# Supplementary material for: LncRNA DANA1 promotes drought tolerance and histone deacetylation of drought responsive genes in Arabidopsis
Source: EMBO Rep. 2024 Jan 2;25(2):796–812. doi: 10.1038/s44319-023-00030-4 (PMC10897447; doi:10.1038/s44319-023-00030-4)
Supplement: Supplementary file 1 — Appendix [file 44319_2023_30_MOESM1_ESM.pdf]

Appendix for

LncRNA *DANA1* promotes histone deacetylation of drought responsive genes in *Arabidopsis*

Jingjing Cai,[1,5] Yongdi Zhang,[1,5] Reqing He,[1,5] Liyun Jiang,[1,5] Zhipeng Qu,[2] Jinbao Gu,[3] Jun Yang,[1] María Florencia Legascue,[4] Zhen-Yu Wang,[3] Federico Ariel,[4] David L Adelson,[2] Youlin Zhu,[1] and Dong Wang[1,6,*]

Appendix Table of Contents

**Figure**	**Page number**
Appendix Figure S1	3
Appendix Figure S2	4
Appendix Figure S3	5-6
Appendix Figure S4	7
Appendix Figure S5	8
Appendix Figure S6	9-10
Appendix Figure S7	11
Appendix Figure S8	12
Appendix Figure S9	13
Appendix Figure S10	14-15
Appendix Figure S11	16
Appendix Figure S12	17
Appendix Figure S13	18
Appendix Figure S14	19
Appendix Figure S15	20
Appendix Figure S16	21

Appendix Figure S17	22
Appendix Figure S18	23
Appendix Figure S19	24
Appendix Figure S20	25
Appendix Figure S21	26
Appendix Figure S22	27
Appendix Figure S23	28
Appendix Figure S24	29
Appendix Figure S25	30
Appendix Figure S26	31

**Table**	**Page number**
Appendix Table S1	32-36
Appendix Table S2	37

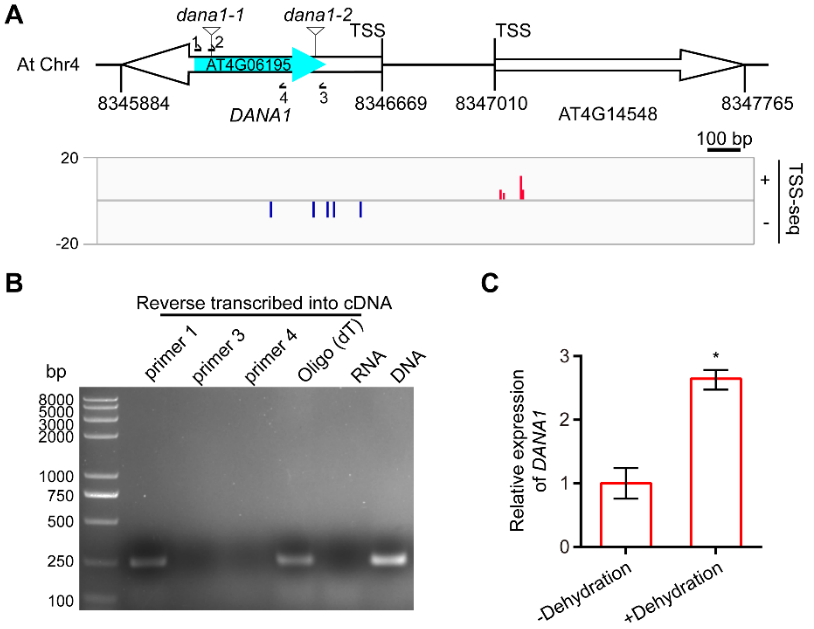

Appendix Figure S1. Detection of transcription direction of *DANA1*.

A Genome browser screenshot of the *DANA1* locus. In TSS-seq (Transcription Start Site sequencing), sense (+) and antisense (-) strands were shown in red and dark blue, respectively. The primer 1, primer 3, primer 4 and oligo (dT) was separately in reverse transcription for producing cDNAs, and primer 2 and primer 4 were used in the PCR reaction presented in (B).

B Strand-specific RT-PCR analysis. RNA and DNA from Col-0 plants were used as negative and positive control, respectively.

C Expression of *DANA1* was induced by dehydration. RNAs were extracted from 10-day-old Col-0 seedlings that were either dehydrated to lose approximately 60% fresh weight or not (-Dehydration), and gene expression level was measured by RT-qPCR normalized against the *UBQ3* gene.

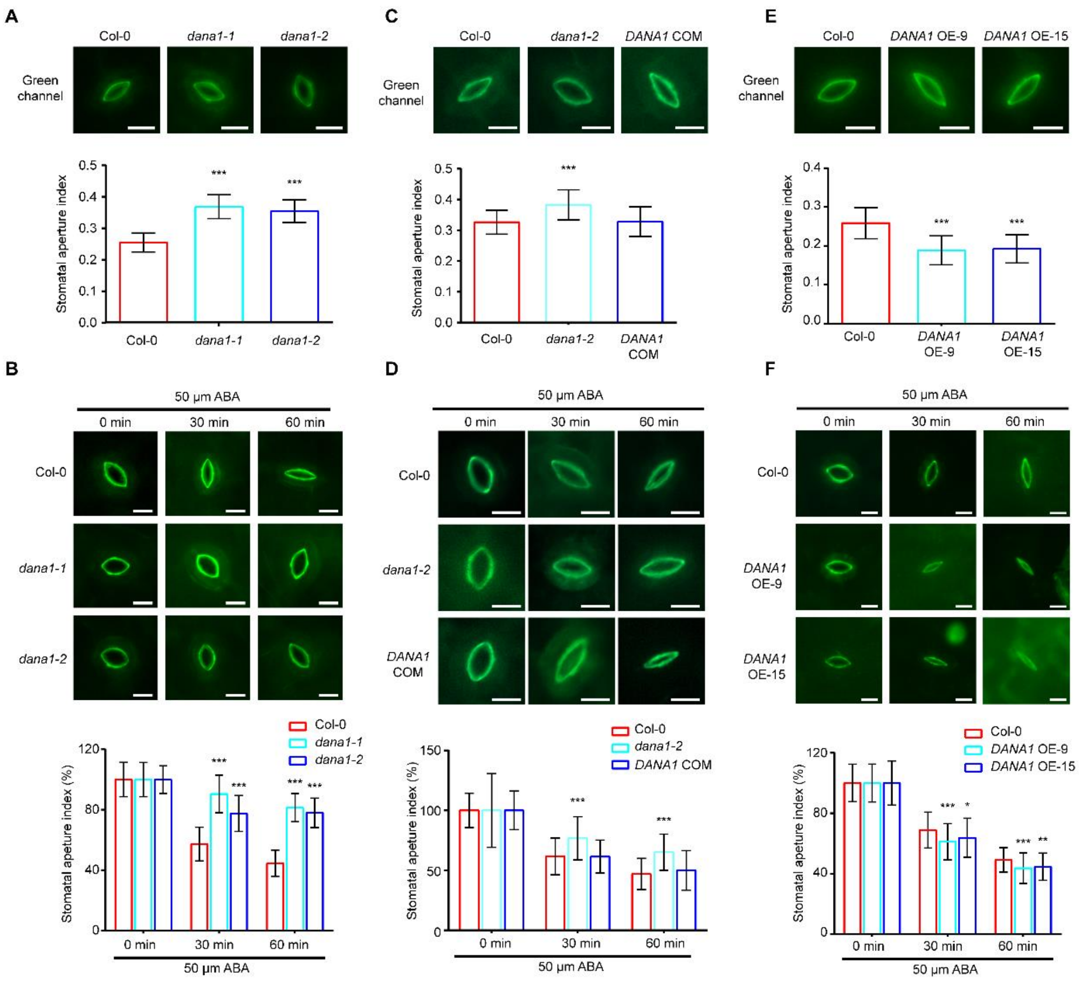

Appendix Figure S2. Stomatal aperture index and ABA-induced stomatal closure in *dana1* mutant, *DANA1* over-expressing and *DANA1* complementation plants.

A, B Mutated *DANA1* increases both stomatal aperture index (A) and ABA-induced stomatal closure (B).

C, D Stomatal aperture index (C) and ABA-induced stomatal closure (D) in *DANA1* COM are similar to Col-0.

E, F Over-expressing *DANA1* decreases both stomatal aperture index (E) and ABA-induced stomatal closure (F).

Data information: Data shown as mean ± SD (n = 60). Asterisks represent significant differences determined by Student's *t*-test (* $P < 0.05$; ** $P < 0.01$; *** $P < 0.001$). Scale bars = 10 μm.

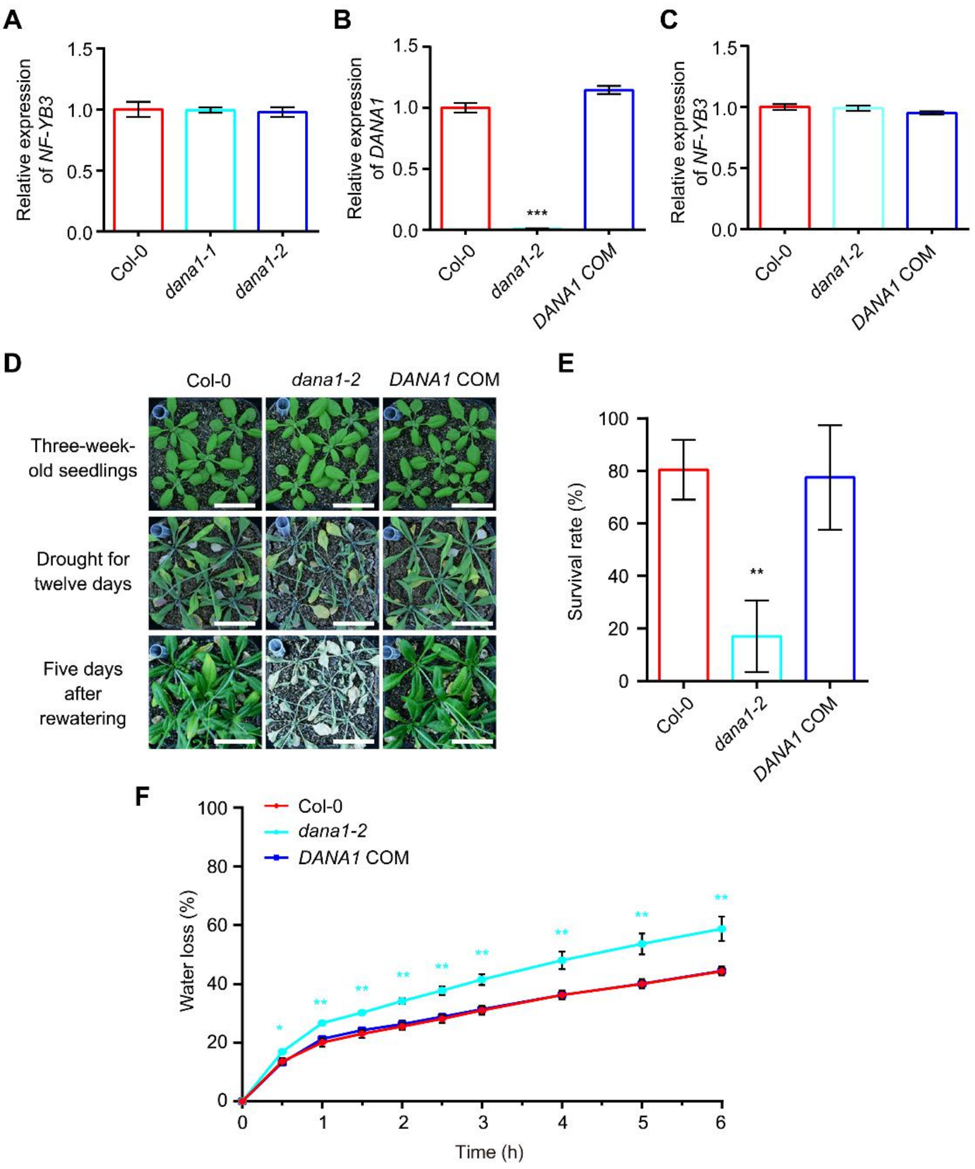

Appendix Figure S3. Drought-tolerant phenotype of *DANA1* complementation plants.

A Quantitative measurement of the transcript levels of *NF-YB3* in Col-0 and the *dana1* mutant plants. *UBQ3* was used as an internal control.
B Quantitative measurement of the transcript levels of *DANA1* in Col-0, *dana1-2* and *DANA1* complementation (*DANA1* COM) plants. *UBQ3* was used as an internal control.
C Quantitative measurement of the transcript levels of *NF-YB3* in Col-0, *dana1-2* and *DANA1* complementation (*DANA1* COM) plants. *UBQ3* was used as an internal control.
D Morphology of seedlings before and after drought stress treatment. Three-week-old Col-0, *dana1-2* and *DANA1* COM plants were subjected to drought stress for twelve days and then rewatered for five days. Scale bars = 3 cm.
E Survival rate after drought treatment (n = 3 biological replates).
F Water loss in detached leaves of three-week-old Col-0, *dana1-2* and *DANA1* COM plants (n = 3 biological replicates, each replicate contains five fully expanded leaves).
Data information: Values shown are means ± SD from three replicates. Asterisks represent significant differences determined by Student's *t*-test (* $P < 0.05$; ** $P < 0.01$;

*** $P < 0.001$).

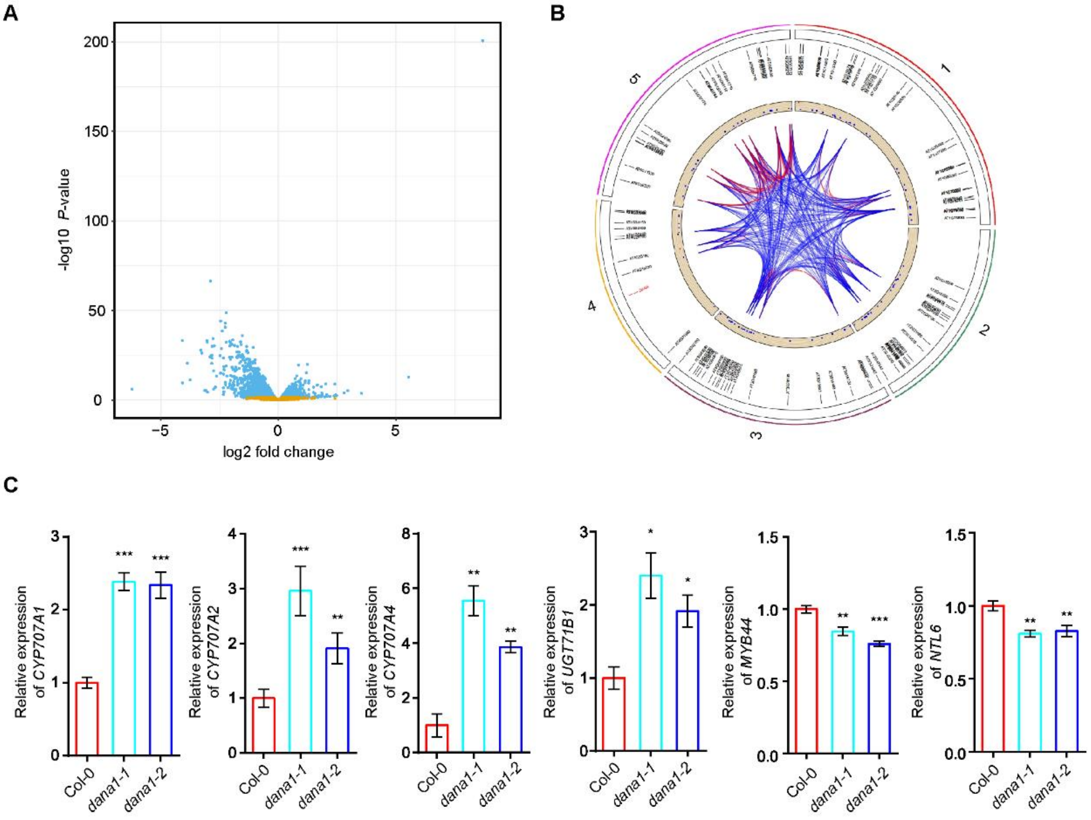

Appendix Figure S4. Gene differential expression analysis of *dana1* mutant using RNA-seq.

A Volcano plot presenting adjusted *p*-value versus gene expression fold change. Blue dots stand for significantly differentially expressed genes (Benjamini-Hochberg method adjusted *P*-value < 0.05).

B Genomic distribution of 100 most significantly differentially expressed genes. Blue and red dots of the scatter plot inside inner track represent down- and up- regulated genes respectively. Links inside the circle plot represent 100 most significantly differentially expressed genes, blue and red lines stand for between- and in-chromosome connections respectively.

C Expression profiles of drought response related genes in *dana1* mutant. *UBQ3* was used as an internal control.

Data information: Asterisks represent significant differences determined by Student's *t*-test (* $P < 0.05$; ** $P < 0.01$; *** $P < 0.001$).

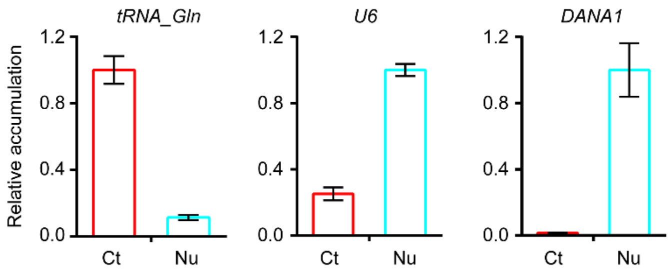

Appendix Figure S5. *DANA1* is mainly enriched in the nucleus.

Relative subcellular distributions of *DANA1* transcripts. *U6* RNA and Glutamine tRNA (*tRNA_Gln*) were used as nuclear and cytoplasmic RNA controls, respectively. Nu: nucleus; Ct: cytoplasm.

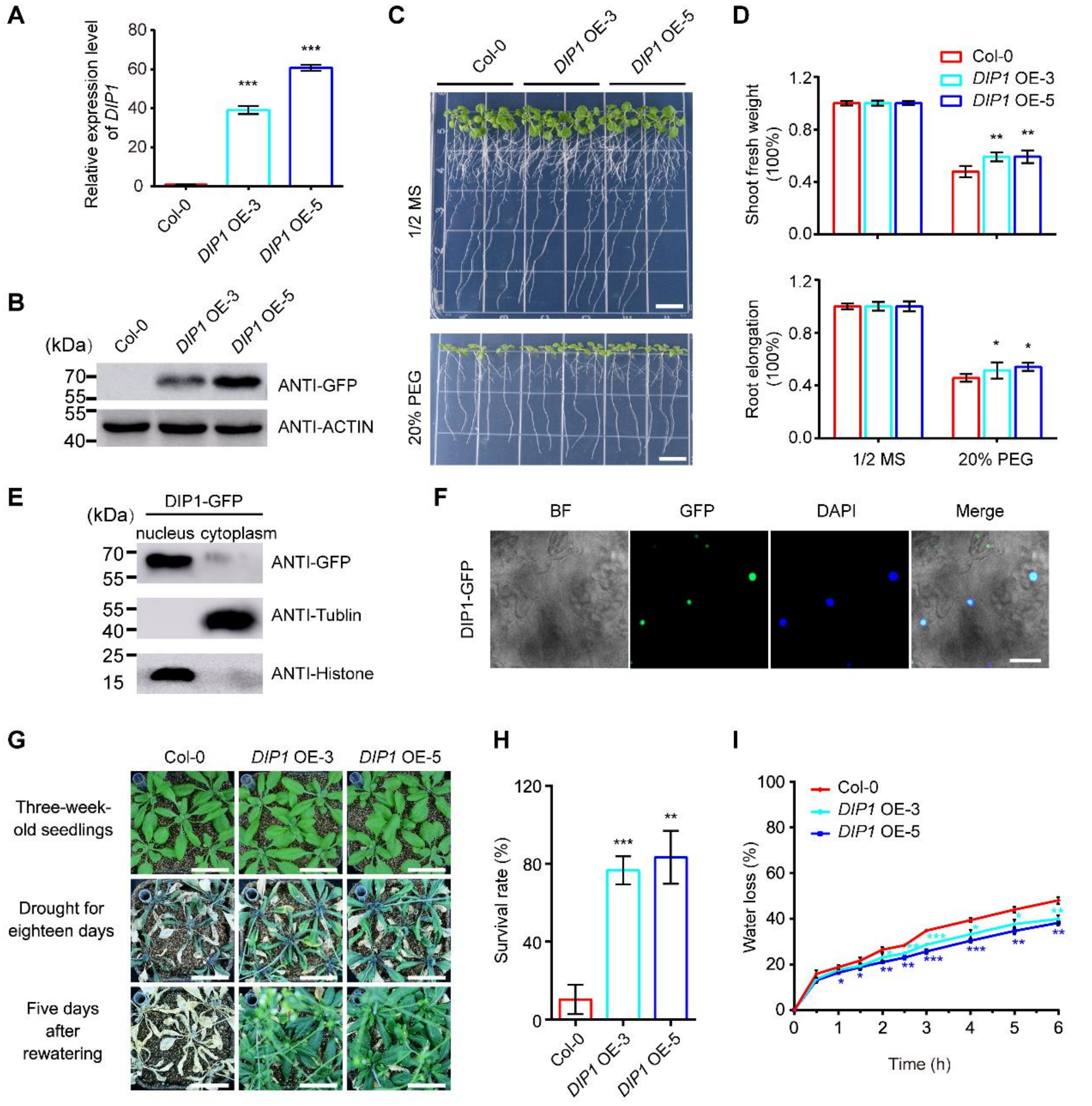

Appendix Figure S6. Drought-tolerant phenotype of *DIP1* over-expressing plants.

A, B Detection on the transcript levels (A) and protein levels (B) of *DIP1* in *DIP1* over-expressing transgenic lines. *DIP1* OE-3 and *DIP1* OE-5 are Col-0 plants transformed with *UBQ10: DIP1-GFP*. *DIP1* OE-5 has been used in RIP assay presented in Fig. 3D. ACTIN is shown as a loading control.

C The *DIP1* over-expressing lines are insensitive to PEG treatment. Scale bars = 1 cm.

D Root length and fresh weight of seedlings shown in (C).

E Immunoblot analyses showing the nucleus and cytoplasmic distributions of DIP1-GFP protein.

F Subcellular localization of DIP1-GFP in rosette leaves of 3-week-old *DIP1* OE-5 plants. Scale bar = 20 µm.

G Drought-tolerance assay. Col-0, *DIP1* OE-3 and *DIP1* OE-5 plants grown under normal growth conditions for three weeks were subjected to drought stress for eighteen days and then rewatered for three days. Scale bars = 3 cm.

H Survival rate after drought treatment (n = 3 biological replates).

I Water loss in detached leaves of three-week-old Col-0, *DIP1* OE-3 and *DIP1* OE-5 plants.

Data information: Values shown are means ± SD from three biological replicates.

Asterisks represent significant differences determined by Student's *t*-test (* $P < 0.05$; ** $P < 0.01$; *** $P < 0.001$).

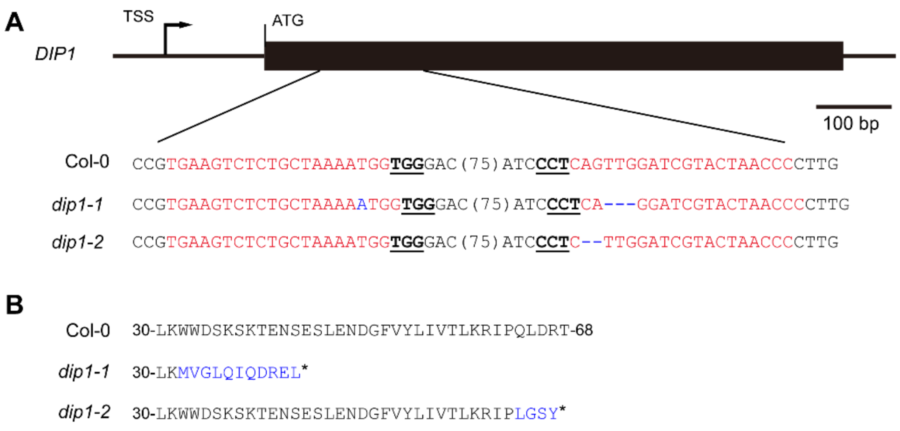

Appendix Figure S7. Targeted mutagenesis of *DIP1* in *Arabidopsis* using CRISPR/Cas9 system.

A CRISPR/Cas9-induced *DIP1* gene mutations at the target site in the mutants. The protospacer of the sgRNA and protospacer-adjacent motif (PAM) sequences are highlighted in red and bold underlined, respectively. Numbers in parentheses indicate gap length.

B Amino acid sequence alignment of wild type DIP1 protein and the *dip1* mutants. Both nucleotide and amino acid changes in *dip1* mutants are highlighted in blue, and asterisks correspond to stop codons.

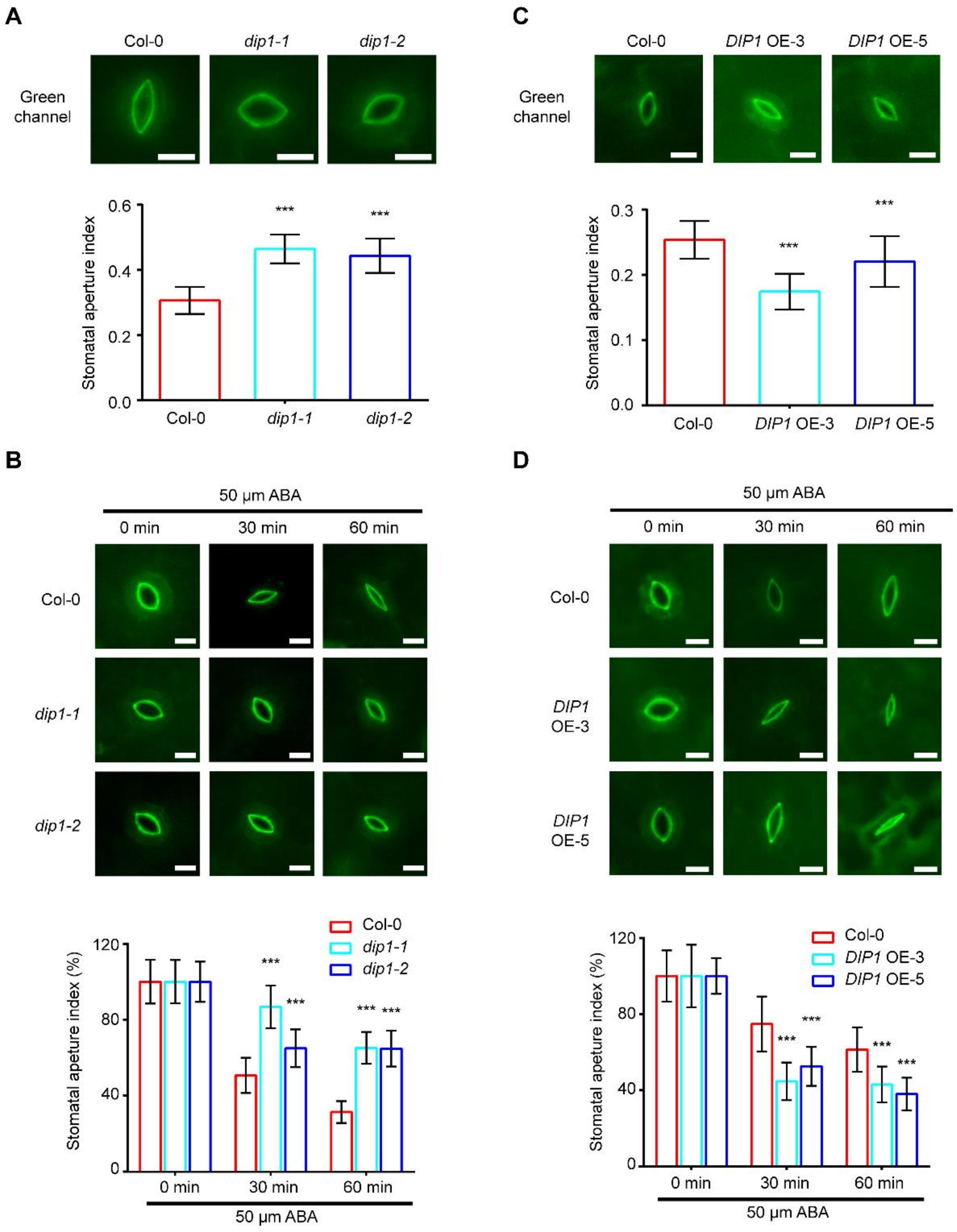

Appendix Figure S8. Stomatal aperture index and ABA-induced stomatal closure in *dip1* mutant and *DIP1* over-expressing plants.

A, B Mutated *DIP1* increases both stomatal aperture index (A) and ABA-induced stomatal closure (B).

C, D Over-expressing *DIP1* decreases both stomatal aperture index (C) and ABA-induced stomatal closure (D).

Data information: Data shown as mean ± SD (n = 60). Asterisks represent significant differences determined by Student's *t*-test (*** $P < 0.001$). Scale bars = 10 µm.

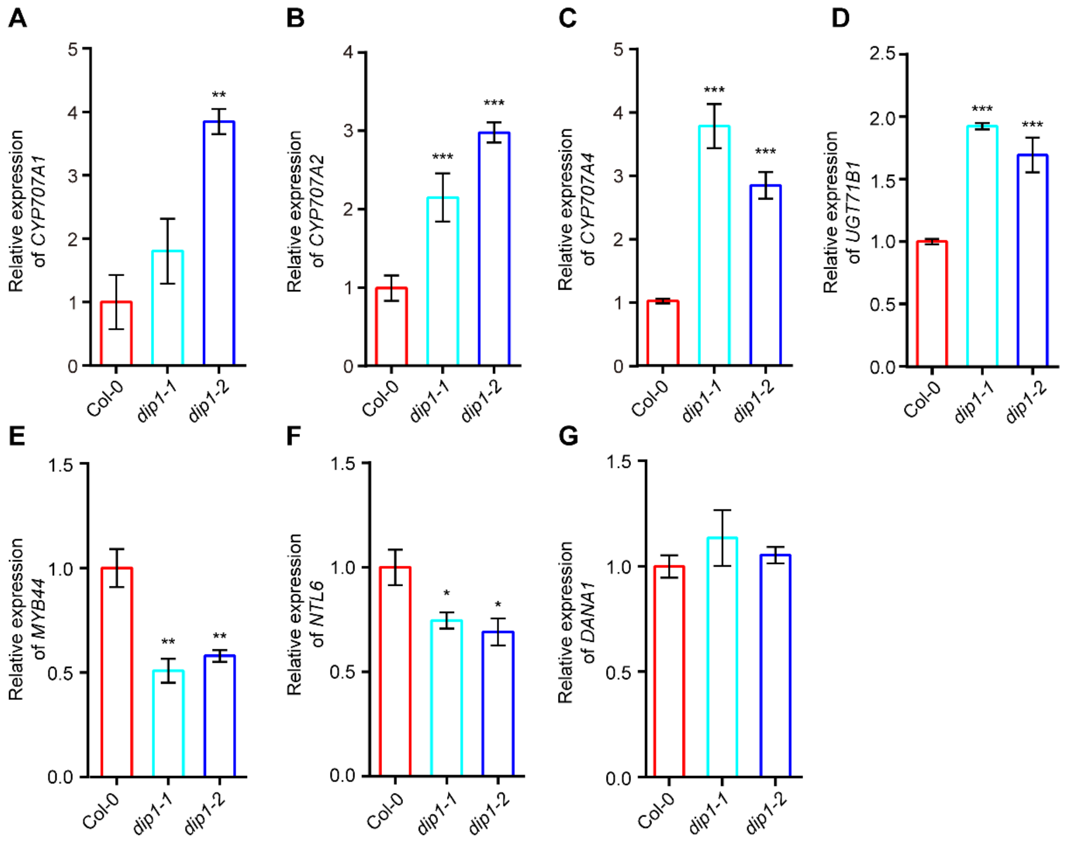

Appendix Figure S9. Quantitative measurement of the transcript levels of drought response related genes (A-F) and *DANA1* (G) in *dip1* mutant. *UBQ3* was used as an internal control.

Data information: Values shown are means ± SD from three replicates. Asterisks represent significant differences by Student's *t*-test (* $P < 0.05$; ** $P < 0.01$; *** $P < 0.001$).

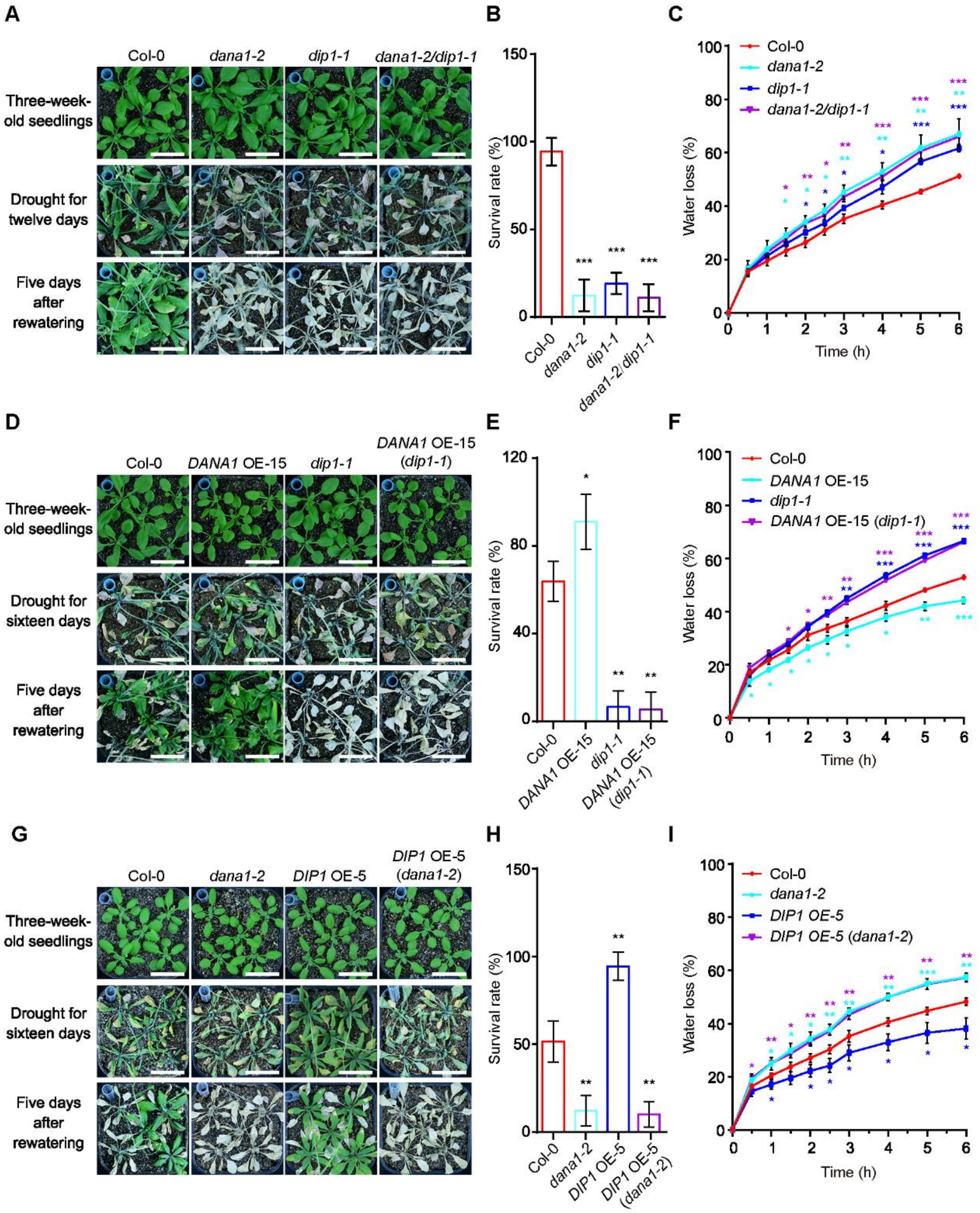

Appendix Figure S10. Genetic interaction of *DANA1* with *DIP1*.

A Morphology of seedlings before and after drought stress treatment. Three-week-old Col-0, *dana1-2*, *dip1-1* and *dana1-2*/*dip1-1* plants were subjected to drought stress for sixteen days and then rewatered for five days. Scale bars = 3 cm.
B Survival rate after drought treatment (*n* = 3 biological replates).
C Water loss in detached leaves of three-week-old Col-0, *dana1-2*, *dip1-1* and *dana1-2*/*dip1-1* plants (*n* = 3 biological replicates, each replicate contains five fully expanded leaves).
D Morphology of seedlings before and after drought stress treatment. Three-week-old Col-0, *DANA1* OE-15, *dip1-1* and *DANA1* OE-15 (*dip1-1*) plants were subjected to drought stress for sixteen days and then rewatered for five days. Scale bars = 3 cm.
E Survival rate after drought treatment (*n* = 3 biological replates).

F Water loss in detached leaves of three-week-old Col-0, *DANA1* OE-15, *dip1-1* and *DANA1* OE-15 (*dip1-1*) plants (n = 3 biological replicates, each replicate contains five fully expanded leaves).

G Morphology of seedlings before and after drought stress treatment. Three-week-old Col-0, *dana1-2*, *DIP1* OE-5 and *DIP1* OE-5 (*dana1-2*) plants were subjected to drought stress for sixteen days and then rewatered for five days. Scale bars = 3 cm.

H Survival rate after drought treatment (n = 3 biological replates).

I Water loss in detached leaves of three-week-old Col-0, *dana1-2*, *DIP1* OE-5 and *DIP1* OE-5 (*dana1-2*) plants (n = 3 biological replicates, each replicate contains five fully expanded leaves).

Data information: Values shown are means ± SD from three biological replicates.

Asterisks represent significant differences by Student's t-test (* $P < 0.05$; ** $P < 0.01$; *** $P < 0.001$).

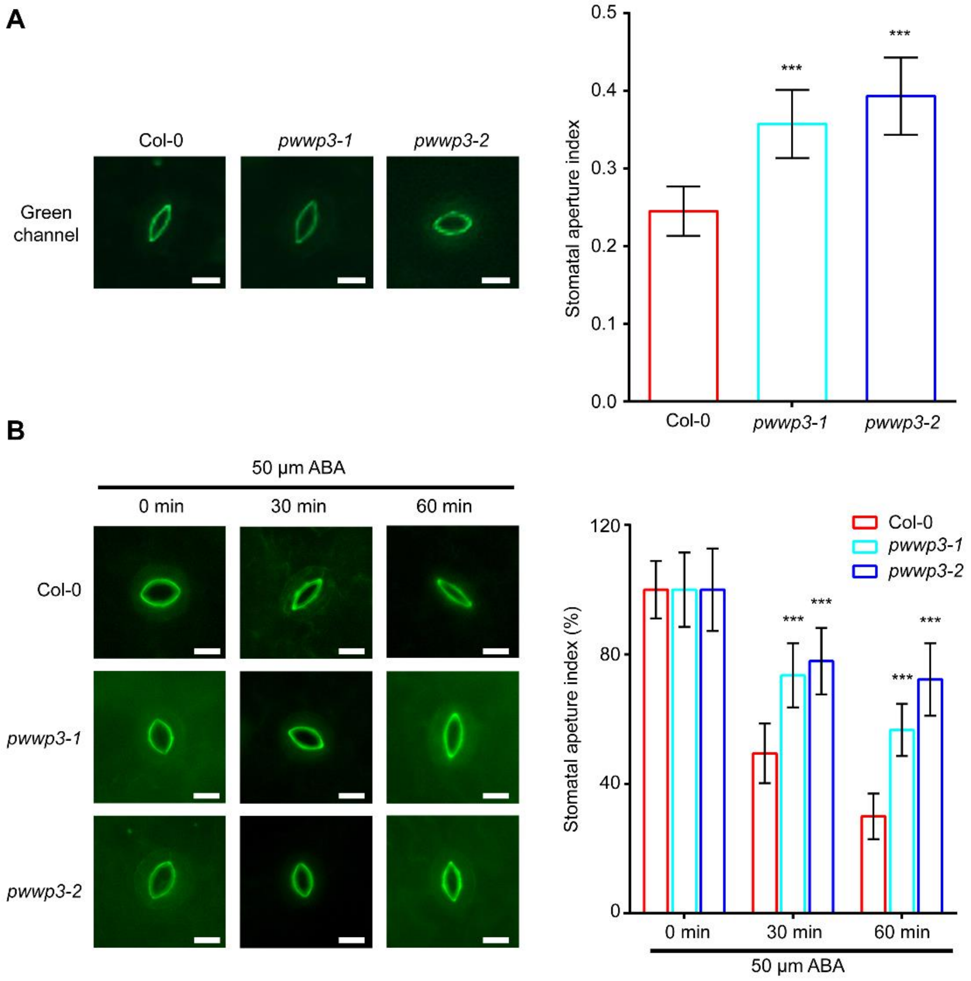

Appendix Figure S11. Mutated *PWWP3* increases both stomatal aperture index (A) and ABA-induced stomatal closure (B).

Data shown as mean ± SD (n = 60). The average of stomatal aperture index of Col-0 and *pwwp3* mutant plants in the presence or absence of ABA shown as a percentage relative to the average of stomatal aperture index of Col-0 and *pwwp3* mutant plants not exposed to ABA, which was defined as 100%.

Data information: Asterisks represent significant differences determined by Student's *t*-test (*** $P < 0.001$). Scale bars = 10 μm.

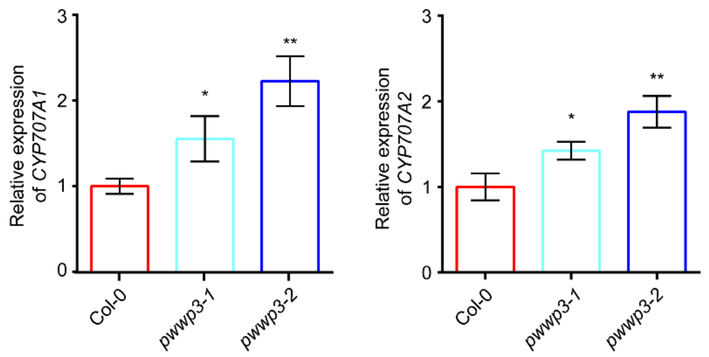

Appendix Figure S12. Quantitative measurement of the transcript levels of *CYP707A1* and *CYP707A2* in Col and the *pwwp3* mutant. *UBQ3* was used as an internal control.

Data information: Asterisks represent significant differences by Student's *t*-test (* $P < 0.05$; ** $P < 0.01$).

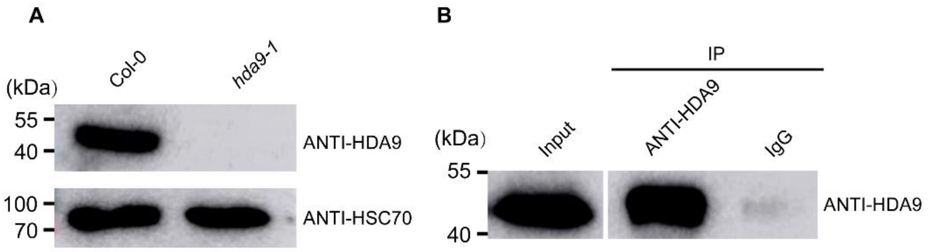

Appendix Figure S13. Validation of the anti-HDA9 antibody.

A Validation of anti-HDA9 antibody by immunoblotting of proteins extracted from Col-0 and *hda9-1* mutant plants. HSC70 is shown as a loading control

B Purification of HDA9 was validated by western blot.

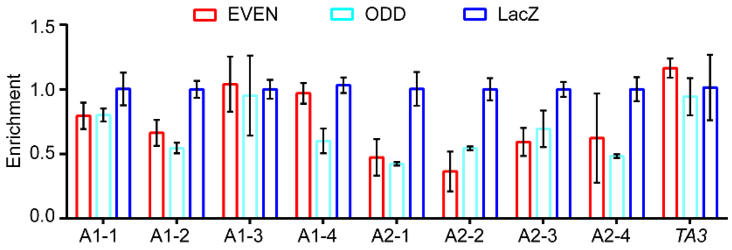

Appendix Figure S14. Mutated *DIP1* impairs the association of *DANA1* to DNA at the *CYP707A1* and *CYP707A2* loci.

Data from ChIRP-qPCR are represented relative to the background level of DNA precipitation (*PP2A*), and the *TA3* locus was used as the negative control.

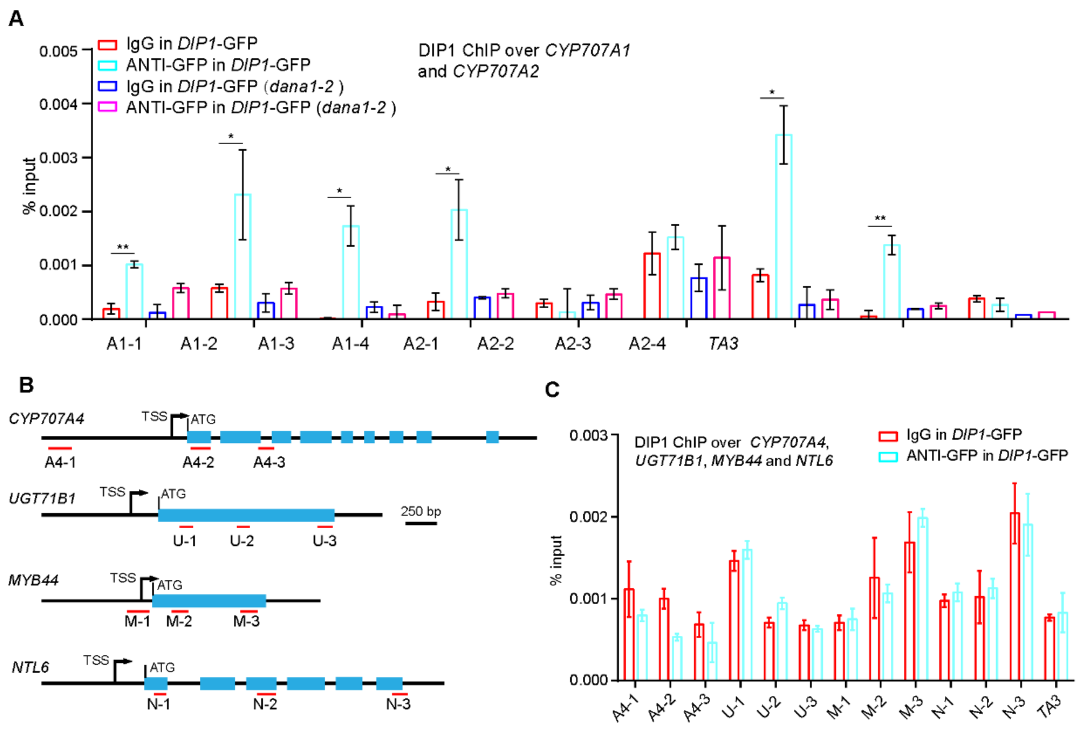

Appendix Figure S15. Mutated *DANA1* impairs the association of *DIP1* to DNA at the *CYP707A1* and *CYP707A2* loci.

A Mutated *DANA1* impairs the association of DIP1 to DNA at the *CYP707A1* and *CYP707A2* loci. The *TA3* locus was used as the negative control.

B Gene structure of *CYP707A4*, *UGT71B1*, *MYB44* and *NTL6*, indicating exons (boxes) and introns (lines). The locations of the gene regions analyzed by ChIP-qPCR are marked.

C The association of DIP1 to DNA at the *CYP707A4*, *UGT71B1*, *MYB44* and *NTL6* loci. The *TA3* locus was used as the negative control.

Data information: Asterisks represent significant differences by Student's *t*-test (* $P < 0.05$; ** $P < 0.01$; *** $P < 0.001$).

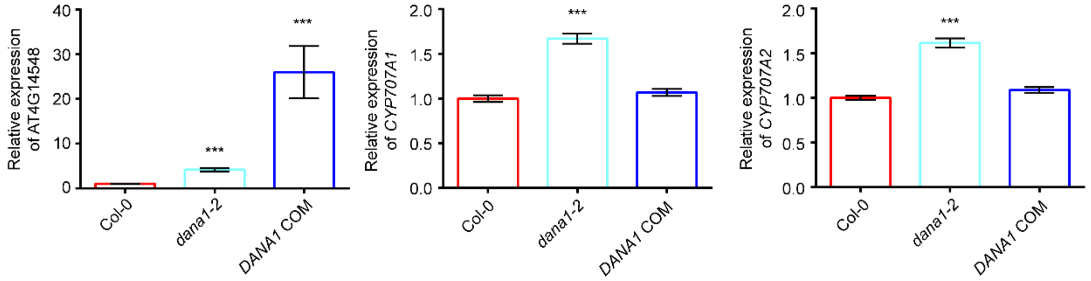

Appendix Figure S16. Quantitative measurement of the transcript levels of AT4G14548, *CYP707A1* and *CYP707A2* in Col-0, *dana1-2* and *DANA1* COM plants. *UBQ3* was used as an internal control.

Data information: Asterisks represent significant differences by Student's *t*-test (*** $P < 0.001$).

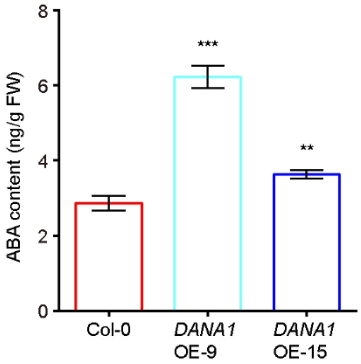

Appendix Figure S17. Measurement of ABA contents in seedlings. The ABA contents were measured in 10-day-old Col-0 and *DANA1*-overexpression seedlings. Data information: Asterisks represent significant differences by Student’s t-test (** $P < 0.01$; *** $P < 0.001$, Student's *t*-test).

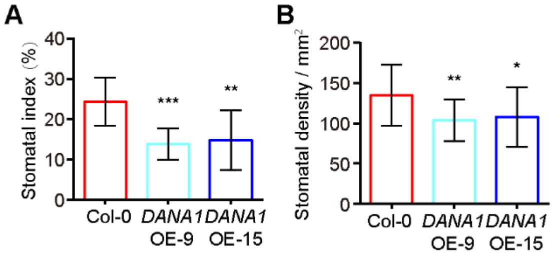

Appendix Figure S18. Both stomatal index (A) and stomatal density (B) are decreased in *DANA1* over-expressing plants.

Data information: Values shown are means ± SD (n = 15). Asterisks represent significant differences determined by Student's t-test (* $P < 0.05$; ** $P < 0.01$; *** $P < 0.001$).

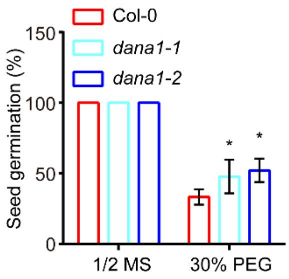

Appendix Figure S19. Seed germination of Col-0, *dana1-1* and *dana1-2* mutants.

Seeds (30 per genotype) were grown on half MS medium supplemented with 30% (w/v) PEG. Seeds were stratified at 4℃ for 3 days, and germinations of seeds were determined when they had grown for 2 days. Seeds were considered as germinated once radicle penetrated the seed coat. Values shown are means ± SD from three replicates. Asterisks represent significant differences determined by Student's *t*-test (* $P < 0.05$).

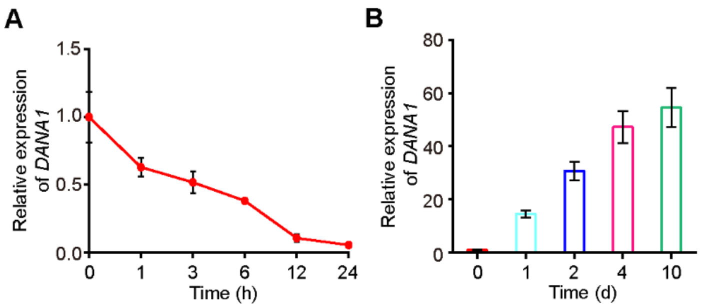

Appendix Figure S20. Quantitative measurement of the transcript levels of *DANA1* during pre- (A) and post-germination (B) stages.

A The RT-qPCR was performed using dry seed and imbibed seed at the designated time points. For seed imbibition, it was carried out as the previous study (Liu *et al*., 2009), and seeds were sown and imbibed on filter paper moistened with water at 22°C under continuous light.

B The RT-qPCR was performed using dry seed and seed that were sown on 1/2 MS media, stratified for 3 days at 4°C in the dark and then grown at 22°C with a 16-h light/8-h dark photoperiod at the designated time points. Expression level of *DANA1* in dry seed was designed as 1, and *UBQ3* was used as an internal control.

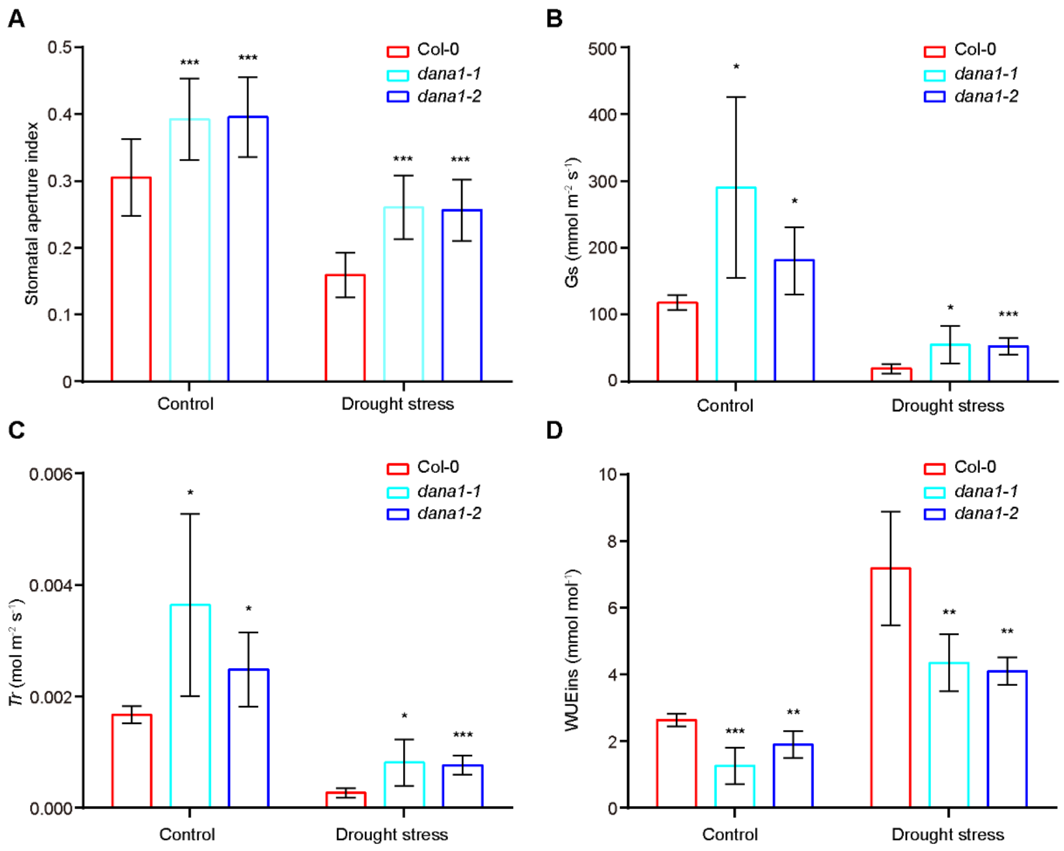

Appendix Figure S21. Stomatal aperture index (A), stomatal conductance (B), transpiration rate (C) and water use efficiency (D) in the rosette leaves of four-week-old Col-0, *dana1-1* and *dana1-2* mutants grown under normal and drought stress conditions.

Data information: Values shown are means ± SD from three replicates. Asterisks represent significant differences determined by Student's *t*-test (* $P < 0.05$; ** $P < 0.01$; *** $P < 0.001$).

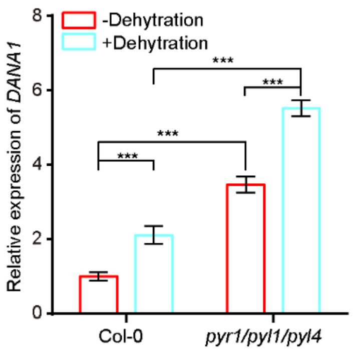

Appendix Figure S22. Expression of *DANA1* in WT and an ABA-insensitive mutant *pyr1*/*pyl1*/*pyl4*. *UBQ3* was used as an internal control.

Data information: Asterisks represent significant differences determined by Student's *t*-test (*** $P < 0.001$).

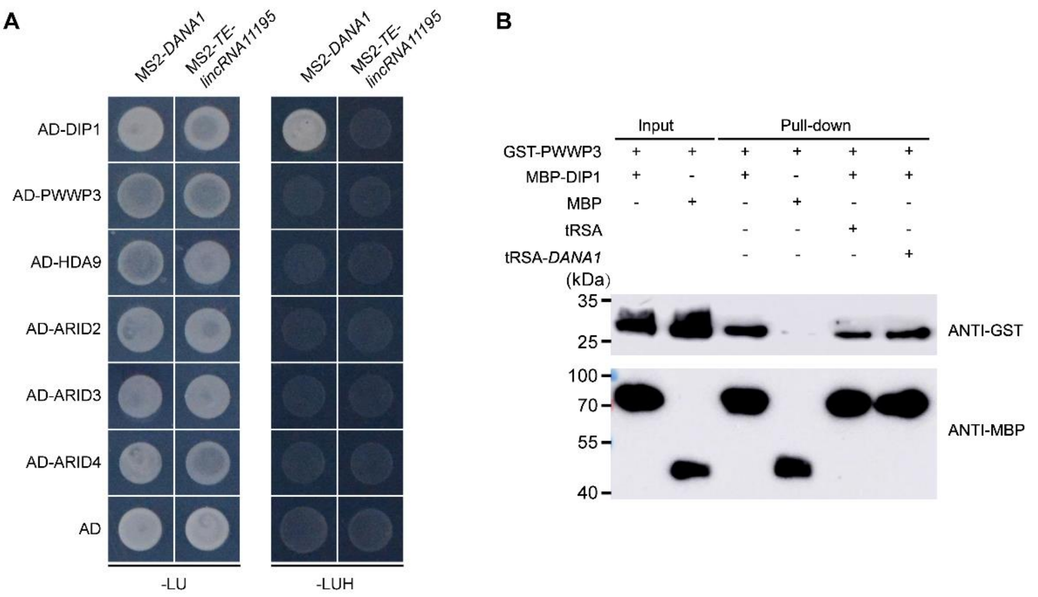

Appendix Figure S23. *DANA1* does not influence the interaction between DIP1 and PWWP3.

A Tests of *DANA1*-PWWP3, *DANA1*-HDA9 and *DANA1*-ARIDs interactions by yeast three-hybrid assays. Full-length PWWP3, HDA9, ARID2, ARID3 and ARID4 proteins were separately fused with the GAL4 activation domain (AD).

B *In vitro* pull-down assay of GST-PWWP3 and maltose binding protein (MBP)-DIP1 with *in vitro* transcribed tRSA-*DANA1* or tRSA (100 pmol).

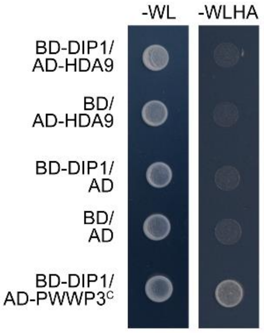

Appendix Figure S24. Tests of DIP1-HDA9 interaction by yeast two-hybrid assays.

Full-length HDA9 protein was fused with the GAL4 activation domain (AD).

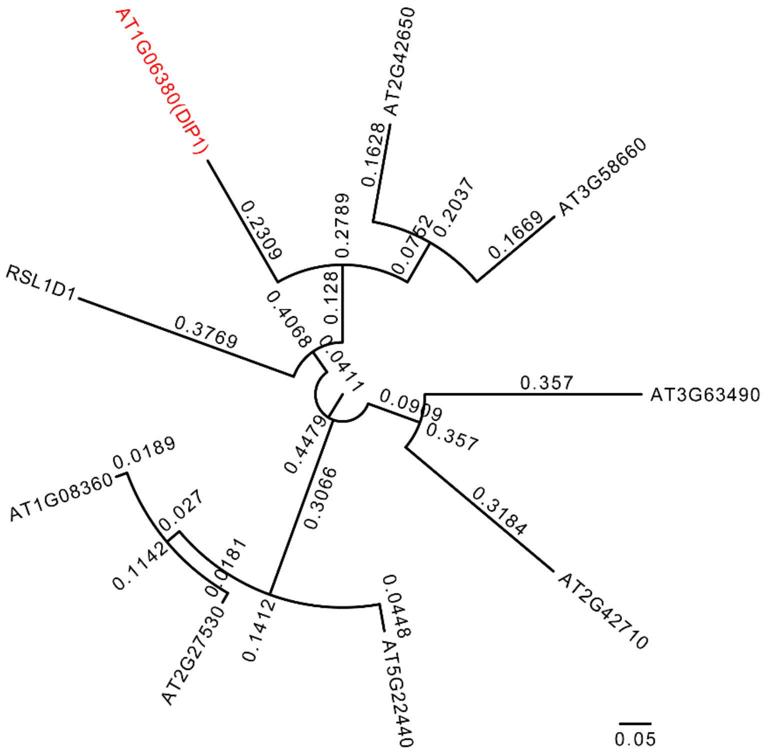

Appendix Figure S25. Phylogenetic tree of ribosomal protein L1P/L10e family.

AT5G22440, AT2G27530 and AT1G08360 are components of 80S ribosome (Barakat, Szick-Miranda et al., 2001). RSL1D1 is a human ribosomal L1 domain-containing protein, which directly interacts with c-MYC protein (Cheng, Yuan et al., 2015). The phylogenetic tree was constructed using a neighbour joining clustering method based on the multiple alignment of amino acid sequences of these genes. The scale bar represents the amino acid substitutions per site.

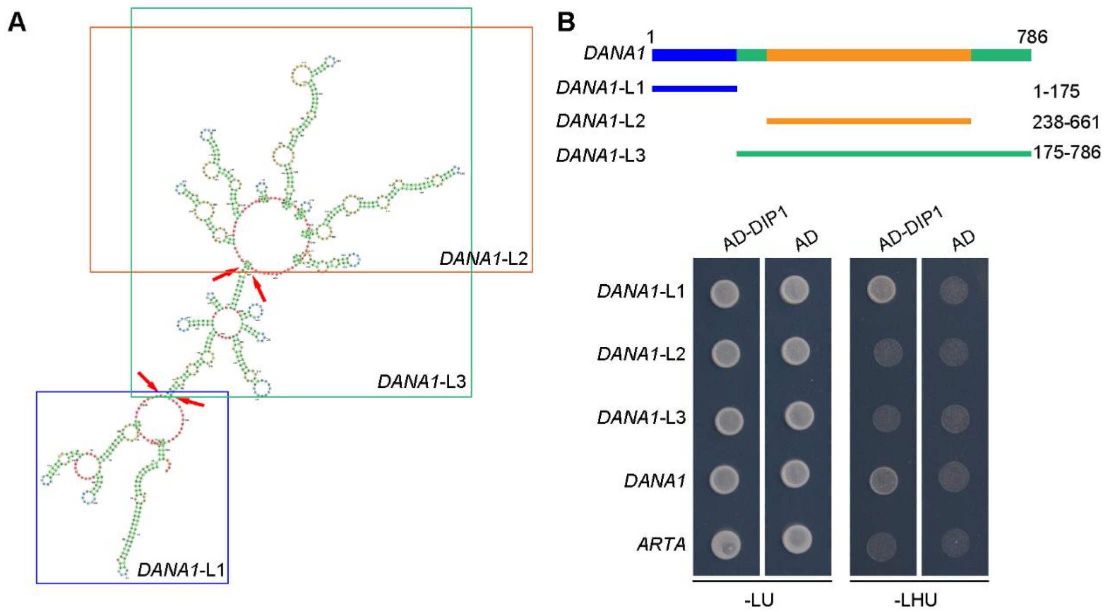

Appendix Figure S26. ***DANA1*-L1 interacts with DIP1 in yeast cells.**
A Secondary structure of the *DANA1* predicted with *RNAfold*.
B Tests of *DANA1*-DIP1 interactions by yeast three-hybrid assays.

References

Barakat A, Szick-Miranda K, Chang IF, Guyot R, Blanc G, Cooke R, Delseny M, Bailey-Serres J (2001) The organization of cytoplasmic ribosomal protein genes in the Arabidopsis genome. *Plant Physiol* 127: 398-415

Cheng Q, Yuan F, Lu F, Zhang B, Chen T, Chen X, Cheng Y, Li N, Ma L, Tong T (2015) CSIG promotes hepatocellular carcinoma proliferation by activating c-MYC expression. *Oncotarget* 6: 4733-44

Liu Y, Shi L, Ye N, Liu R, Jia W, Zhang J (2009) Nitric oxide-induced rapid decrease of abscisic acid concentration is required in breaking seed dormancy in Arabidopsis. *New Phytol* 183: 1030-1042

Appendix Table S1. Primers and probes used in this study.

Primer name	Sequences	Purpose
DANA1-F	ACTTGCTTTAATGTGGCATTGAGGG	T-DNA detection and RT-PCR
DANA1-R	GTTCCGACGAACCTAAATGAAAGTC	
PWWP3-JC-F	GGCTGTCAAGTATGCCCGTAGAG	
PWWP3-JC-R	AGCCCTTCACTGCTTCGTTTATCCTAC	
DANA1-F2	CCCTACCTATTGAATGATTCCAGC	
DANA1-1	AAAGTCTCCATGACAGACTC	
DANA1-2	AAAGATTAAAGAGAAACTCA	
DANA1-3	ATCCGCCTGTCATCAACAAC	
DANA1-4	ACTCTTTTCCAGCTTCGCTC	
PROKII-LB1	ATGGTTCACGTAGTGGGCCATCG	
pDAP101-LBa1	GCCTTTTCAGAAATGGATAAATAGCCTTGCTTCC	
DANA1-QF	CCATCCGCCTGTCATCAAC	RT-qPCR
DANA1-QR	GGACCATGCTTTGTGCTCTGTTCTC	
CYP707A1-QF	TGGCTCCAAAACCCAATACGT	
CYP707A1-QR	CGAATGGCCCATACTGAATC	
CYP707A2-QF	GTTCAAGCCAACTTATCC	
CYP707A2-QR	TAAGGGTAGAATGGTATGG	
CYP707A4-QF	ACCTACCAGGAGATGAAG	
CYP707A4-QR	ACATCAAAGGCGAACTGC	
UGT71B1-QF	TAAAGAAGGAGTACCGTAGAGAT	
UGT71B1-QR	GAACAAACTTCTTTAGAGCACAG	
MYB44-QF	TTTAGAGGTGCGATTGAGG	
MYB44-QR	ATCCGCCACCATTGTTCC	
NTL6-QF	ACCTGCTGTCTCGTCTCC	
NTL6-QR	TAGCCTCATCACAAGCATCACT	
NF-YB3-QF	AGGCGATAAGGAAGGTGGAGGAGGA	
NF-YB3-QR	TTGATGTCCCATCGTAGTCACCATG	
AT4G14548-QF	ATCTACGGGATTTGCTGCTTGTTTT	
AT4G14548-QR	CAACACCCGAAGACCCAAAGGAAAA	
UBQ3-QF	ACGGAAGAACTCTTGCTGAC	
UBQ3-QR	AACCTCAAGGGTGATTGTTT	
MS2-QF	GGTCGACTCTAGAAAACATG	
MS2-QR	AGGATCCAATGAACCCGGG	
ACTIN(*N. benthamiana*)-F	CAAGGAAATCACCGCTTTGG	
ACTIN(*N. benthamiana*)-R	AAGGGATGCGAGGATGGA	
U6-QF	TCCCTTCGGGGACATCCGATA	
U6-QR	AAATTTGGACCATTTCTCGATTTGTGC	
tRNA_Gln-QF	CCACCGCTTCTGCCGTTAAA	
tRNA_Gln-QR	GATCCGAGCCAAATCCGAGA	
MS2-DANA1-F	GACTCTAGAGGATCGCCCGGGGATTTTTTTTGAGATGTC ATGTTGA	Y3H and Y2H assays
MS2-DANA1-R	AGAACTAGTGGATCCCCCGGGAAATCAGGAAAAAATA AAGAATTA	
MS2-DANA1-L1F	GACTCTAGAGGATCGCCCGGGGATTTTTTTTGAGATGTC AT	
MS2-DANA1-L1R	AGAACTAGTGGATCCCCCGGGGGCGGATGGTGCTGGAA TCA	

MS2-DANA1-L2F	GACTCTAGAGGATCGCCCGGGTGTCATCAACAACTTCTACA	
MS2-DANA1-L2R	AGAACTAGTGGATCCCCCGGGAAATCAGGAAAAAATAAAGA	
MS2-DANA1-L3F	GACTCTAGAGGATCGCCCGGGTGTGTTTACGAGGTTTGGCA	
MS2-DANA1-L3R	AGAACTAGTGGATCCCCCGGGGGTGTAGAATTAAAAACAAG	
MS2-TE-lincRNA11195-F	GACTCTAGAGGATCGCCCGGGCTCCTTATCTCTATTATTGT	
MS2-TE-lincRNA11195-R	AGAACTAGTGGATCCCCCGGGCTGACACTTTTCTTTTCTTTTCG	
AD-DIP1-F	GCCATGGAGGCCAGTGAATTCATGAGTACTACTACTACTACGG	
AD-DIP1-R	CAGCTCGAGCTCGATGGATCCAGCAACTGATTGATAAACAGGC	
BD-DIP1-F	ATGGCCATGGAGGCCGAATTCATGAGTACTACTACTACTACGG	
BD-DIP1-R	CCGCTGCAGGTCGACGGATCCAGCAACTGATTGATAAACAGGC	
AD-PWWP3-F	GCCATGGAGGCCAGTGAATTCATGGGTAGTAGTGATGAGCGAAACT	
AD-PWWP3-R	CAGCTCGAGCTCGATGGATCCTCCTGTATTACCTGCAGATGGT	
AD-PWWP3^{c}-F	GCCATGGAGGCCAGTGAATTCGATGTTCCACTAAATGGAGAT	
AD-ARID2-F	CCATGGAGGCCAGTGAATTCATGGAGAATTTGACGGAAAT	
AD-ARID2-R	AGCTCGAGCTCGATGGATCCCTCCAATTGCTCCAGAGGCA	
AD-ARID3-F	CCATGGAGGCCAGTGAATTCATGGTGGATACCGAAATGCA	
AD-ARID3-R	AGCTCGAGCTCGATGGATCCCTGCTCAAACGGAACTCGTA	
AD-ARID4-F	CCATGGAGGCCAGTGAATTCATGATGGCGGATACTGAAATGCAAGA	
AD-ARID4-R	AGCTCGAGCTCGATGGATCCTCACTGCTCAAATGGGACTCGCACAA	
AD-HDA9-F	CCATGGAGGCCAGTGAATTCATGCGTTCCAAGGACAAAAT	
AD-HDA9-R	AGCTCGAGCTCGATGGATCCTGACGCATCGTTATCGTTGT	
GST-DIP1-F	GATCTGGTTCCGCGTGGATCCATGAGTACTACTACTACTACGG	
GST-DIP1-R	CTCGAGTCGACCCGGGAATTCAGCAACTGATTGATAAACAGGC	
tRSA-F	TAATACGACTCACTATAGGGAAAAAAAA	
tRSA-R	GAATTCTTTTTTTTTTTTCTGCAGTG	
tRSA-DANA1-F	CAGAAAAAAAAAAAAGAATTCGATTTTTTTTGAGATGTCATGTTGA	
tRSA-DANA1-R	AGACTGCAGGTCGACAAGCTTAAATCAGGAAAAAATAAAGAATTA	RNA pull-down and protein pull-down assays
DANA1-T-R	AAATCAGGAAAAAATAAAGAATTA	
MBP-DIP1-F	CTGTACTTCCAATCCAATATGAGTACTACTACTACTACGGCTG	
MBP-DIP1-R	CCGTTATCCACTTCCAATAGCAACTGATTGATAAACAGGCAAT	
GST-PWWP3-F	TTCCAGGGGCCCCTGGGATCCATGGGTAGTAGTGATGAGCGAAACT	
GST-PWWP3-R	GACCCGGGAATTCGGGGATCCTCCTGTATTACCTGCAGATGGT	
DANA1-TriFC-F	GGGGACTCTTGACCATGGAGGCCTGATTTTTTTTGAGATGTCATGTTGA	

DANA1-TriFC-R	ACGGCTCGAGCGGCCGCAGGCCTAAATCAGGAAAAAATAAAGAATTA	TriFC and BiFC assays
DIP1-TriFC-F	ACACGGGGGACTCTTGAAGGCCTATGAGTACTACTACTACTACGG	
DIP1-TriFC -R	GCCCTTGCTCACCATAGGCCTAGCAACTGATTGATAAACAGGC	
MSCP-TriFC-F	CAAGGCTAGCGAATTCCACGTGGCTTCTAACTTTACTCAGTTCGT	
MSCP-TriFC-R	GGTCACCTGTAATTCACACGTGTTAGTAGATGCCGGAGTTGGCCG	
DIP1-YC-F	CCTGGCGCGCCACTAGTGGATCCATGAGTACTACTACTACTAC	
DIP1-YC-R	GGAGCGGTACCCTCGAGGTCGACAGCAACTGATTGATAAACAG	
PWWP3-YN-F	CCTGGCGCGCCACTAGTGGATCCATGGGTAGTAGTGATGAGCG	
PWWP3-YN-R	GGAGCGGTACCCTCGAGGTCGACTCCTGTATTACCTGCAGATG	
PP2A-QF	TATCGGATGACGATTCTTCGTGCAG	RIP assay
PP2A-QR	GCTTGGTCGACTATCGGAATGAGAG	
DANA1-QF1	ATAATAATAGCTCGTACAGC	
DANA1-QR1	ACAAAGCATGGTCCTTCA	
DIP1-sgRNA1F	TGATTGGAAGTCTCTGCTAAAATGG	CRISPR/Cas9 assay
DIP1-sgRNA1R	AAACCCATTTTAGCAGAGACTTCCA	
DIP1-sgRNA2F	TGATTGGGTTAGTACGATCCAACTG	
DIP1-sgRNA2R	AAACCAGTTGGATCGTACTAACCCA	
DIP1-CX-F	TTGCTCAGAACCCTAAT	
DIP1-CX-R	TCCATCTACCAGGAACT	
CYP707A1-1F	GTAGACTTTACCCCACAT	ChIP and ChIRP qPCR assays
CYP707A1-1R	GGTTAATAATTCGTTCTGTT	
CYP707A1-2F	TCCGCCTTGTTTCTCACT	
CYP707A1-2R	TCCGACGTAAGGCCAACC	
CYP707A1-3F	TTATCACGCTAAACTCAG	
CYP707A1-3R	GAGCGATGGATTCAATAT	
CYP707A1-4F	TGAATCTCCCTGGAACAC	
CYP707A1-4R	GATTACTCCGATTATGTTGT	
CYP707A2-1F	AAGTGTAGTGTGGGGTTAGC	
CYP707A2-1R	CGCAGTACTATTTATGTGGT	
CYP707A2-2F	CAAAGACTACGGCTACCT	
CYP707A2-2R	CGAGTGGCGAAGAAGGAA	
CYP707A2-3F	GTTCAAGCCAACTTATCC	
CYP707A2-3R	TAAGGGTAGAATGGTATGG	
CYP707A2-4F	TTTGGCTTCAATGTCTAAC	
CYP707A2-4R	GGAGAATTGGCTCAGGGT	
CYP707A4-1F	CACCCGTAAATGTTGTCA	
CYP707A4-1R	CAACGGCATGTCGGTATT	
CYP707A4-2F	ATCCTCATCTTATGCTTGC	
CYP707A4-2R	TTTGCTTGGAGGTGAAGA	
CYP707A4-3F	ACCTACCAGGAGATGAAG	
CYP707A4-3R	ACATCAAAGGCGAACTGC	
MYB44-1F	AAAACCGCTTGCGTGGAA	
MYB44-1R	CTTTAGCAATCTTCTCCT	

MYB44-2F	CGCCGCAAGTTGAGCATC	
MYB44-2R	TTCTTCACGGCGTTGTCC	
MYB44-3F	TTTAGAGGTGCGATTGAGG	
MYB44-3R	ATCCGCCACCATTGTTCC	
NTL6-1F	TAATCCGTTACTATCTCCG	
NTL6-1R	AATCCCAAGGTTCCCATT	
NTL6-2F	ACCTGCTGTCTCGTCTCC	
NTL6-2R	CGGTTGTAGCCTCATCAC	
NTL6-3F	TTAGGGCGGCAGTAGTTG	
NTL6-3R	GGGCCGGTTCAAGCACTA	
UGT71B1-1F	ACCGTCTCCGCTACATCC	
UGT71B1-1R	AGCGACCTTGGACACGAC	
UGT71B1-2F	ATGGAACCTCAGGCGTTGA	
UGT71B1-2R	GCCGCTAGATTCTAAGTC	
UGT71B1-3F	GGAGCAGGATAGCAAGAT	
UGT71B1-3R	GACCACGTCTTGAACAAA	
TA3-F	GATTCTTACTGTAAAGAACATGGCATTGAGAGA	
TA3-R	TCCAAATTTCCTGAGGTGCTTGTAACC	
DANA1-OE-F	TATGACCATGATTACGAATTCTCAACATGGTGGAGCACGACACAC	Overexpression and complementation
DANA1-OE-R	CAGGTCGACTCTAGAGGATCCCCGATCTAGTAACATAGATGACACC	
DANA1-COM-F	TATGACCATGATTACGAATTCGCGCATAATGAAAGGATCCGAAGC	
DANA1-COM-R	CAGGTCGACTCTAGAGGATCCCGTAACCTTTGATCTTTGATCTTTG	
DIP1-OE-F	GTTTTTCTGATTAACAGAATTCGATGAGTACTACTACTACTACGG	
DIP1-OE-R	TCTCCATGGTCTAGAGGATCCAGCAACTGATTGATAAACAGGC	
PWWP3-MYC-F	GCTTGGGCGACCTCACCGAATTCATGGGTAGTAGTGATGAGCG	
PWWP3-MYC-R	AGTTATCTAGATCCGGTGGATCCTCCTGTATTACCTGCAGATG	
DANA1_ChIRP_probe_1	CCTCAATGCCACATTAAAGC-3'biotin	ChIRP biotinylated probes
DANA1_ChIRP_probe_2	GCAAGTGGTGAATTCCTAGA-3'biotin	
DANA1_ChIRP_probe_3	TGTAGGCAACAACTTTACCA-3'biotin	
DANA1_ChIRP_probe_4	TGGTGCTGGAATCATTCAAT-3'biotin	
DANA1_ChIRP_probe_5	AGCAGTGCAATGGAGATGTC-3'biotin	
DANA1_ChIRP_probe_6	GTGGTACTCTTATGCCAAAC-3'biotin	
DANA1_ChIRP_probe_7	GCTGGAAAAGAGTTGTGGCG-3'biotin	
DANA1_ChIRP_probe_8	CTCTGTTCTCACAACATTGA-3'biotin	
DANA1_ChIRP_probe_9	ATAGGAGAAGCTTTTACCCT-3'biotin	
DANA1_ChIRP_probe_10	GGATCTCTCCAAAACATTCC-3'biotin	
DANA1_ChIRP_probe_11	TCCATCGAAGTAGAGCTTTC-3'biotin	
DANA1_ChIRP_probe_12	AATAGCTCGTACAGCTAGTG-3'biotin	
DANA1_ChIRP_probe_13	TCCATGACAGACTCCATATT-3'biotin	
DANA1_ChIRP_probe_14	ATAGGAAGTTCCGACGAACC-3'biotin	
DANA1_ChIRP_probe_15	AGAAGATCATACGGTGTAGA-3'biotin	
DANA1_ChIRP_probe_16	ACCAAACTGCTTTCCATTAT-3'biotin	
LacZ_ChIRP_probe_1	CCAGTGAATCCGTAATCATG-3'biotin	
LacZ_ChIRP_probe_2	AATGTGAGCGAGTAACAACC-3'biotin	

LacZ_ChIRP_probe_3	AATAATTCGCGTCTGGCCTT-3'biotin	
LacZ_ChIRP_probe_4	AATTCAGACGGCAAACGACT-3'biotin	
LacZ_ChIRP_probe_5	ATCTTCCAGATAACTGCCGT-3'biotin	
LacZ_ChIRP_probe_6	GCTGATTTGTGTAGTCGGTT-3'biotin	
LacZ_ChIRP_probe_7	AACTGTTACCCGTAGGTAGT-3'biotin	
LacZ_ChIRP_probe_8	TTTCGACGTTCAGACGTAGT-3'biotin	
LacZ_ChIRP_probe_9	ACCATTTTCAATCCGCACCT-3'biotin	
LacZ_ChIRP_probe_10	TTCATCAGCAGGATATCCTG-3'biotin	

Appendix Table S2. The list of *DANA1*-interacting proteins identified by a yeast three-hybrid assay.

Number	Gene ID	Gene description
1	AT1G06380	Ribosomal protein L1p/L10e family (DIP1)
2	AT1G49650	alpha/beta-Hydrolases superfamily protein
3	AT1G58470	Encodes an mRNA-binding protein that contains two RNA recognition motifs (RRMs) and is expressed in proliferating tissues
4	AT3G12470	Polynucleotidyl transferase, ribonuclease H-like superfamily protein
5	AT3G15080	Polynucleotidyl transferase, ribonuclease H-like superfamily protein
6	AT3G07050	Arabidopsis NSN1 encodes a nucleolar GTP- binding protein and is required for maintenance of inflorescence meristem identity and floral organ development
7	AT3G54770	Encodes a putative RNA binding protein that is localized in the nucleus and affects ABA-regulated seed germination of Arabidopsis
8	AT4G04890	Arabidopsis thaliana protodermal factor 2 (PDF2)
9	AT5G14610	DEAD box RNA helicase family protein
10	AT5G46250	AtLARP6a, La related protein 6a
11	AT5G52470	ATFIB1
12	AT5G60980	Nuclear transport factor 2 (NTF2) family protein with RNA binding (RRM-RBD-RNP motifs) domain-containing protein
