# Supplementary figures and images for: LncRNA DANA1 promotes drought tolerance and histone deacetylation of drought responsive genes in Arabidopsis
Source: EMBO Rep. 2024 Jan 2;25(2):796–812. doi: 10.1038/s44319-023-00030-4 (PMC10897447; doi:10.1038/s44319-023-00030-4)

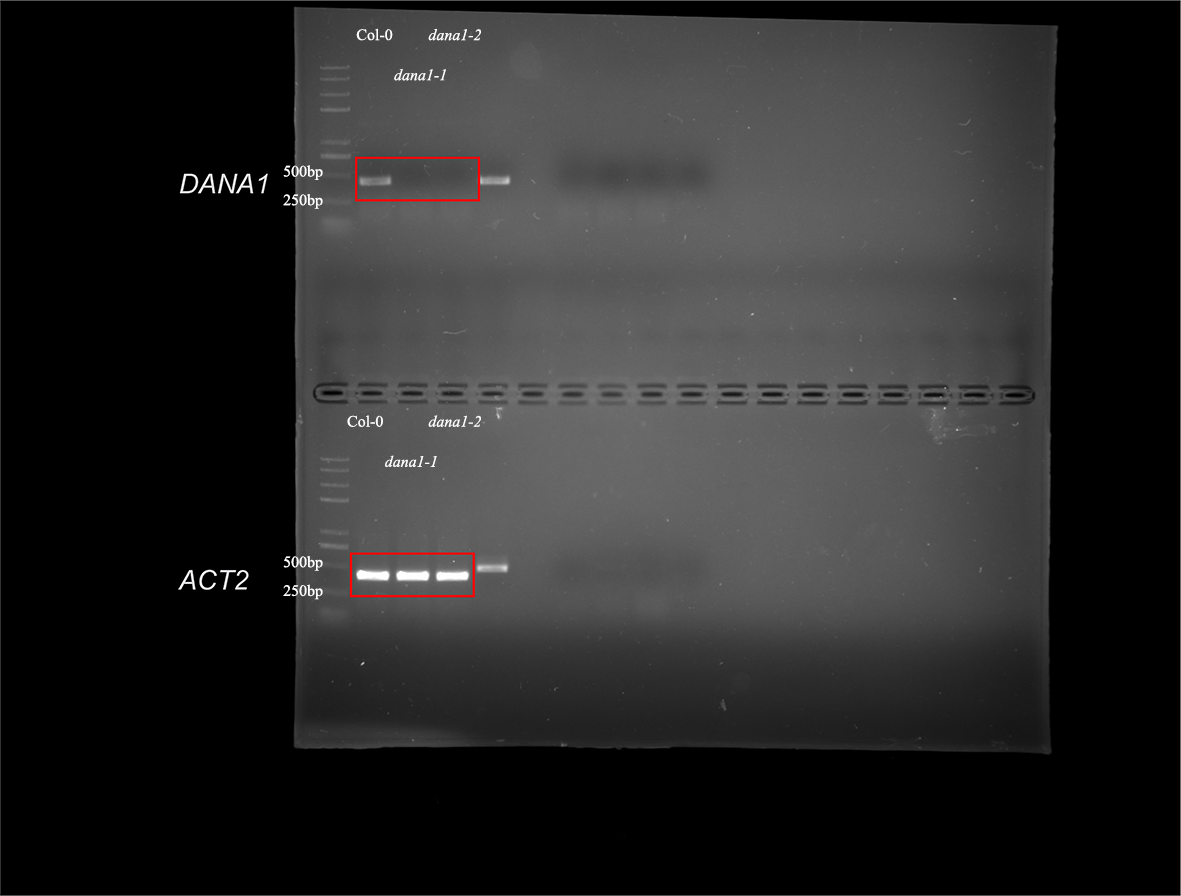

Supplement: Supplementary file 4 — Source Data Fig. 1 [file 44319_2023_30_MOESM4_ESM.zip › Fig. 1B/Fig.1B.tif]

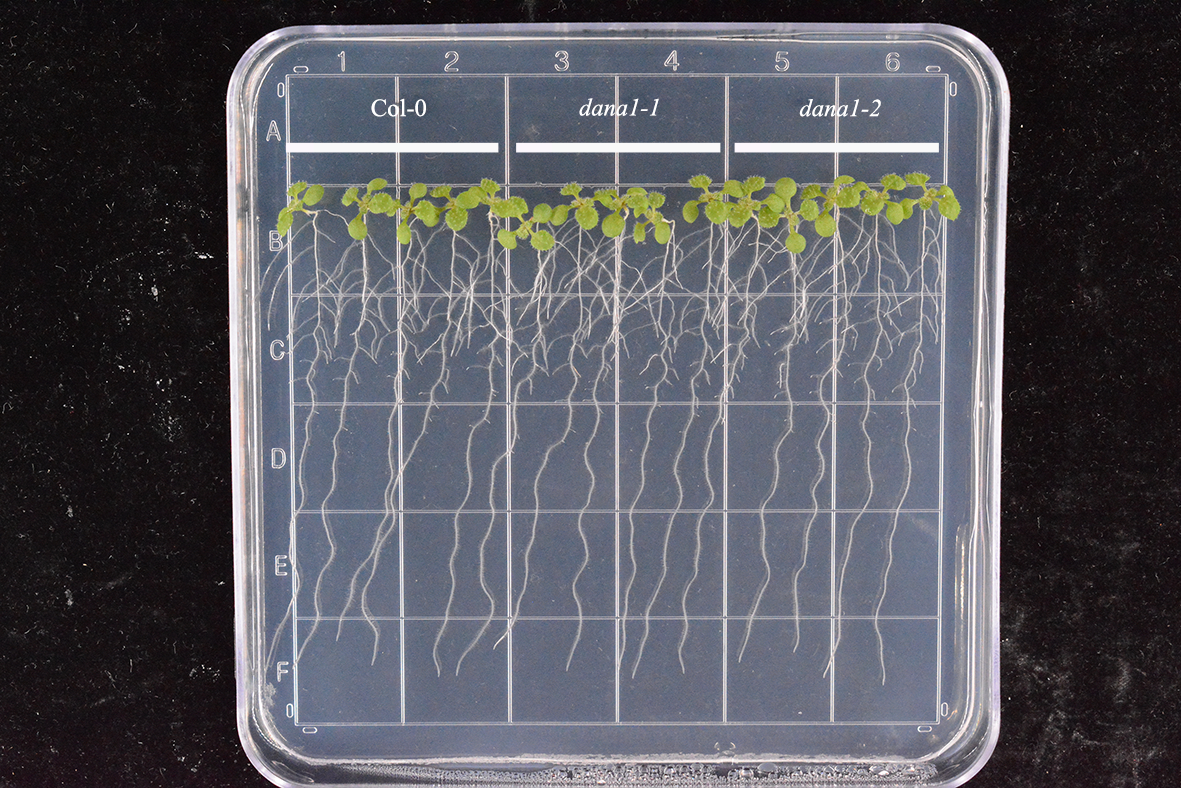

Supplement: Supplementary file 4 — Source Data Fig. 1 [file 44319_2023_30_MOESM4_ESM.zip › Fig. 1C/1-2 MS.tif]

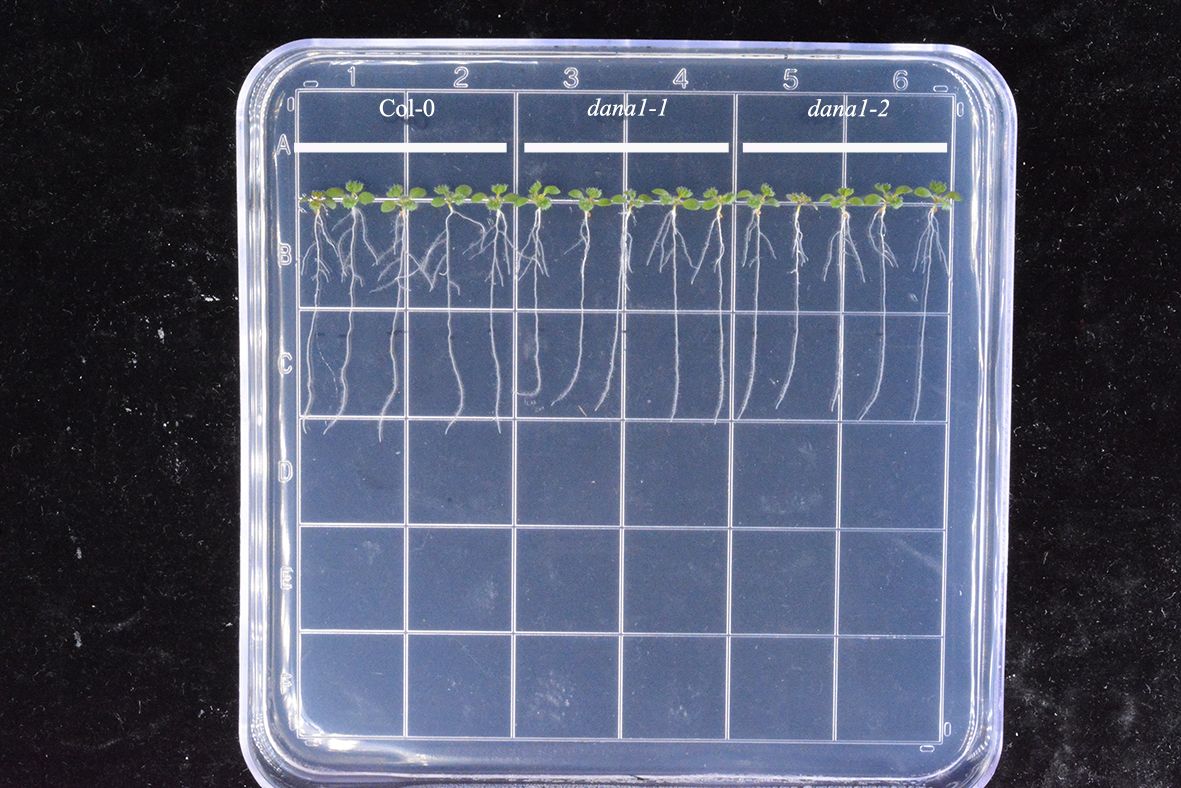

Supplement: Supplementary file 4 — Source Data Fig. 1 [file 44319_2023_30_MOESM4_ESM.zip › Fig. 1C/20% PEG.tif]

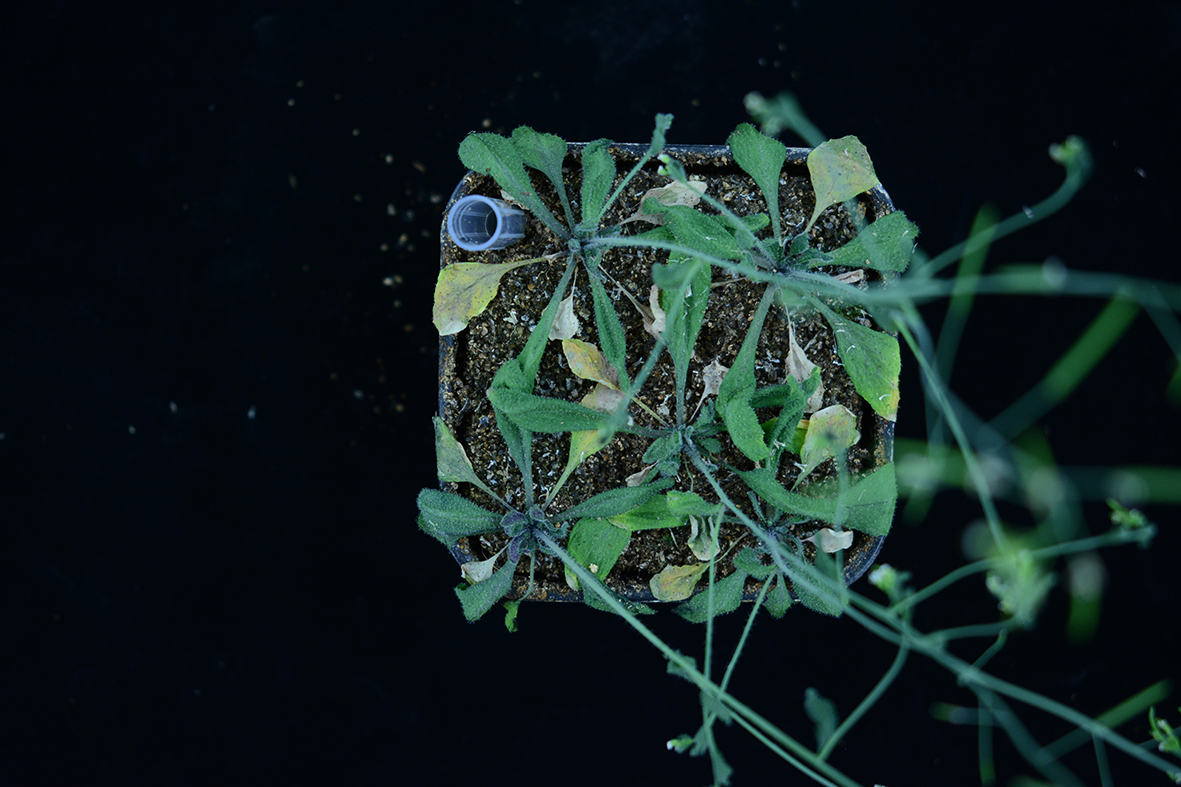

Supplement: Supplementary file 4 — Source Data Fig. 1 [file 44319_2023_30_MOESM4_ESM.zip › Fig. 1F/Col-0 drought for twelve days.tif]

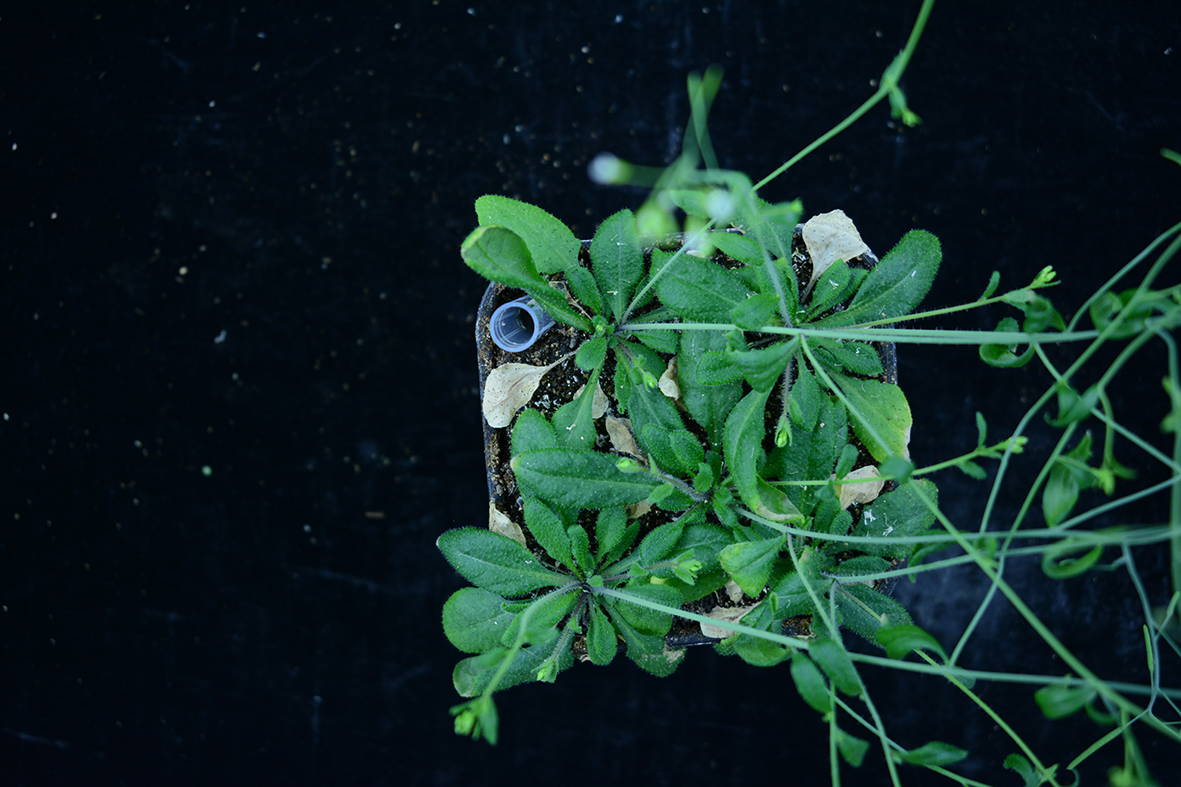

Supplement: Supplementary file 4 — Source Data Fig. 1 [file 44319_2023_30_MOESM4_ESM.zip › Fig. 1F/Col-0 five days after rewatering.tif]

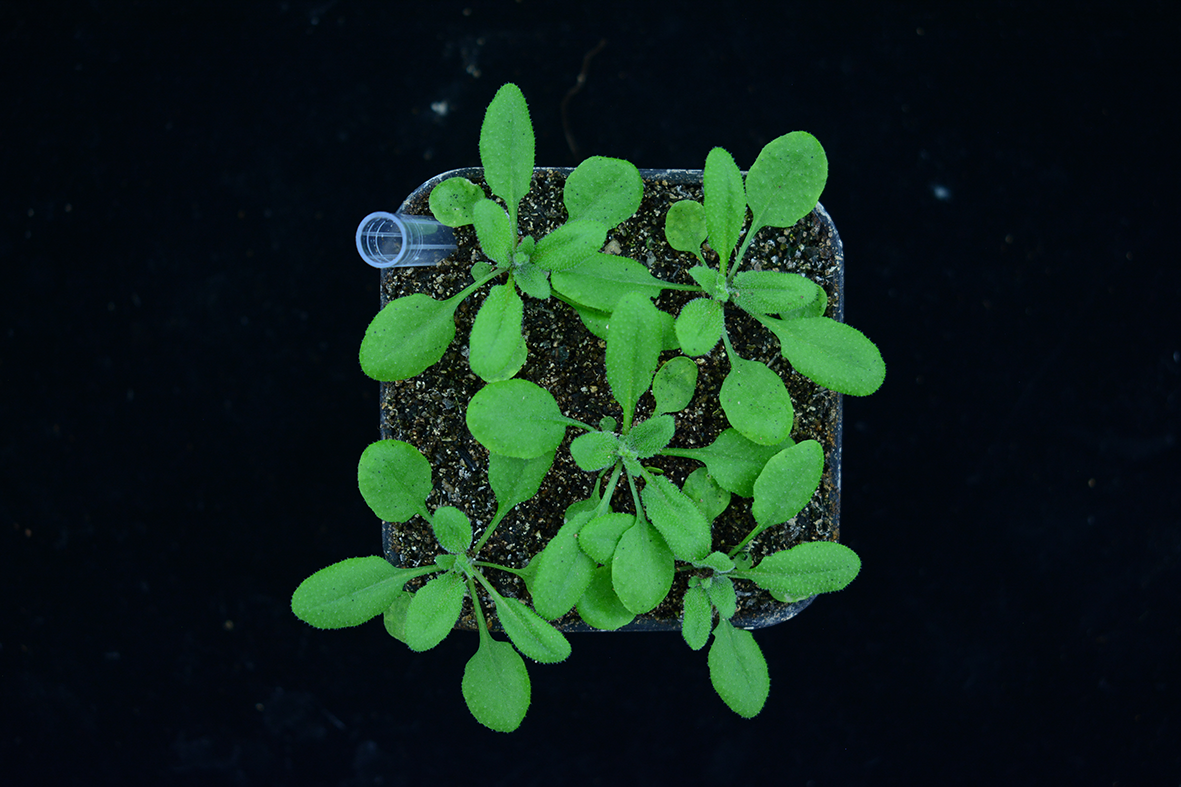

Supplement: Supplementary file 4 — Source Data Fig. 1 [file 44319_2023_30_MOESM4_ESM.zip › Fig. 1F/Col-0 three-week-old seedlings.tif]

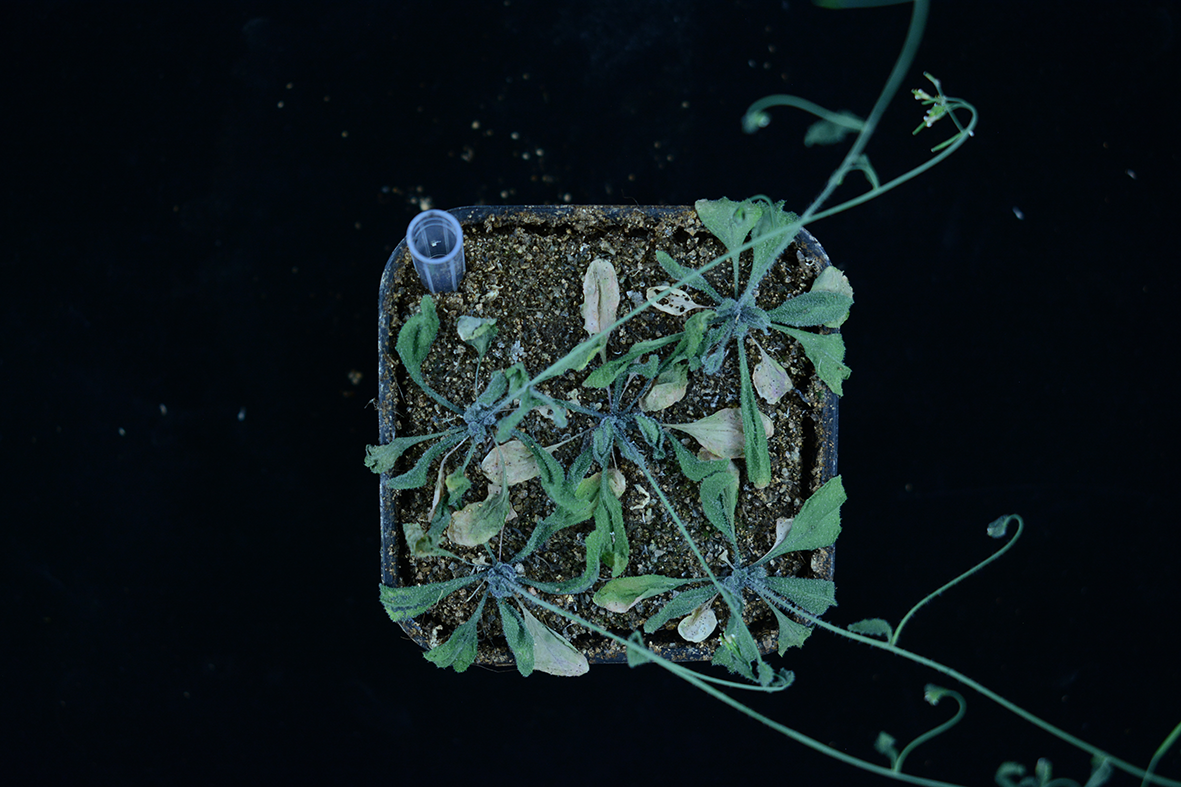

Supplement: Supplementary file 4 — Source Data Fig. 1 [file 44319_2023_30_MOESM4_ESM.zip › Fig. 1F/dana1-1 drought for twelve days.tif]

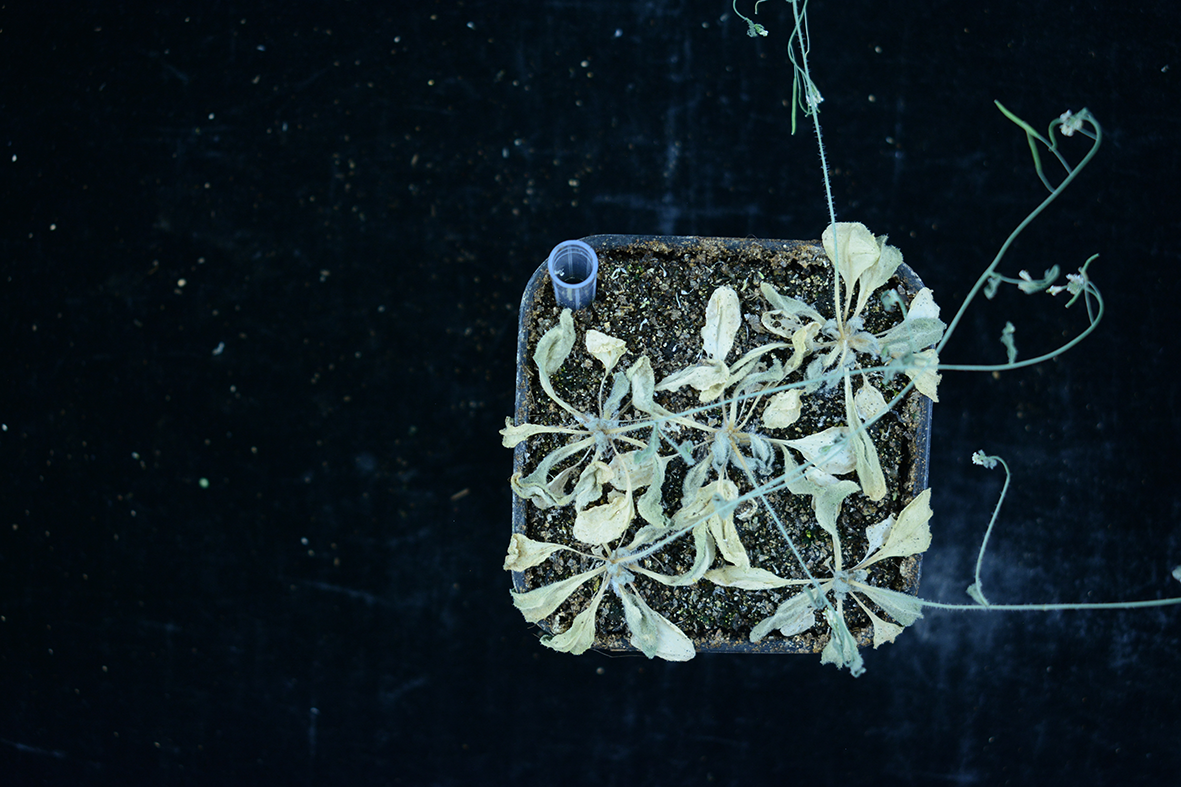

Supplement: Supplementary file 4 — Source Data Fig. 1 [file 44319_2023_30_MOESM4_ESM.zip › Fig. 1F/dana1-1 five days after rewatering.tif]

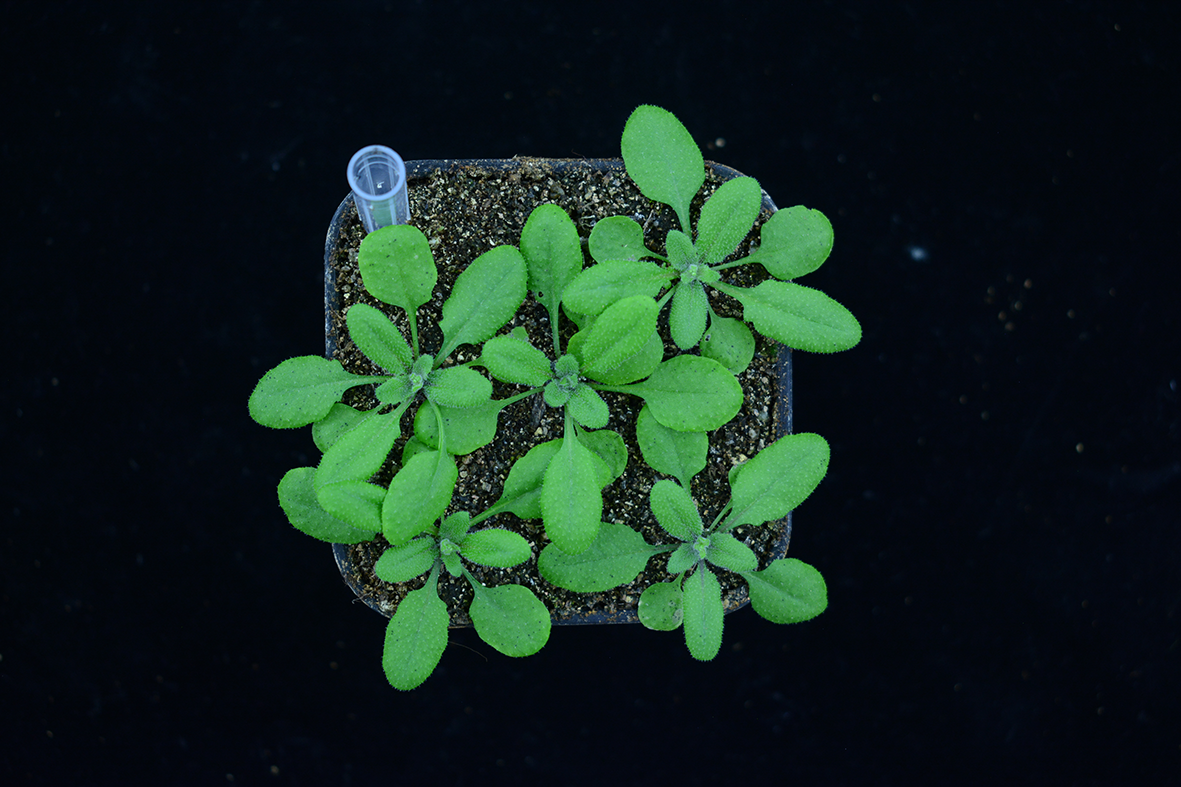

Supplement: Supplementary file 4 — Source Data Fig. 1 [file 44319_2023_30_MOESM4_ESM.zip › Fig. 1F/dana1-1 three-week-old seedlings.tif]

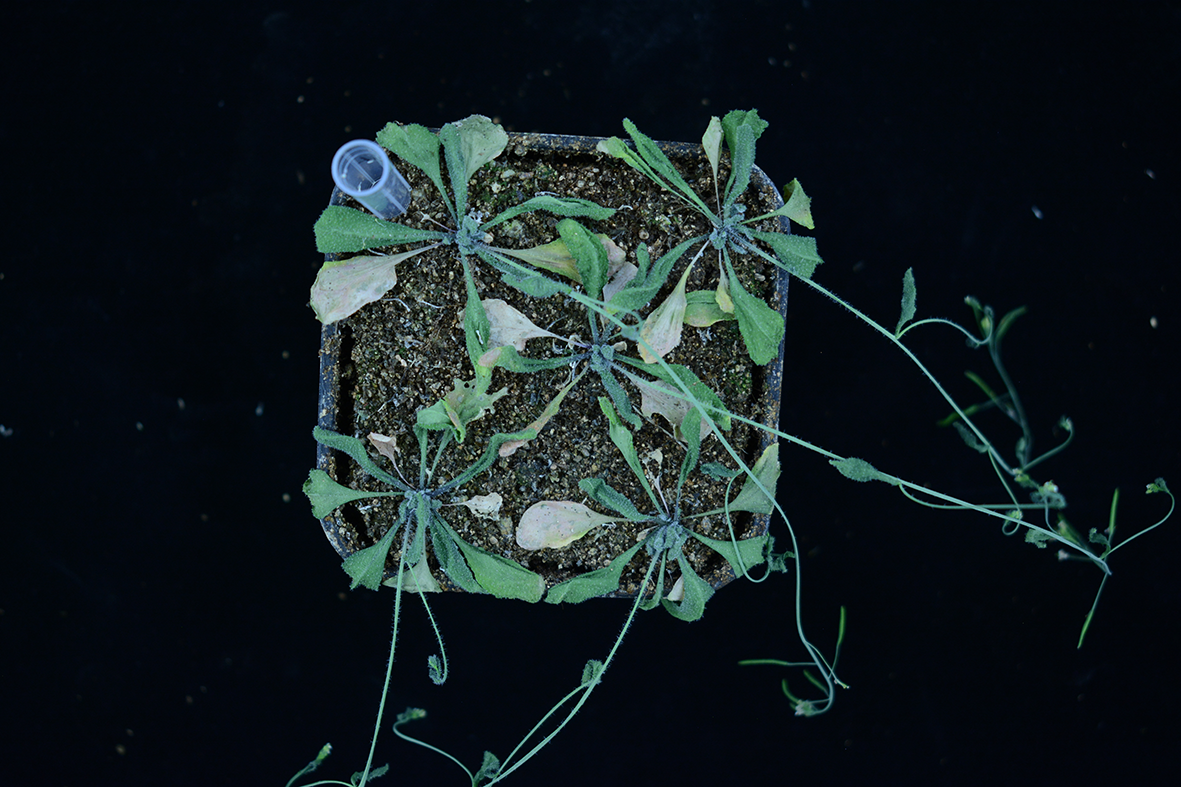

Supplement: Supplementary file 4 — Source Data Fig. 1 [file 44319_2023_30_MOESM4_ESM.zip › Fig. 1F/dana1-2 drought for twelve days.tif]

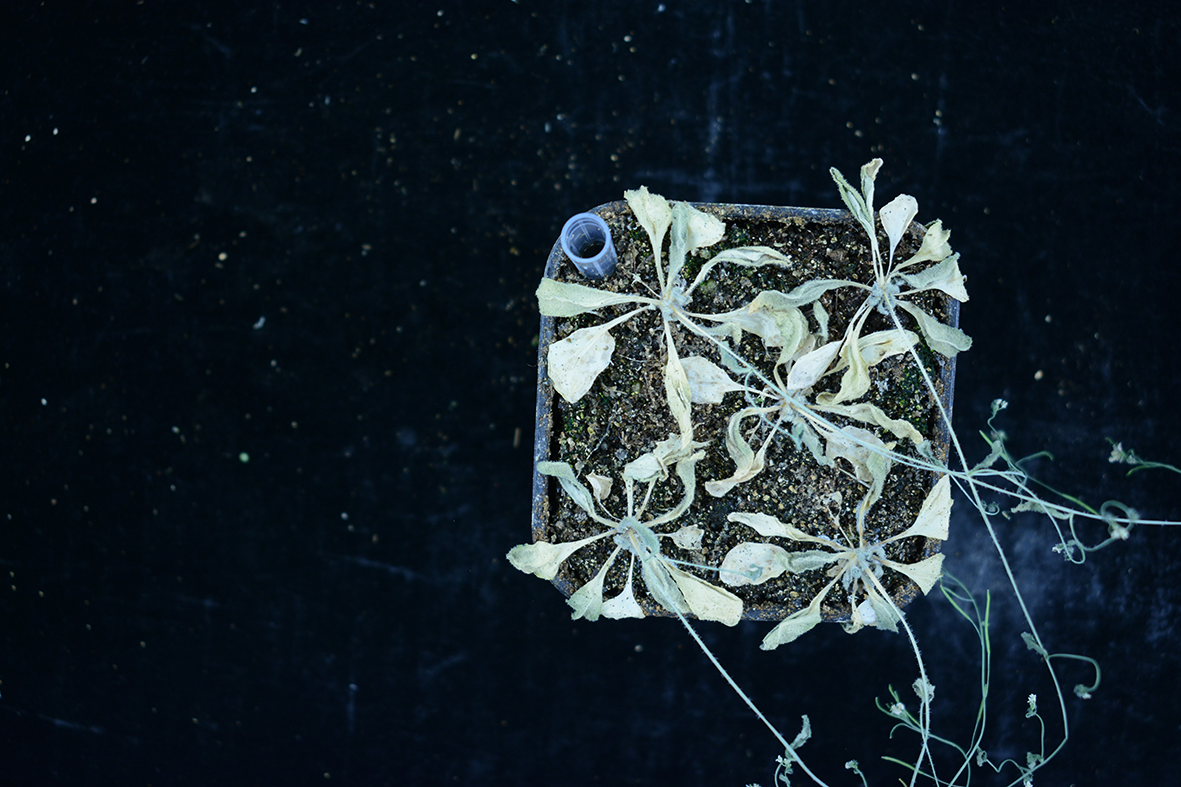

Supplement: Supplementary file 4 — Source Data Fig. 1 [file 44319_2023_30_MOESM4_ESM.zip › Fig. 1F/dana1-2 five days after rewatering.tif]

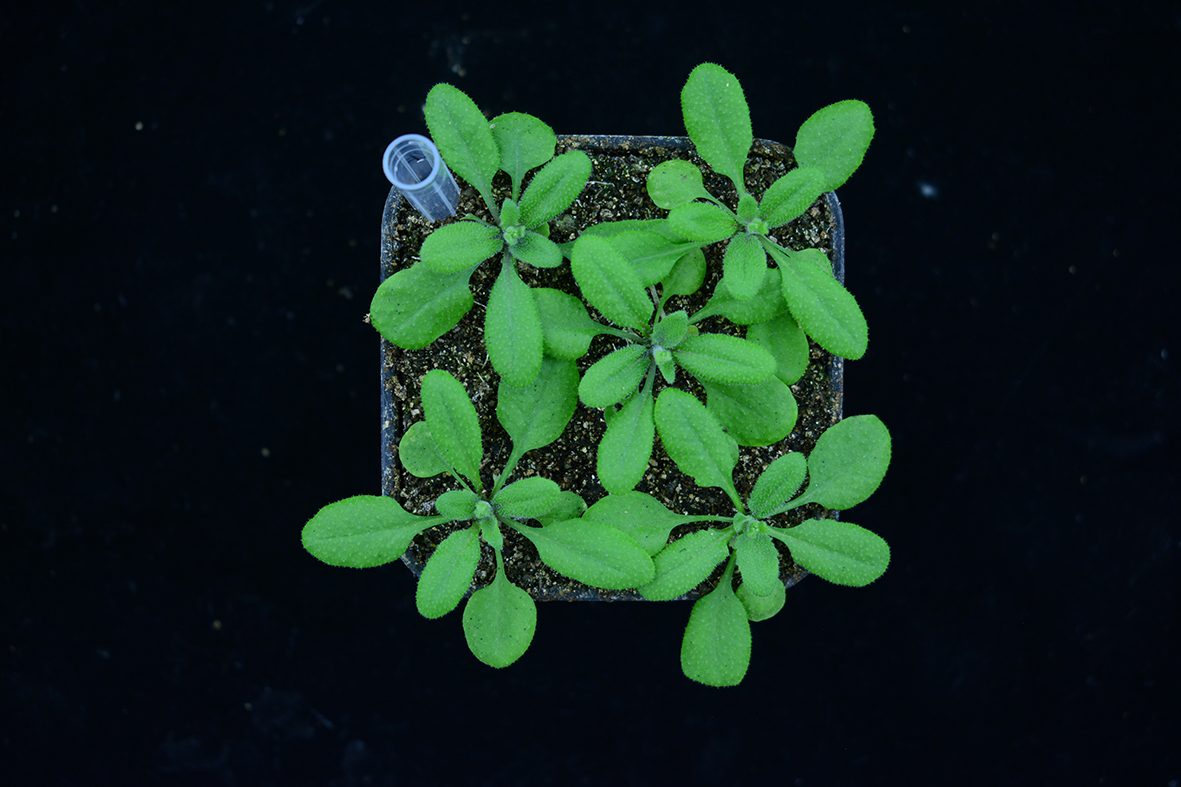

Supplement: Supplementary file 4 — Source Data Fig. 1 [file 44319_2023_30_MOESM4_ESM.zip › Fig. 1F/dana1-2 three-week-old seedlings.tif]

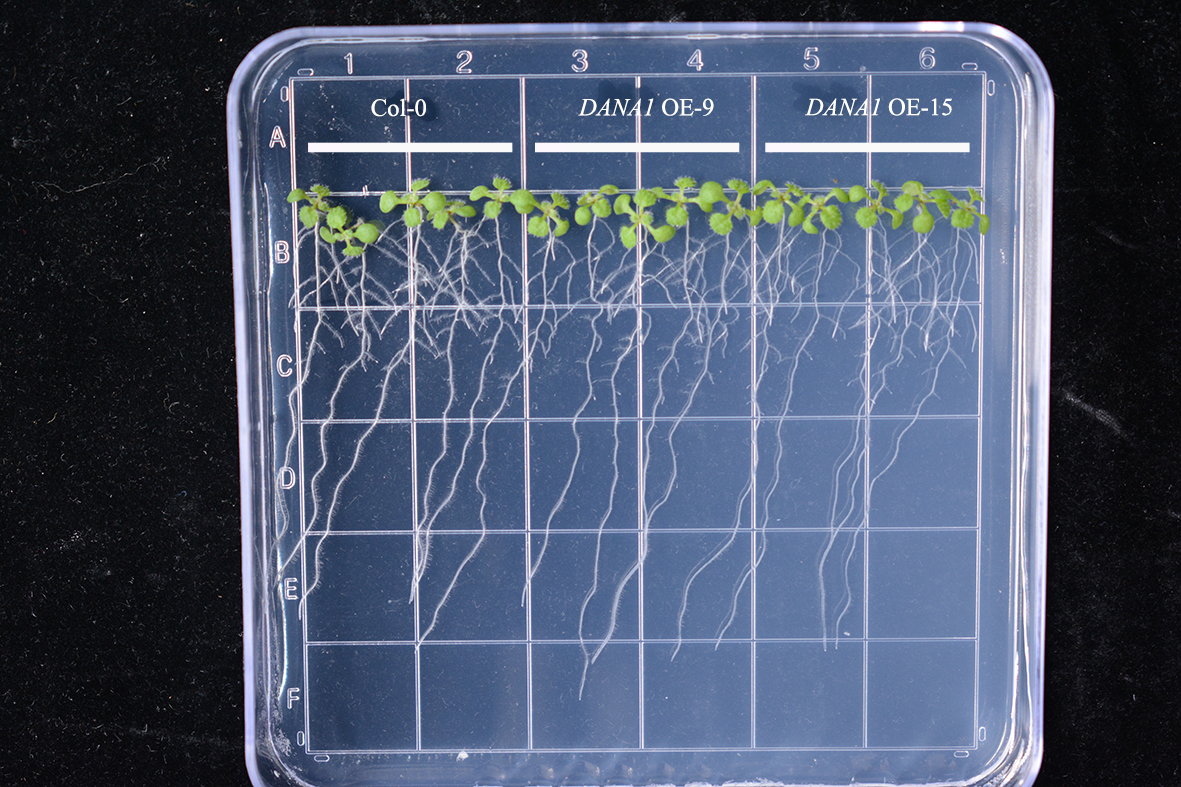

Supplement: Supplementary file 5 — Source Data Fig. 2 [file 44319_2023_30_MOESM5_ESM.zip › Fig.2B/1-2 MS.tif]

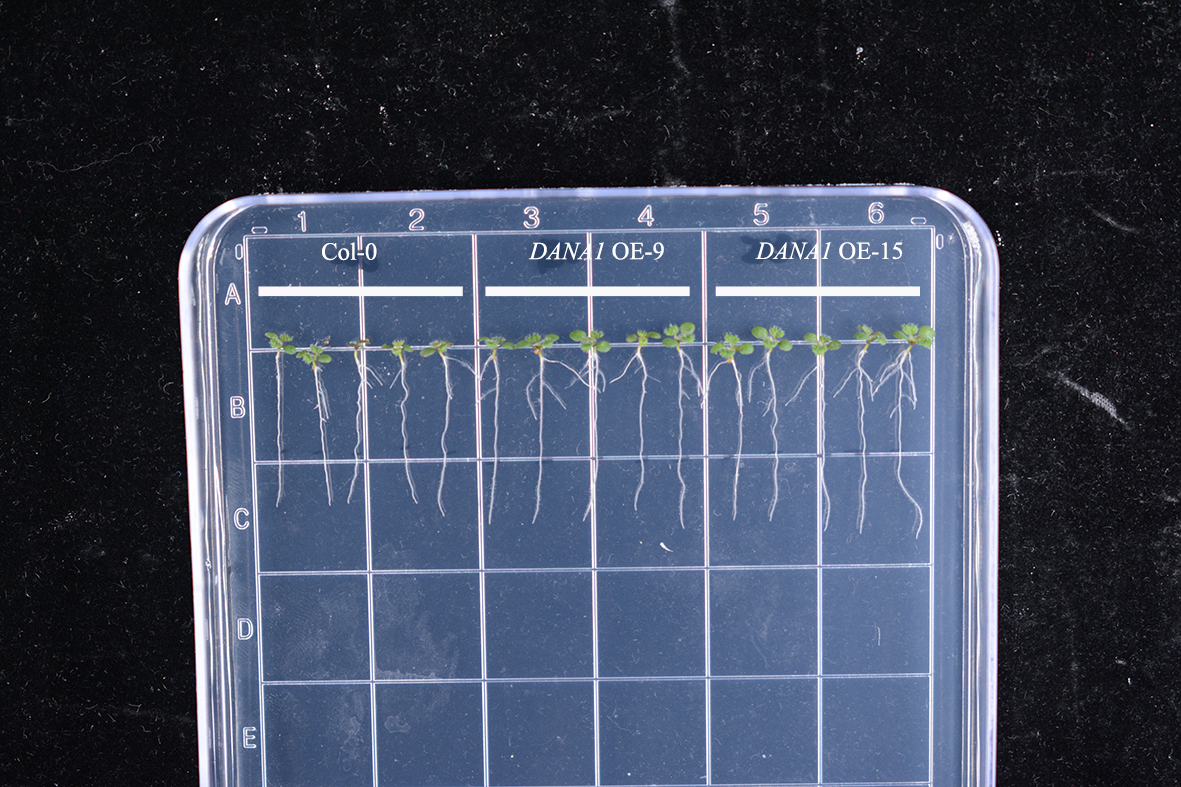

Supplement: Supplementary file 5 — Source Data Fig. 2 [file 44319_2023_30_MOESM5_ESM.zip › Fig.2B/20% PEG.tif]

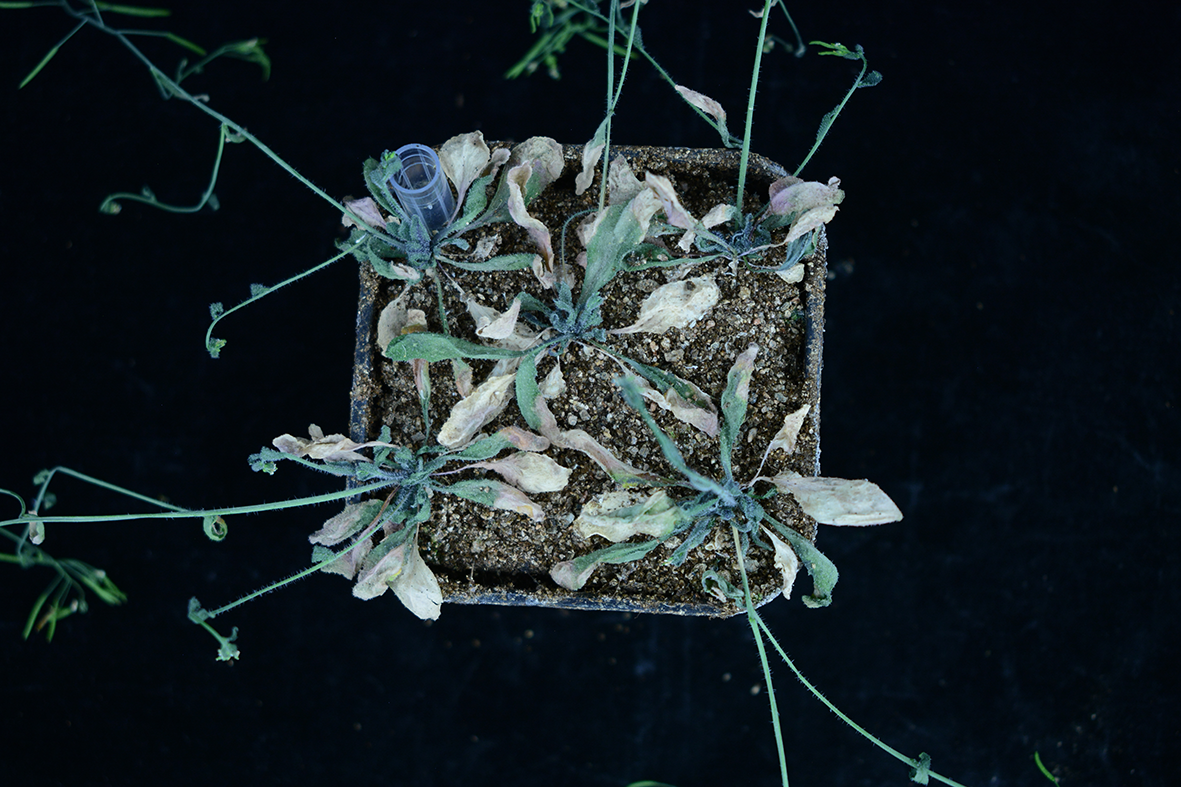

Supplement: Supplementary file 5 — Source Data Fig. 2 [file 44319_2023_30_MOESM5_ESM.zip › Fig.2D/Col-0 drought for eighteen days.tif]

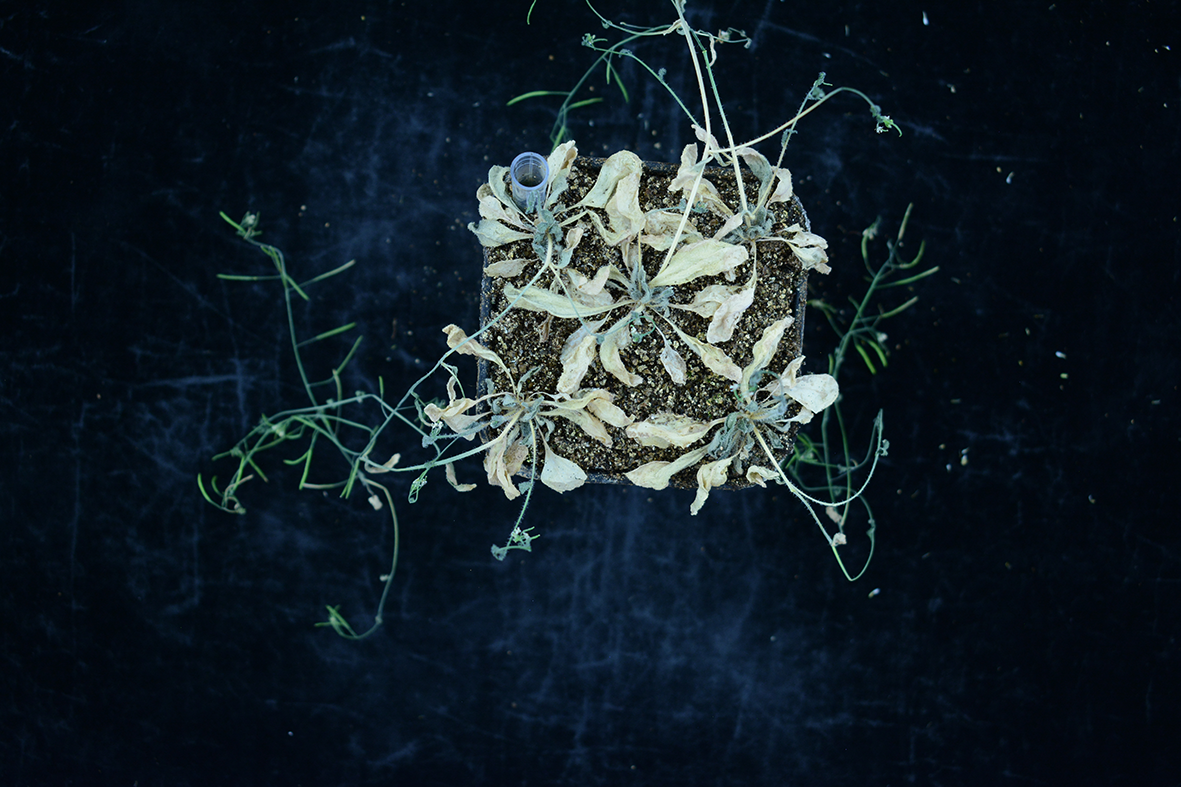

Supplement: Supplementary file 5 — Source Data Fig. 2 [file 44319_2023_30_MOESM5_ESM.zip › Fig.2D/Col-0 five days after rewatering.tif]

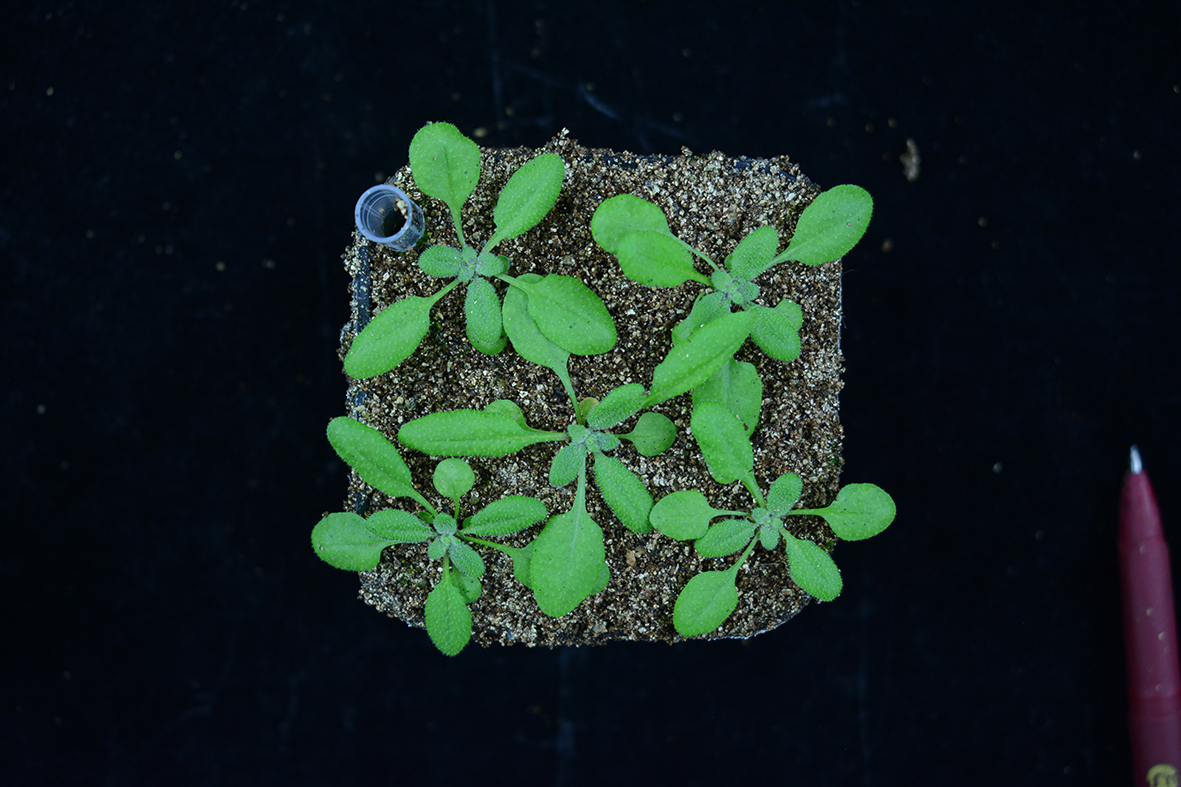

Supplement: Supplementary file 5 — Source Data Fig. 2 [file 44319_2023_30_MOESM5_ESM.zip › Fig.2D/Col-0 three-week-old seedlings.tif]

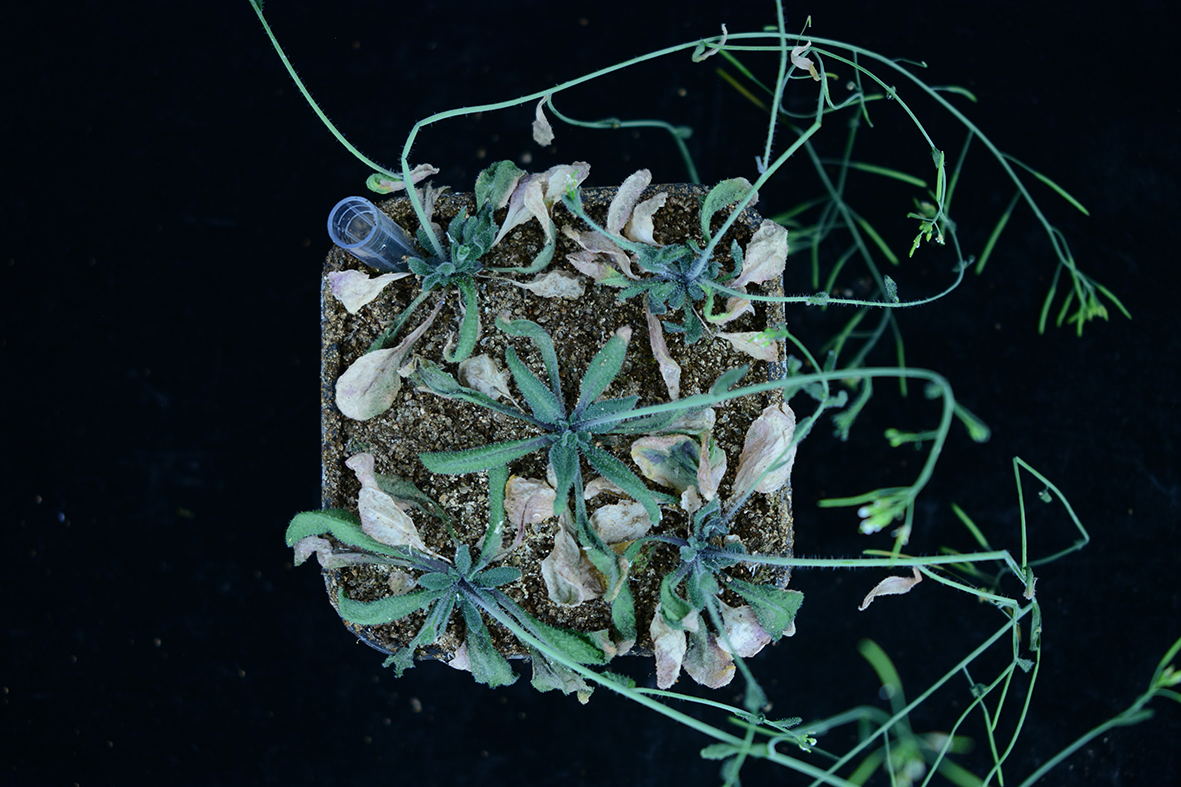

Supplement: Supplementary file 5 — Source Data Fig. 2 [file 44319_2023_30_MOESM5_ESM.zip › Fig.2D/DANA1 OE-15 drought for eighteen days.tif]

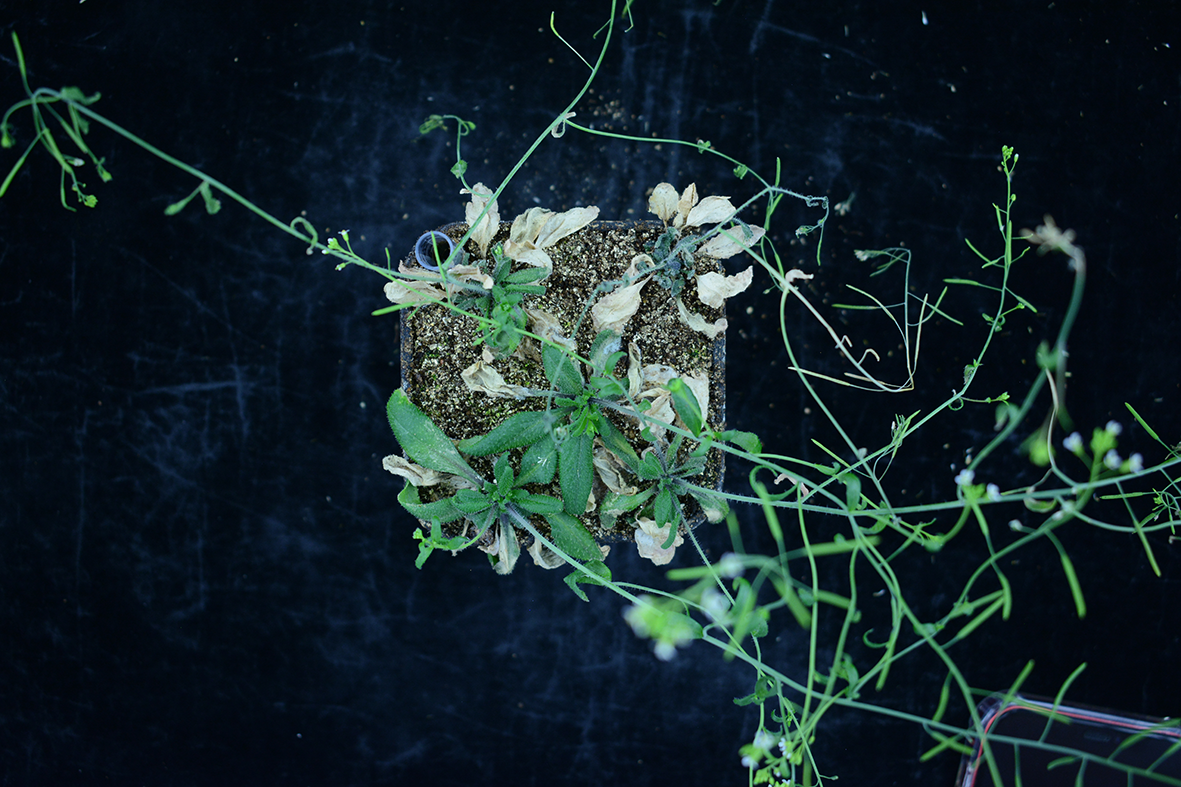

Supplement: Supplementary file 5 — Source Data Fig. 2 [file 44319_2023_30_MOESM5_ESM.zip › Fig.2D/DANA1 OE-15 five days after rewatering.tif]

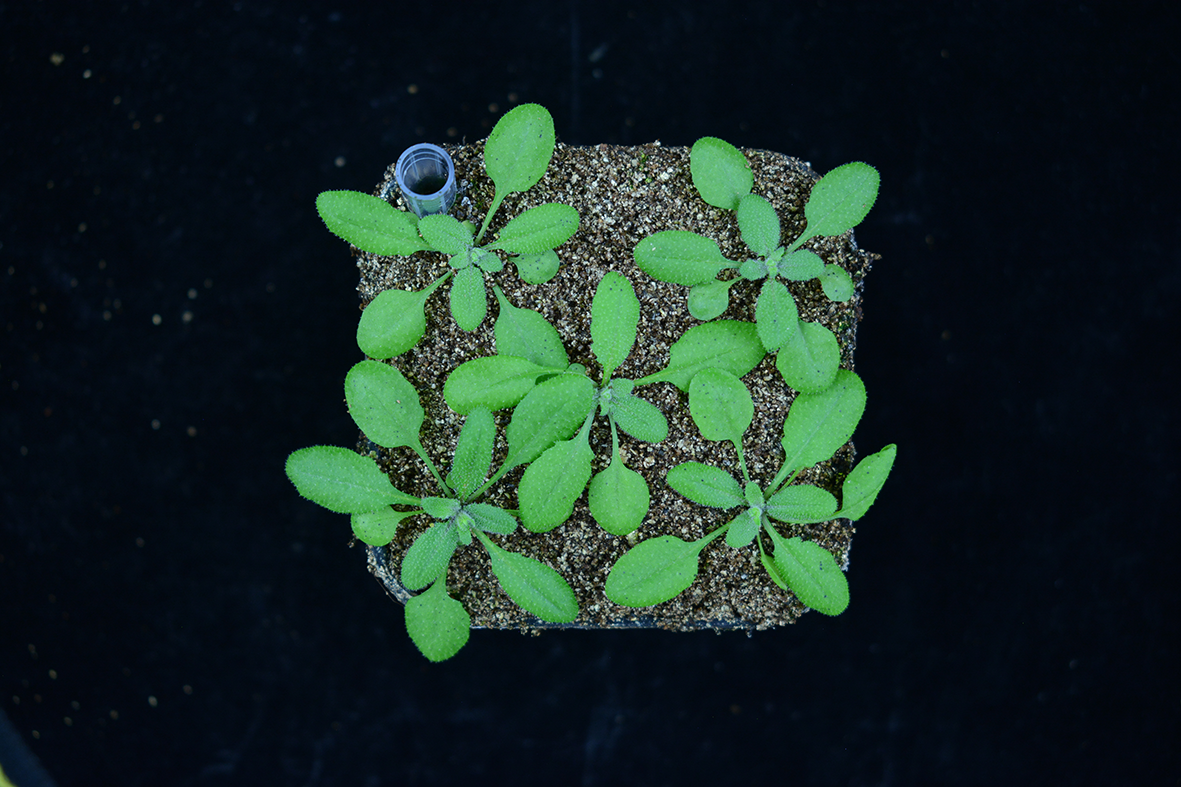

Supplement: Supplementary file 5 — Source Data Fig. 2 [file 44319_2023_30_MOESM5_ESM.zip › Fig.2D/DANA1 OE-15 three-week-old seedlings.tif]

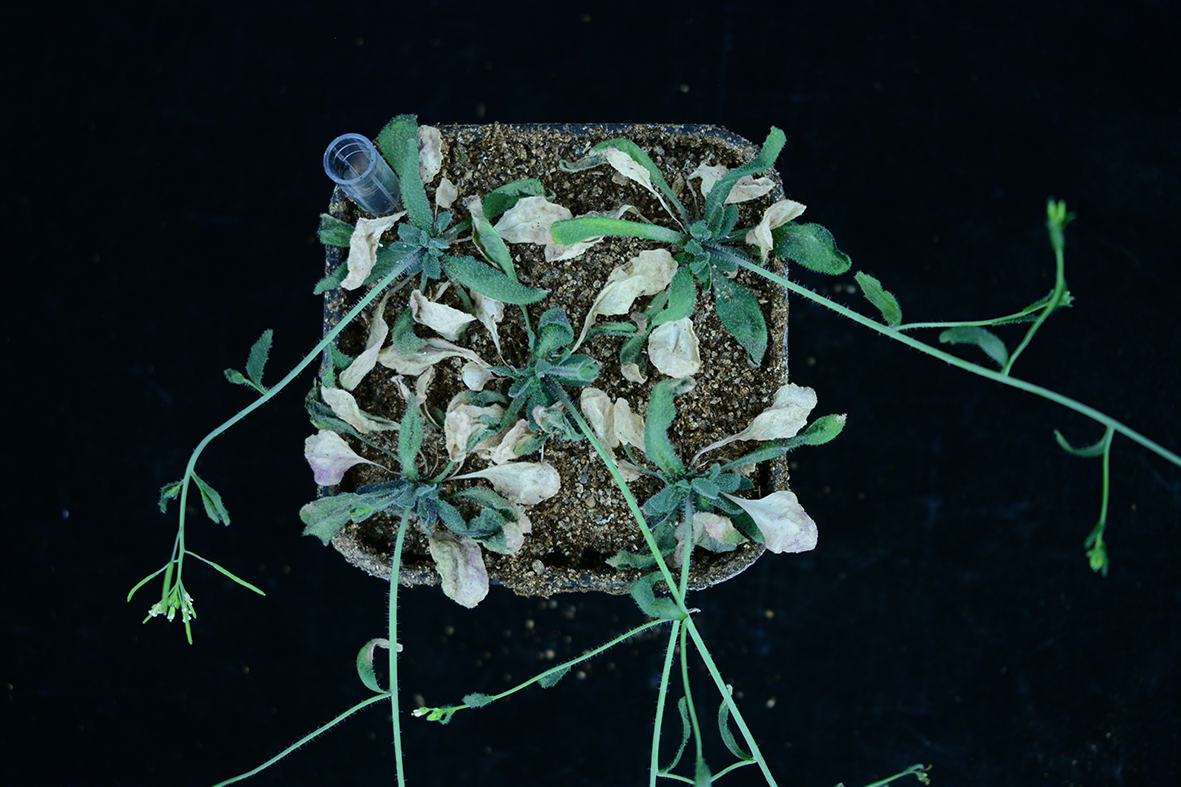

Supplement: Supplementary file 5 — Source Data Fig. 2 [file 44319_2023_30_MOESM5_ESM.zip › Fig.2D/DANA1 OE-9 drought for eighteen days.tif]

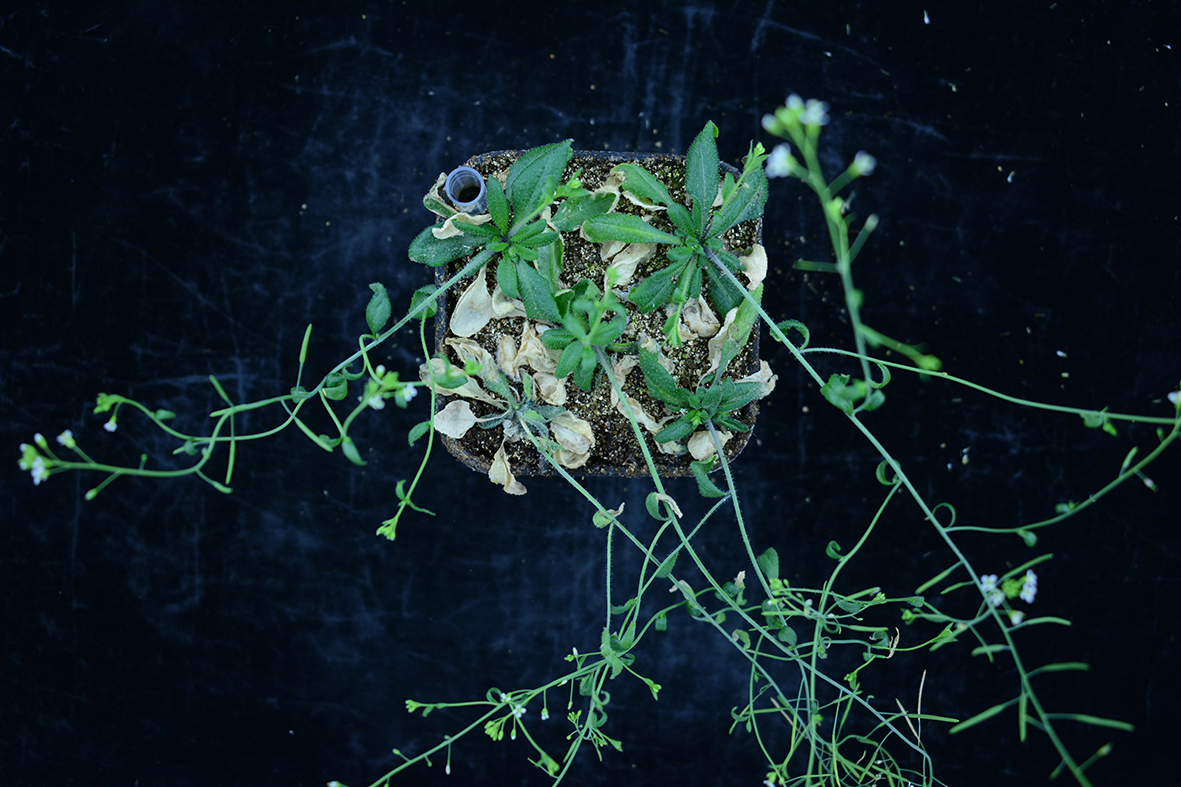

Supplement: Supplementary file 5 — Source Data Fig. 2 [file 44319_2023_30_MOESM5_ESM.zip › Fig.2D/DANA1 OE-9 five days after rewatering.tif]

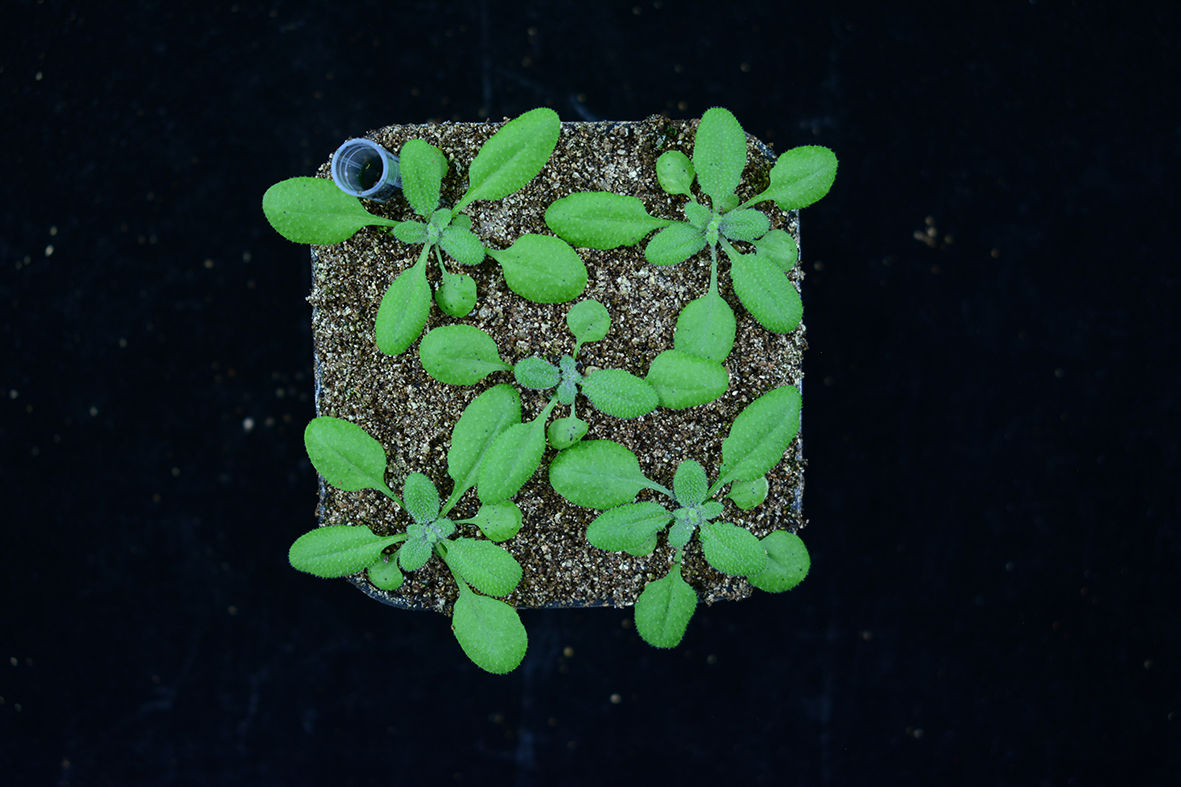

Supplement: Supplementary file 5 — Source Data Fig. 2 [file 44319_2023_30_MOESM5_ESM.zip › Fig.2D/DANA1 OE-9 three-week-old seedlings.tif]

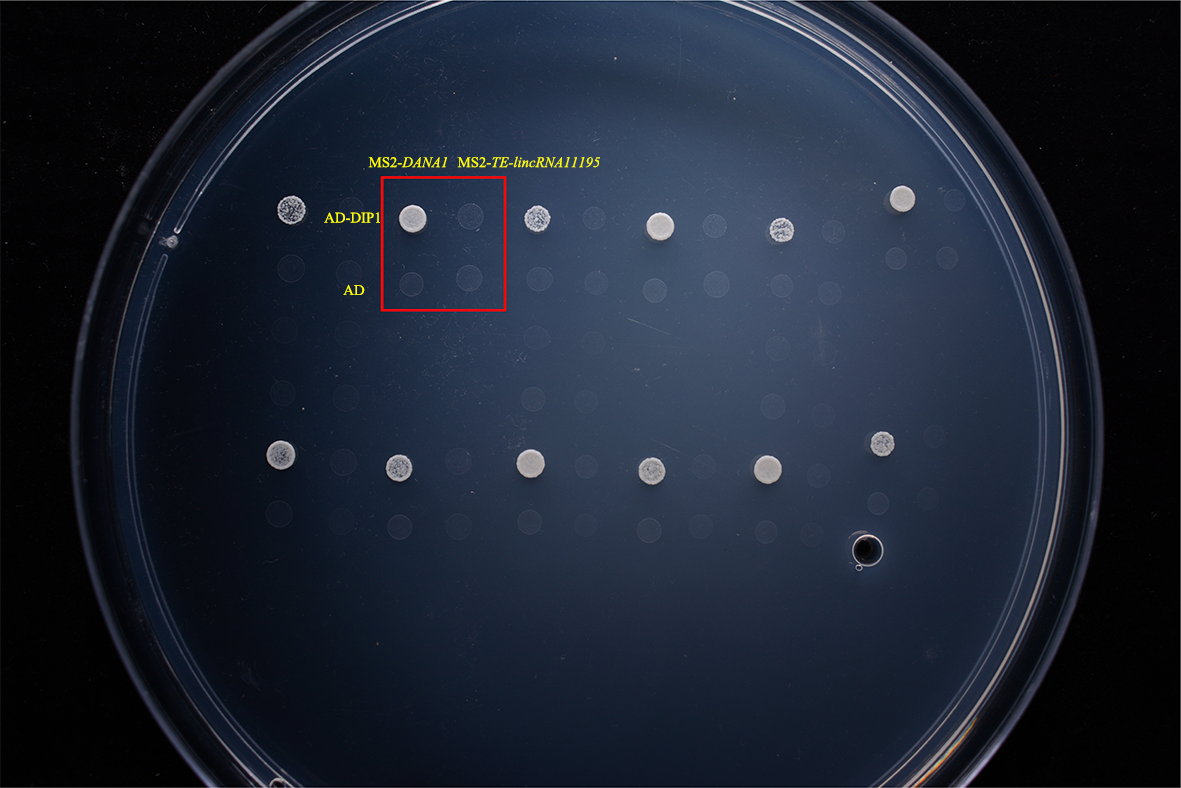

Supplement: Supplementary file 6 — Source Data Fig. 3 [file 44319_2023_30_MOESM6_ESM.zip › Fig.3A/-LHU.tif]

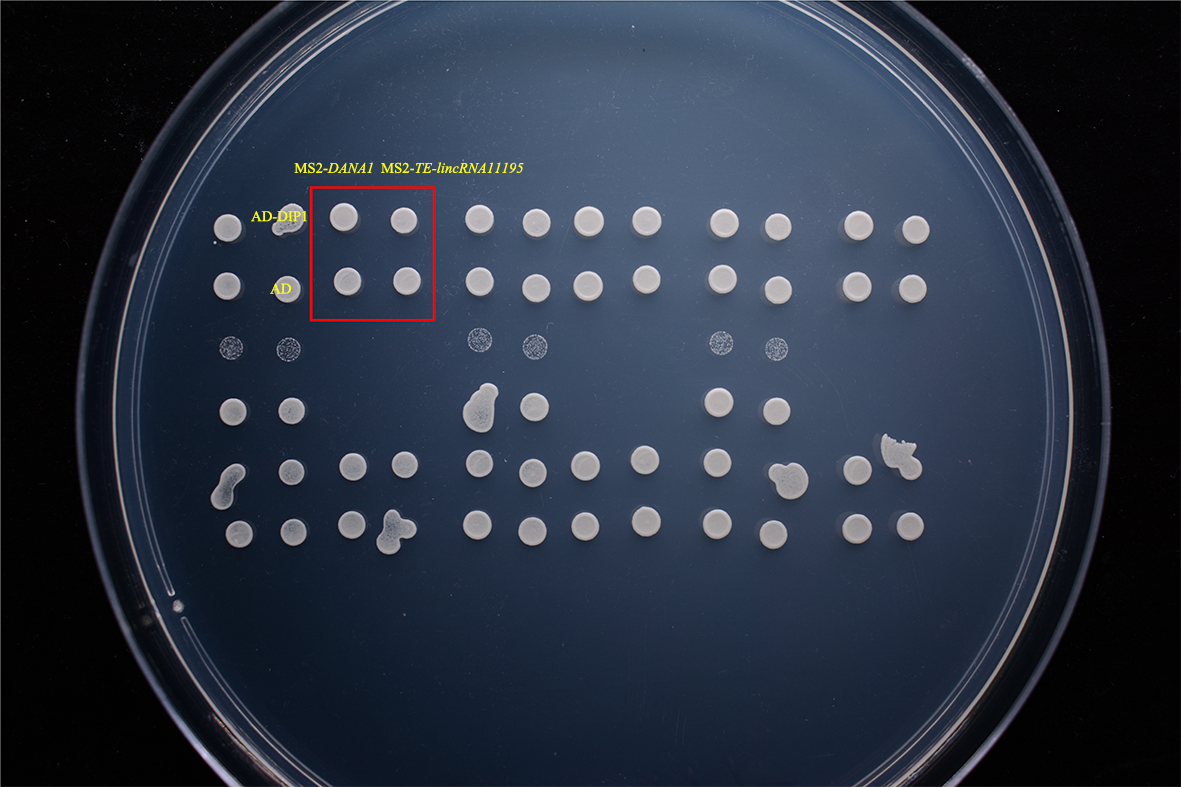

Supplement: Supplementary file 6 — Source Data Fig. 3 [file 44319_2023_30_MOESM6_ESM.zip › Fig.3A/-LU.tif]

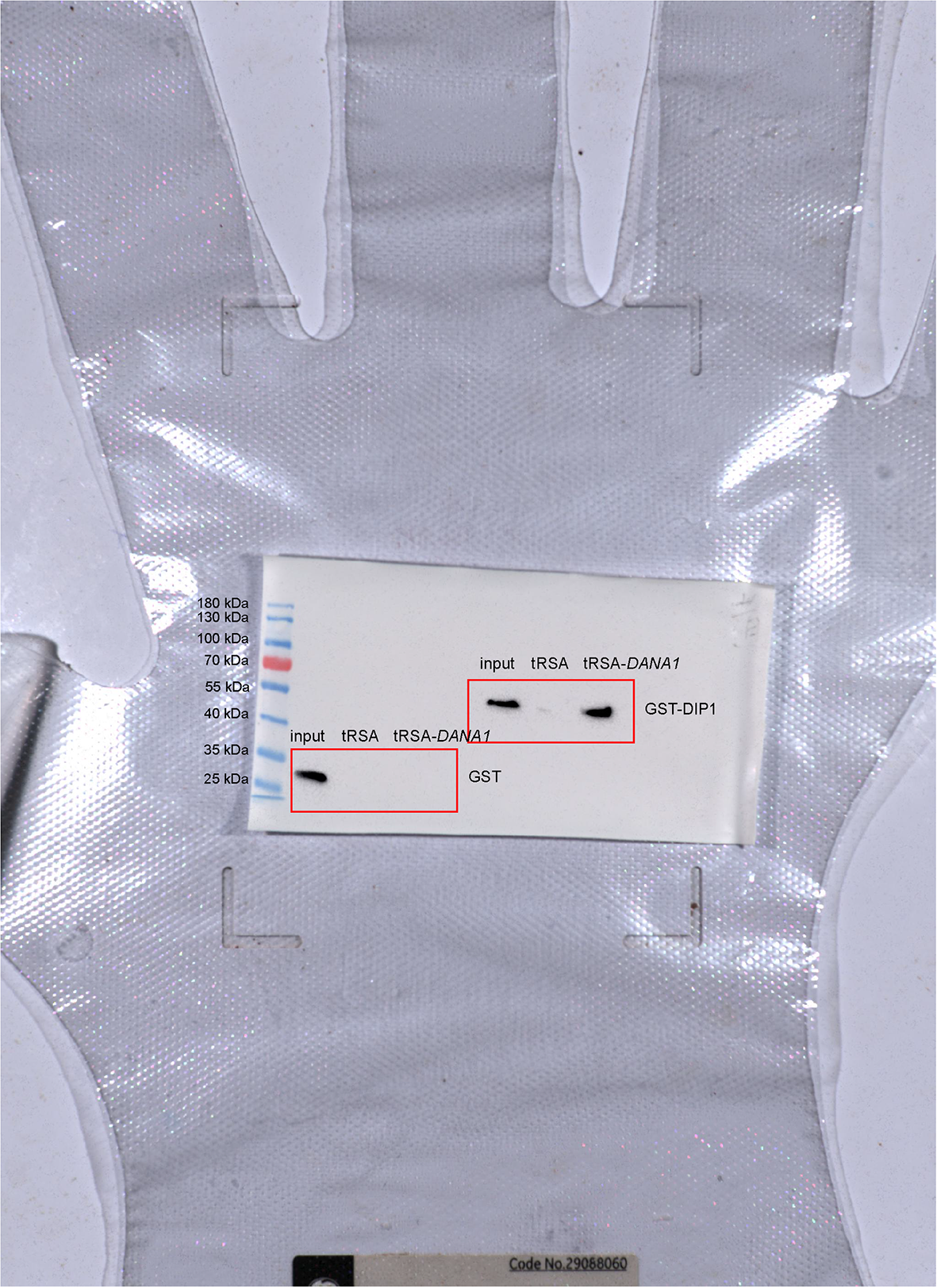

Supplement: Supplementary file 6 — Source Data Fig. 3 [file 44319_2023_30_MOESM6_ESM.zip › Fig.3B/Fig.3B.tif]

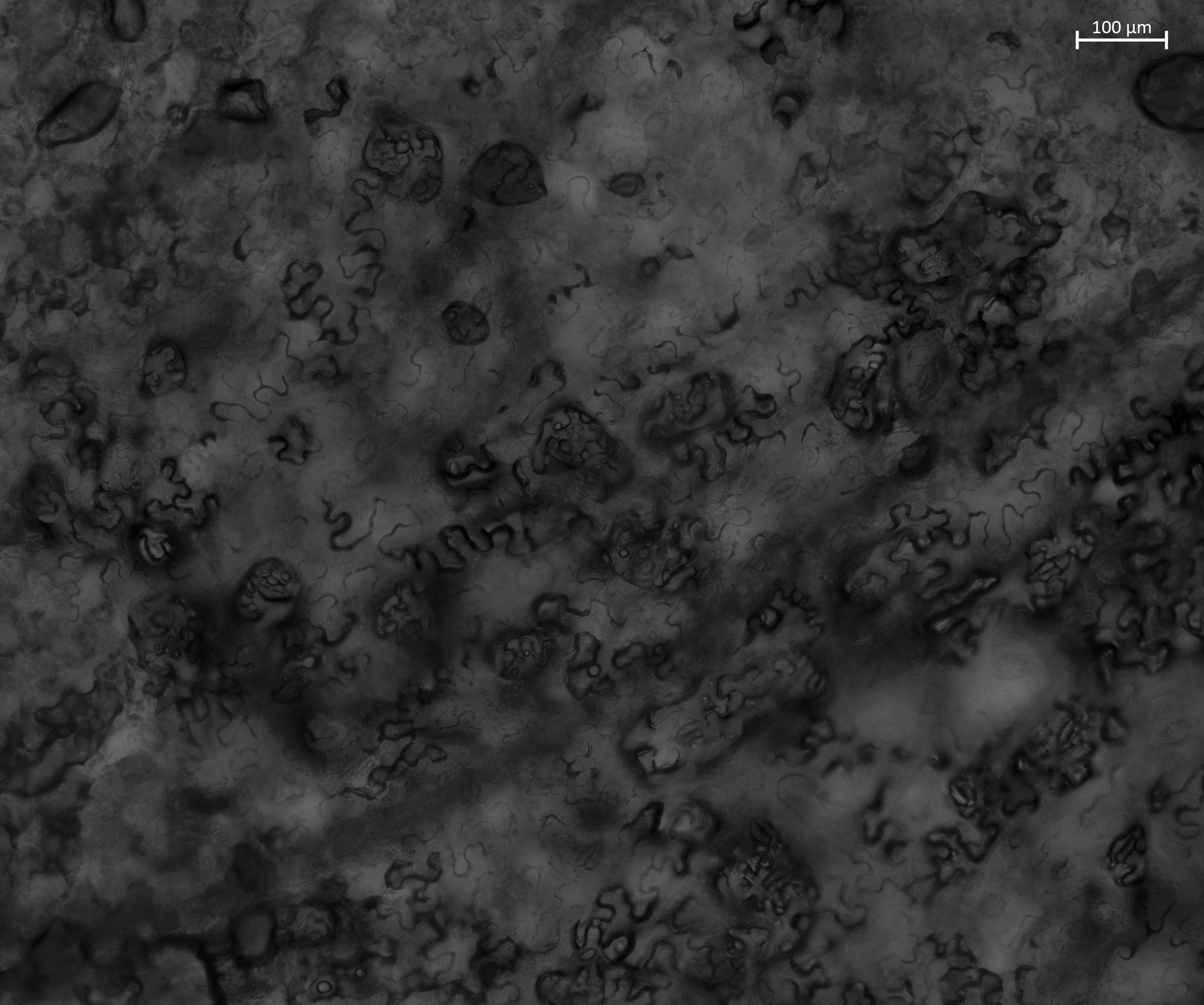

Supplement: Supplementary file 6 — Source Data Fig. 3 [file 44319_2023_30_MOESM6_ESM.zip › Fig.3C/BF (6xMS2 DIP1-nYFP cYFP-MSCP).tif]

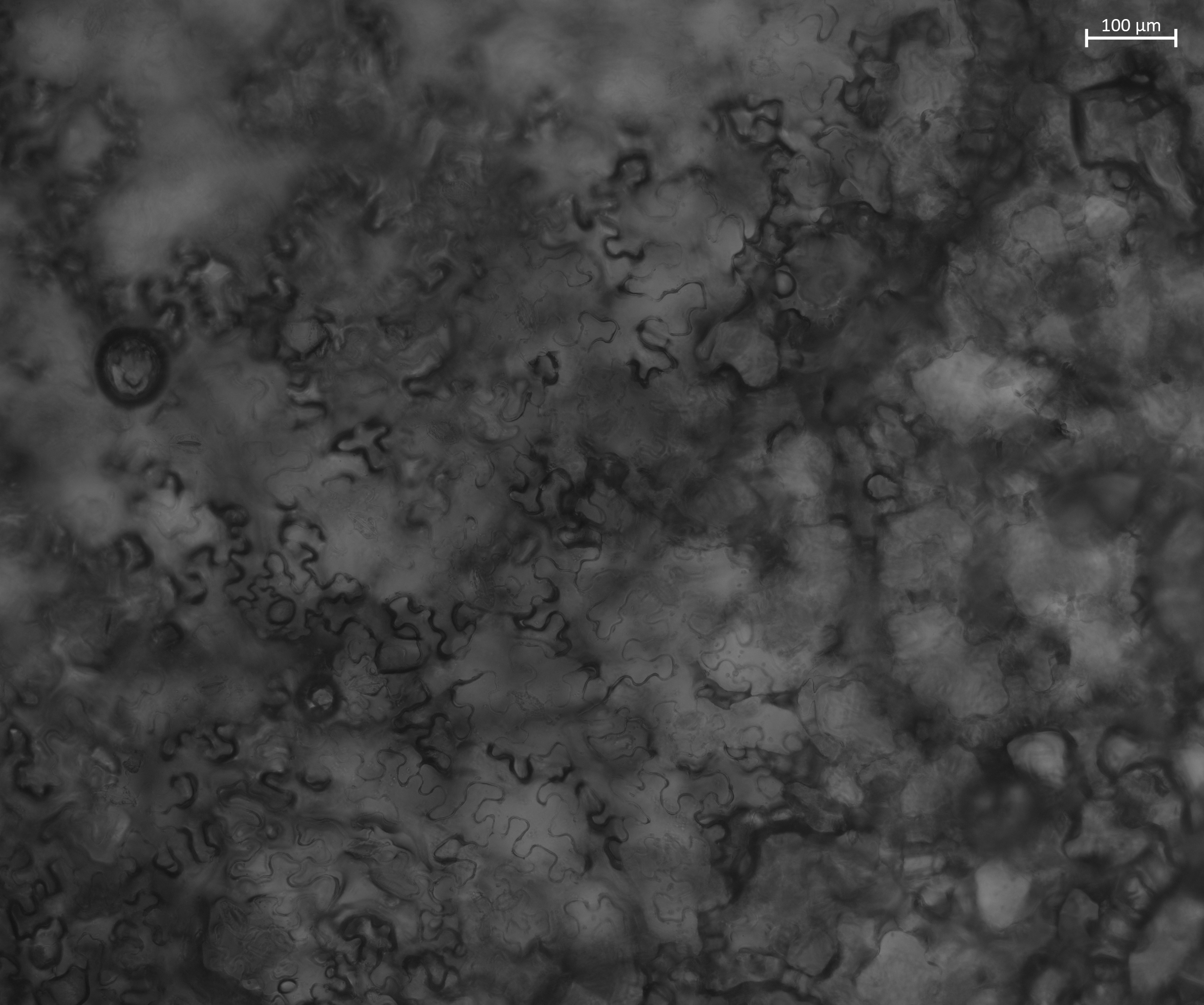

Supplement: Supplementary file 6 — Source Data Fig. 3 [file 44319_2023_30_MOESM6_ESM.zip › Fig.3C/BF (6xMS2-DANA1 DIP1-nYFP cYFP-MSCP).tif]

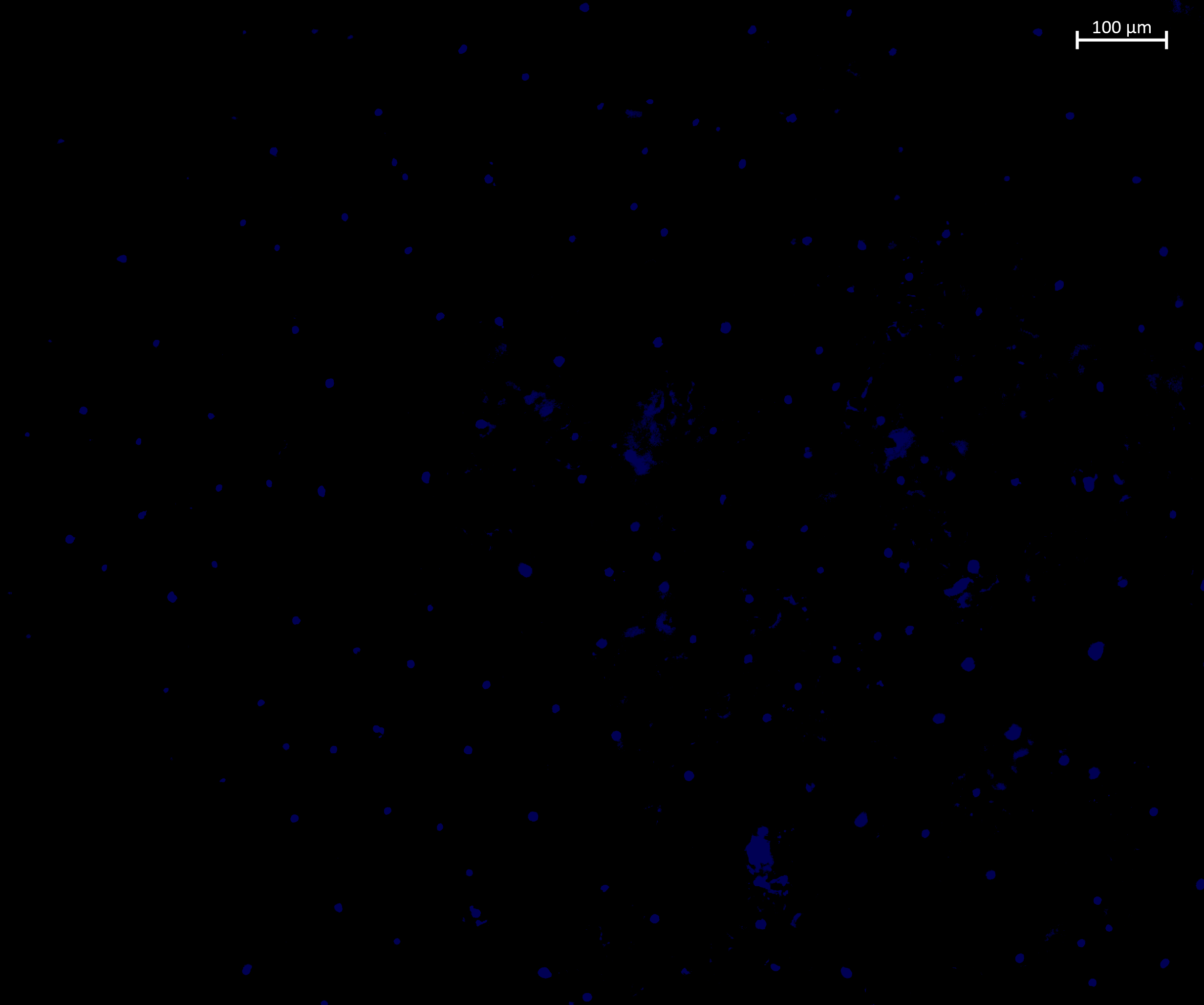

Supplement: Supplementary file 6 — Source Data Fig. 3 [file 44319_2023_30_MOESM6_ESM.zip › Fig.3C/DAPI (6xMS2 DIP1-nYFP cYFP-MSCP).tif]

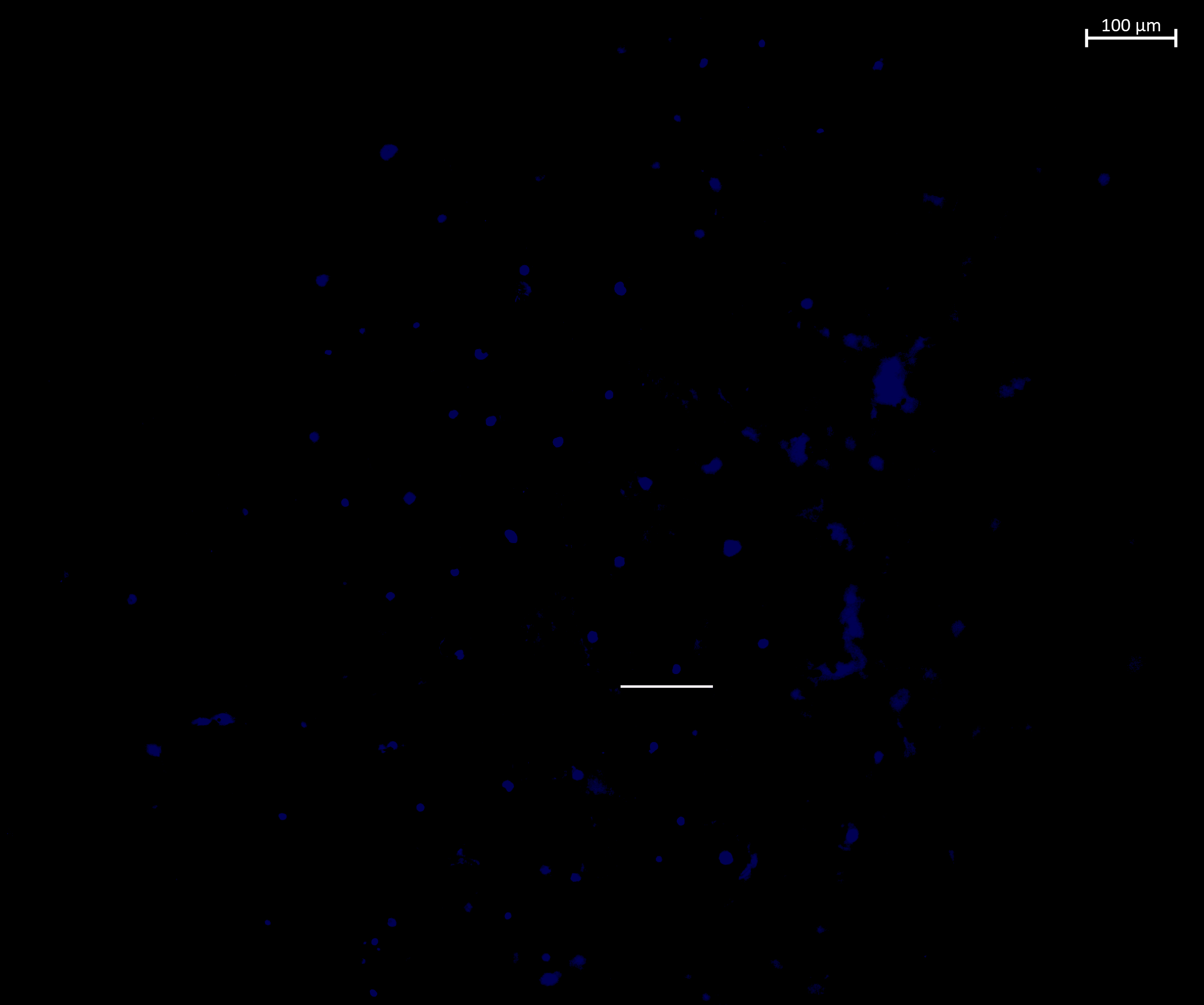

Supplement: Supplementary file 6 — Source Data Fig. 3 [file 44319_2023_30_MOESM6_ESM.zip › Fig.3C/DAPI (6xMS2-DANA1 DIP1-nYFP cYFP-MSCP).tif]

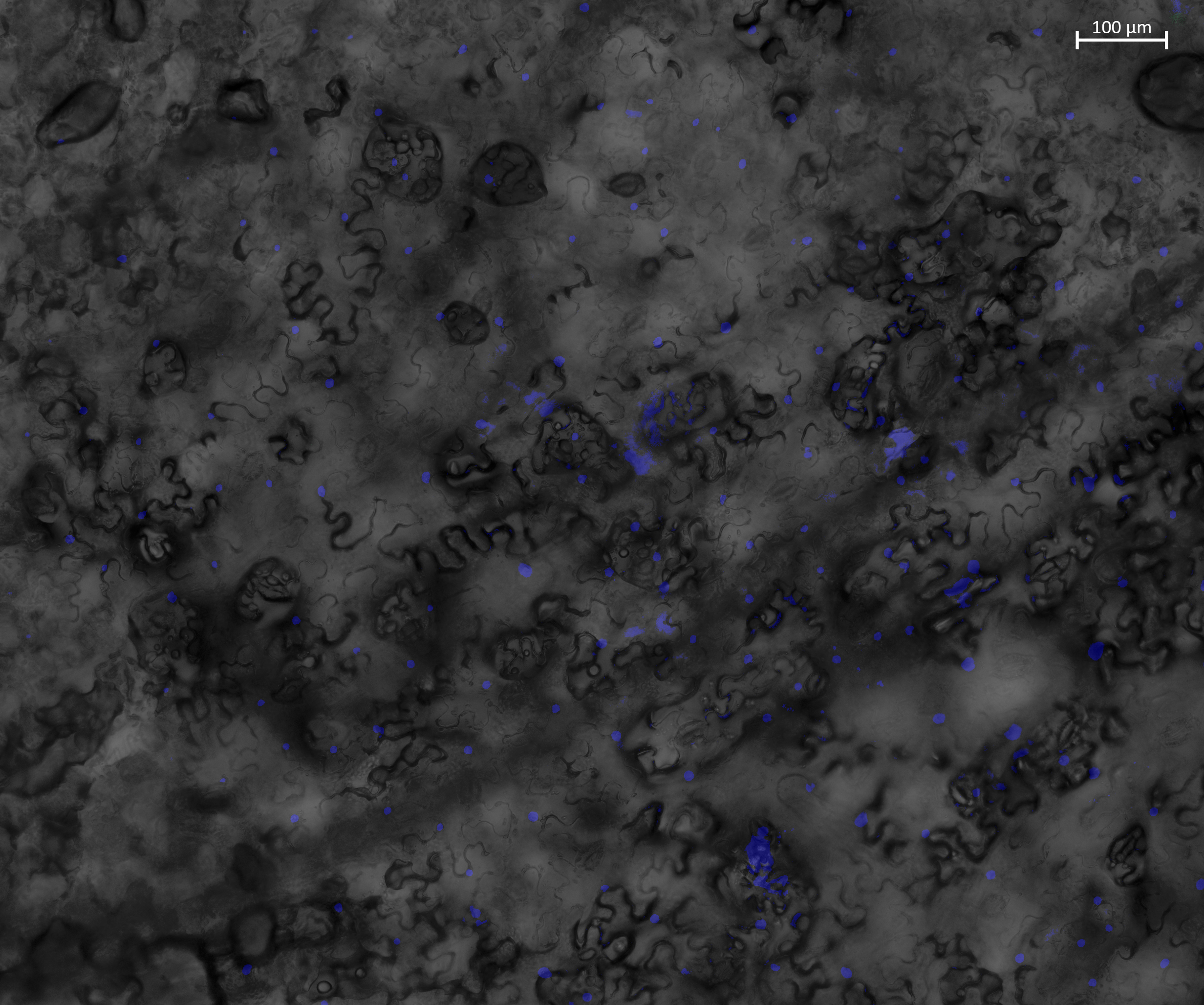

Supplement: Supplementary file 6 — Source Data Fig. 3 [file 44319_2023_30_MOESM6_ESM.zip › Fig.3C/Merge (6xMS2 DIP1-nYFP cYFP-MSCP).tif]

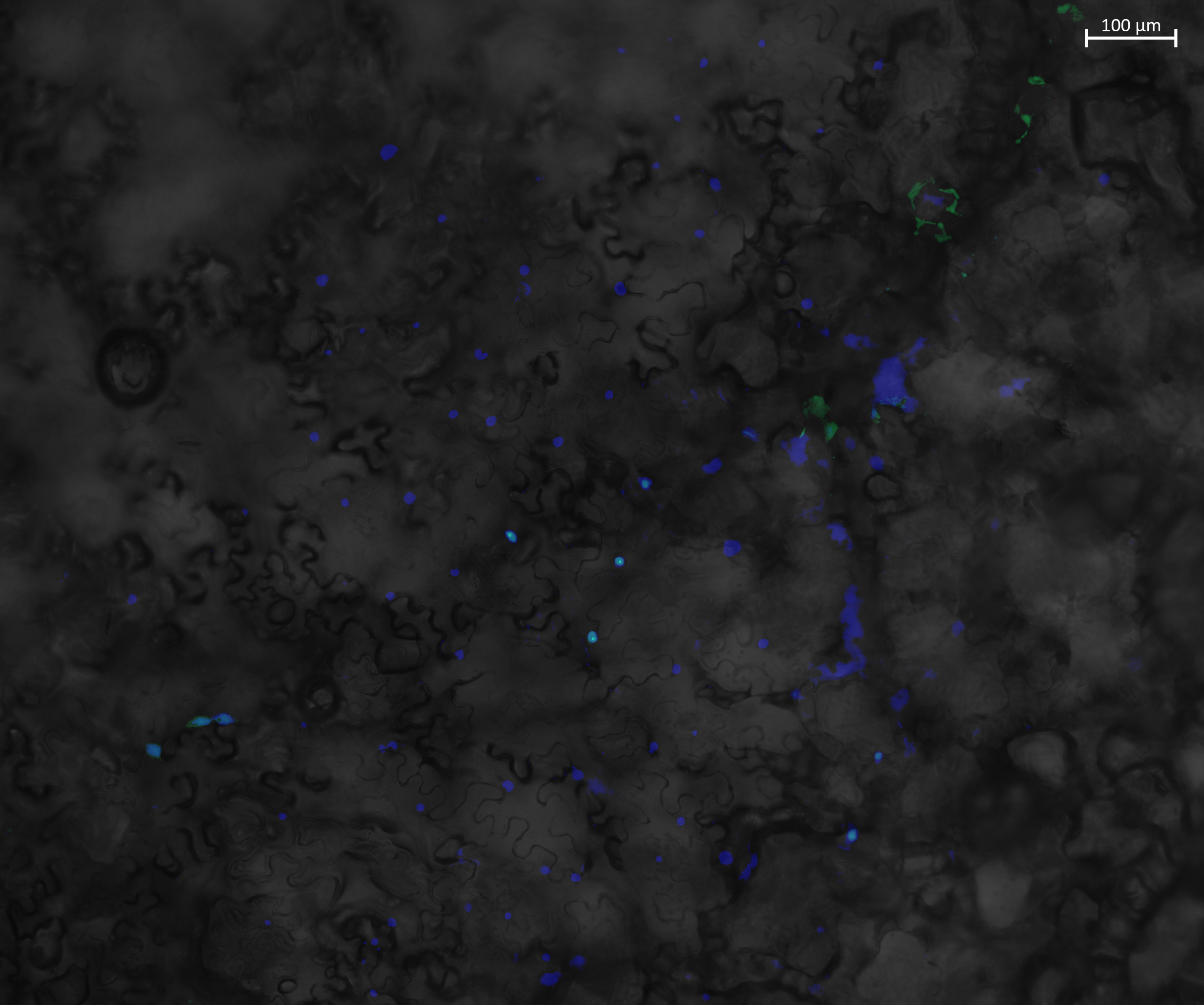

Supplement: Supplementary file 6 — Source Data Fig. 3 [file 44319_2023_30_MOESM6_ESM.zip › Fig.3C/Merge (6xMS2-DANA1 DIP1-nYFP cYFP-MSCP).tif]

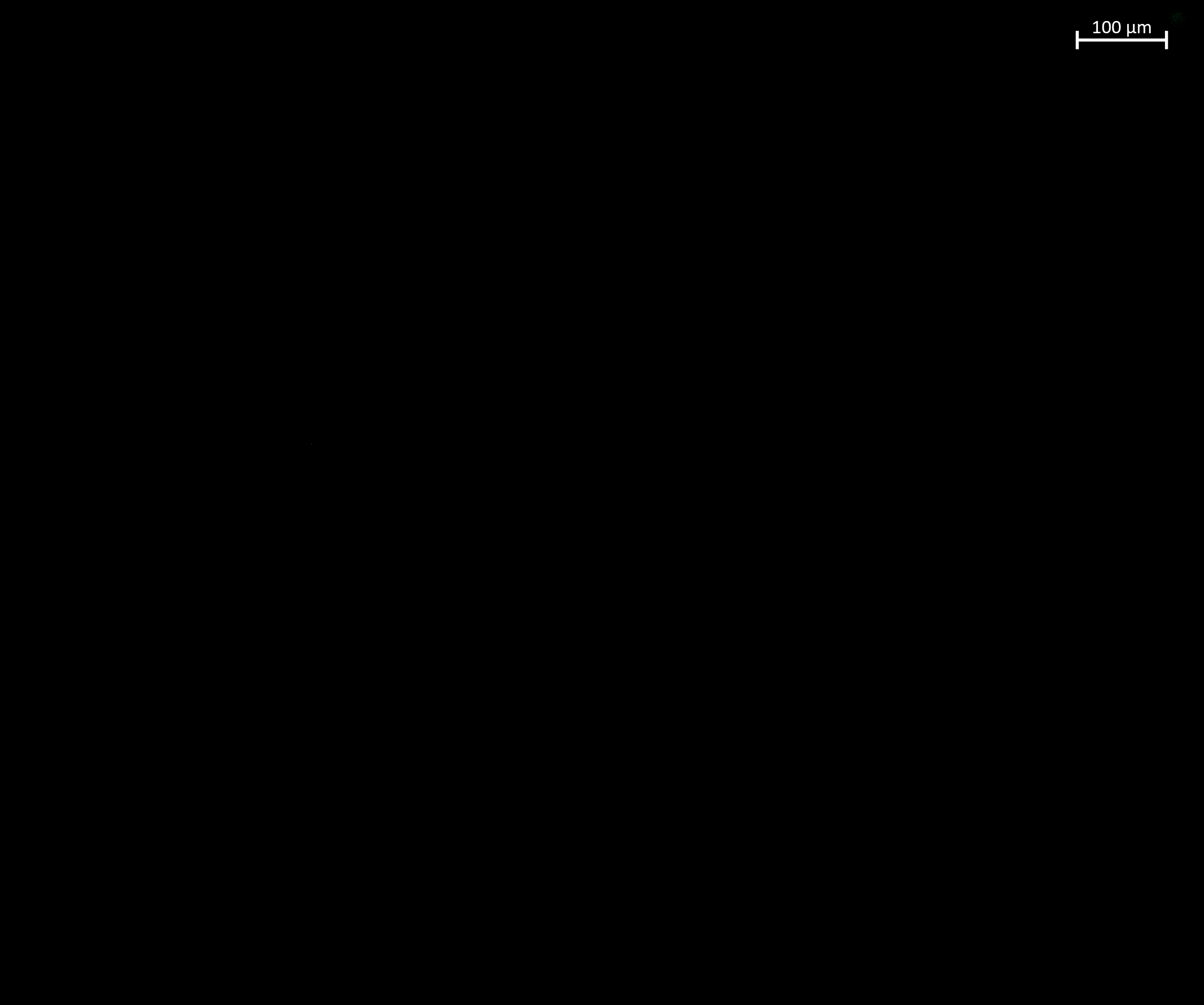

Supplement: Supplementary file 6 — Source Data Fig. 3 [file 44319_2023_30_MOESM6_ESM.zip › Fig.3C/YFP (6xMS2 DIP1-nYFP cYFP-MSCP).tif]

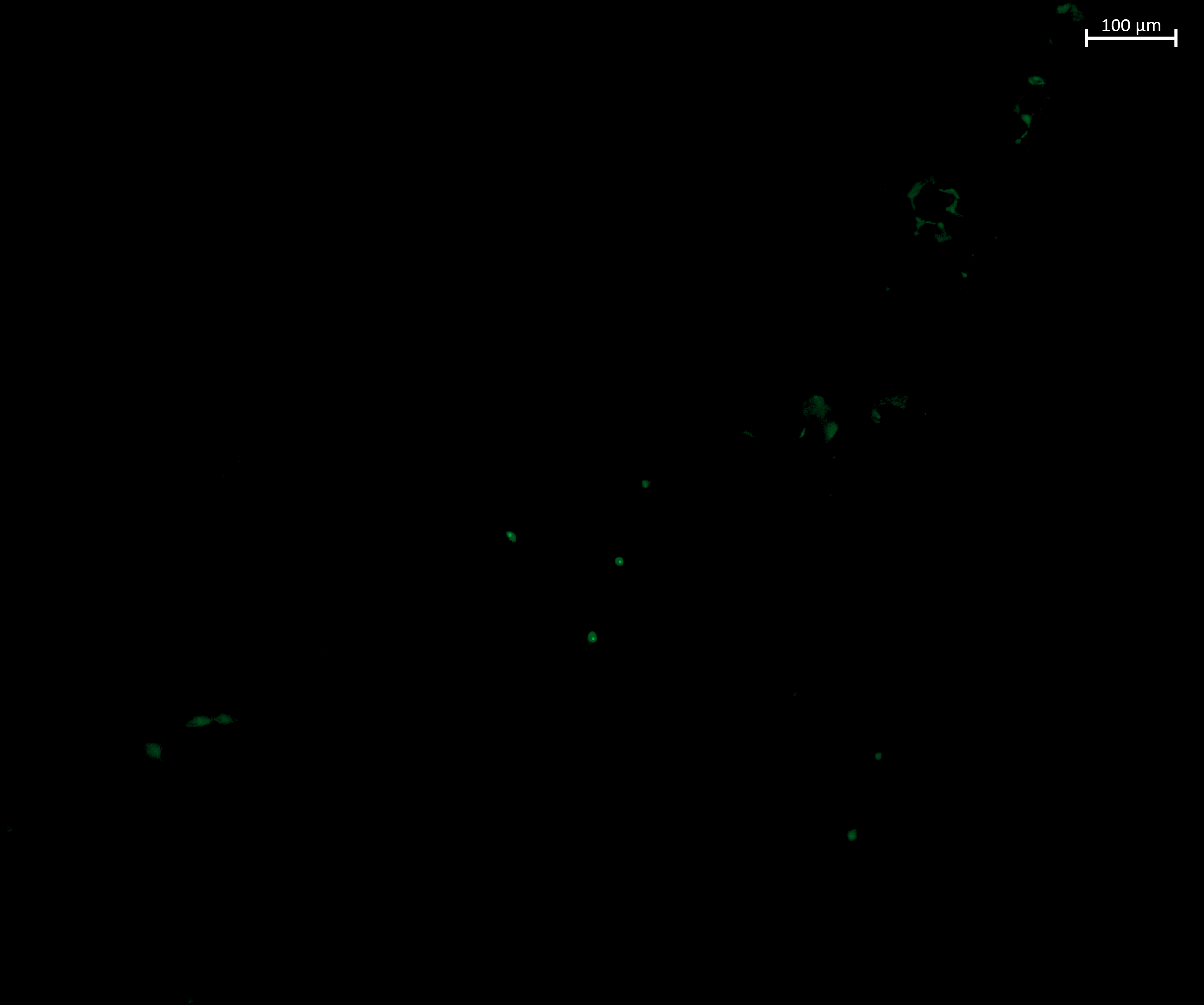

Supplement: Supplementary file 6 — Source Data Fig. 3 [file 44319_2023_30_MOESM6_ESM.zip › Fig.3C/YFP (6xMS2-DANA1 DIP1-nYFP cYFP-MSCP).tif]

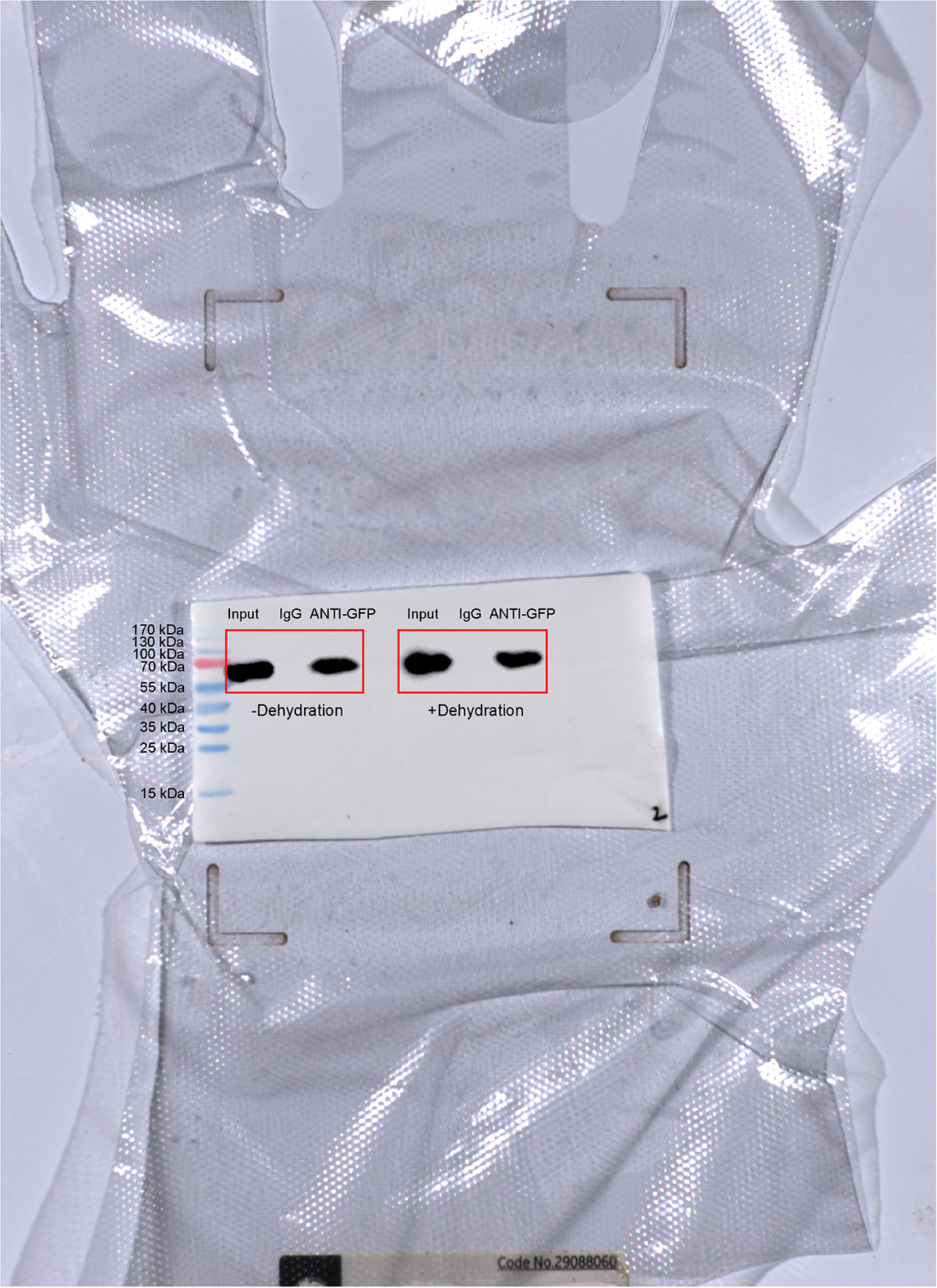

Supplement: Supplementary file 6 — Source Data Fig. 3 [file 44319_2023_30_MOESM6_ESM.zip › Fig.3D/Fig.3D.tif]

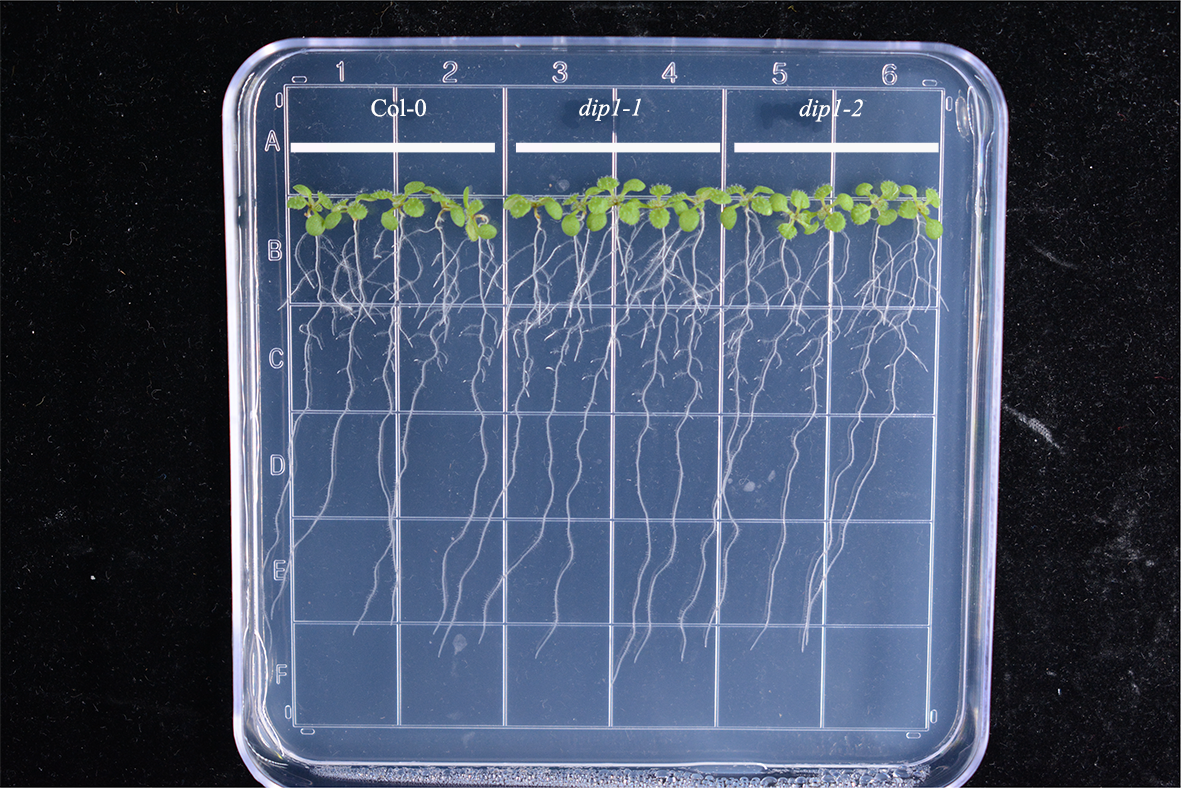

Supplement: Supplementary file 6 — Source Data Fig. 3 [file 44319_2023_30_MOESM6_ESM.zip › Fig.3E/1-2 MS.tif]

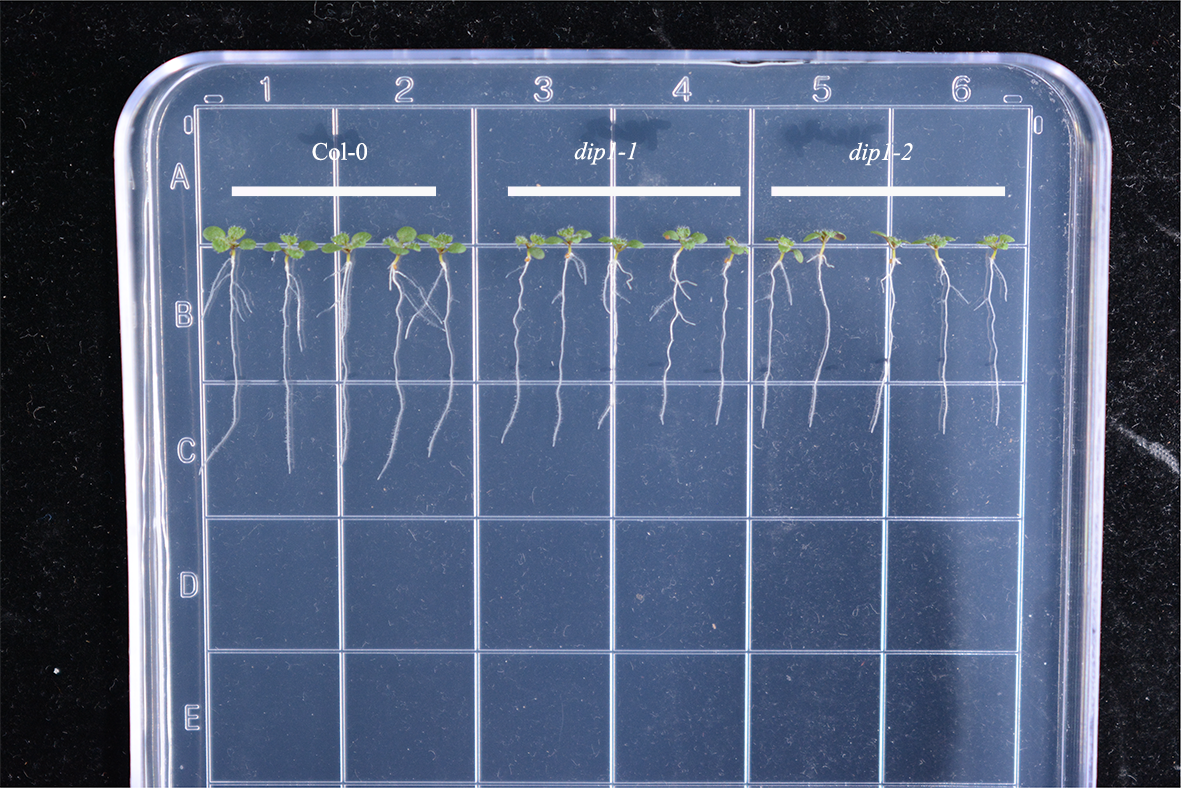

Supplement: Supplementary file 6 — Source Data Fig. 3 [file 44319_2023_30_MOESM6_ESM.zip › Fig.3E/20% PEG.tif]

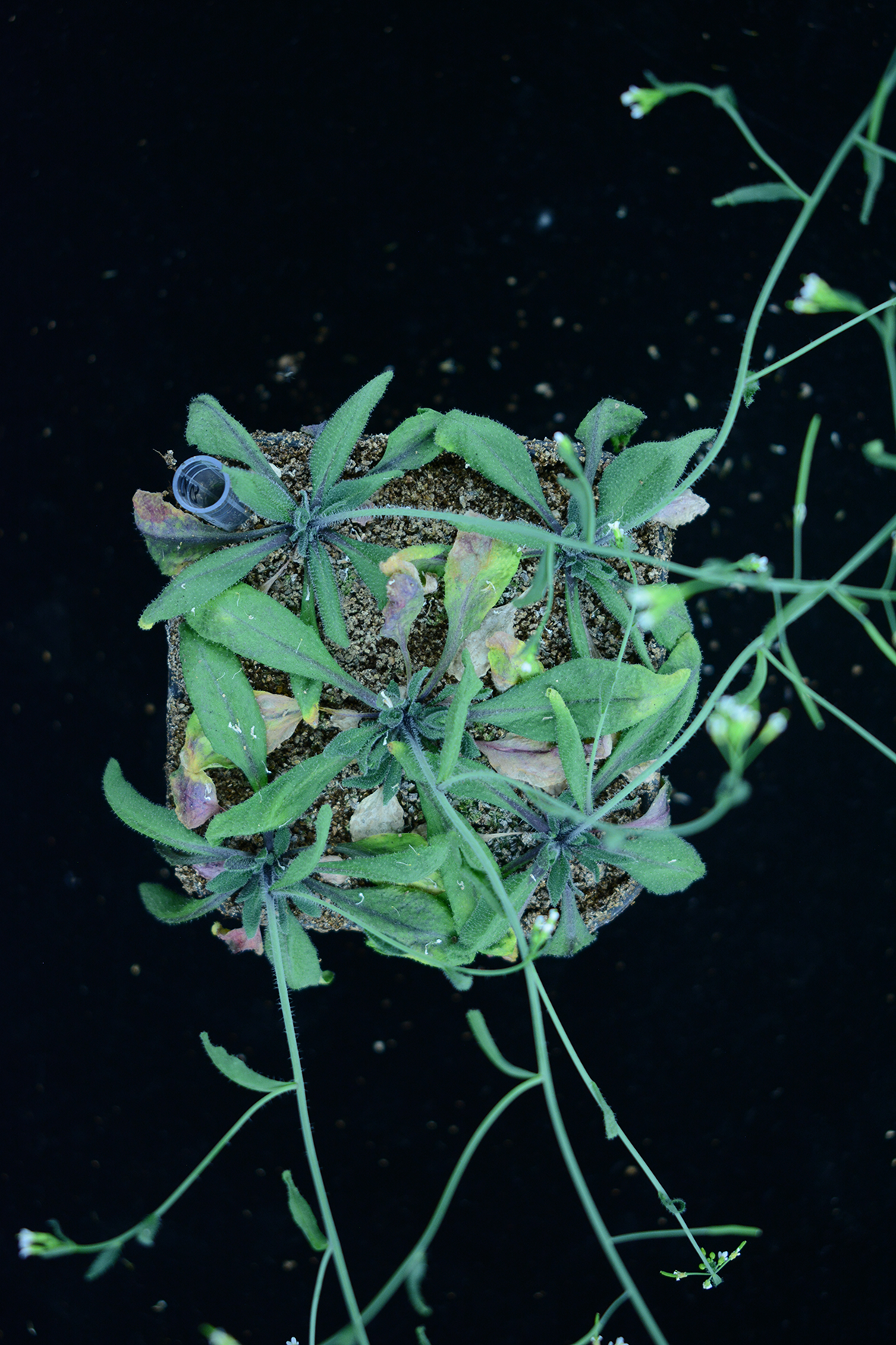

Supplement: Supplementary file 6 — Source Data Fig. 3 [file 44319_2023_30_MOESM6_ESM.zip › Fig.3G/Col-0 drought for fourteen days.tif]

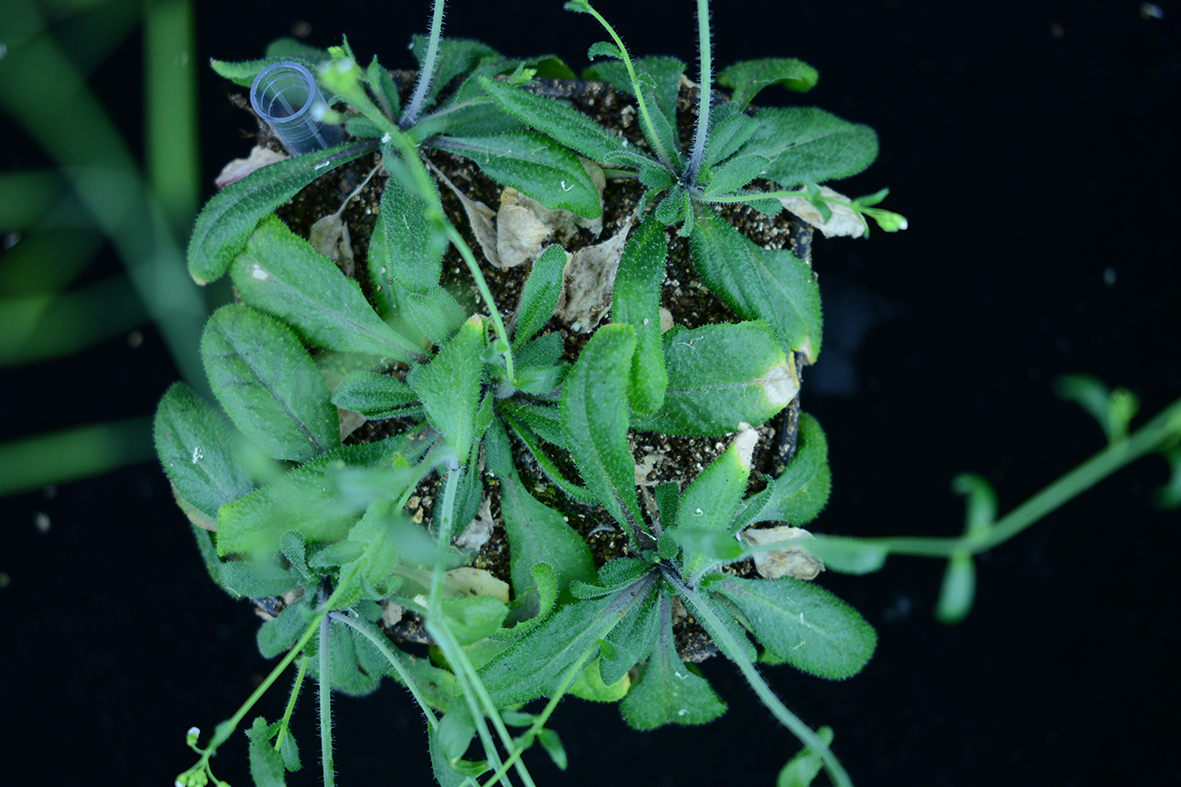

Supplement: Supplementary file 6 — Source Data Fig. 3 [file 44319_2023_30_MOESM6_ESM.zip › Fig.3G/Col-0 five days after rewatering.tif]

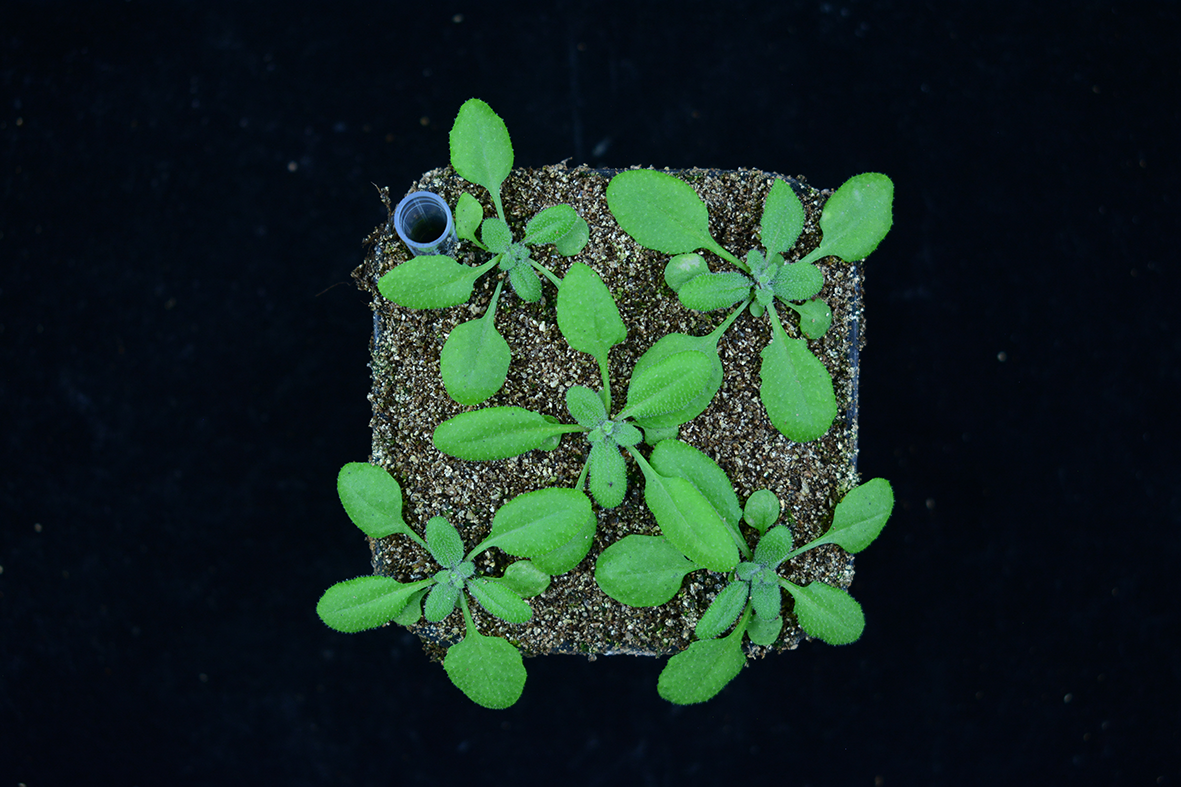

Supplement: Supplementary file 6 — Source Data Fig. 3 [file 44319_2023_30_MOESM6_ESM.zip › Fig.3G/Col-0 three-week-old seedlings.tif]

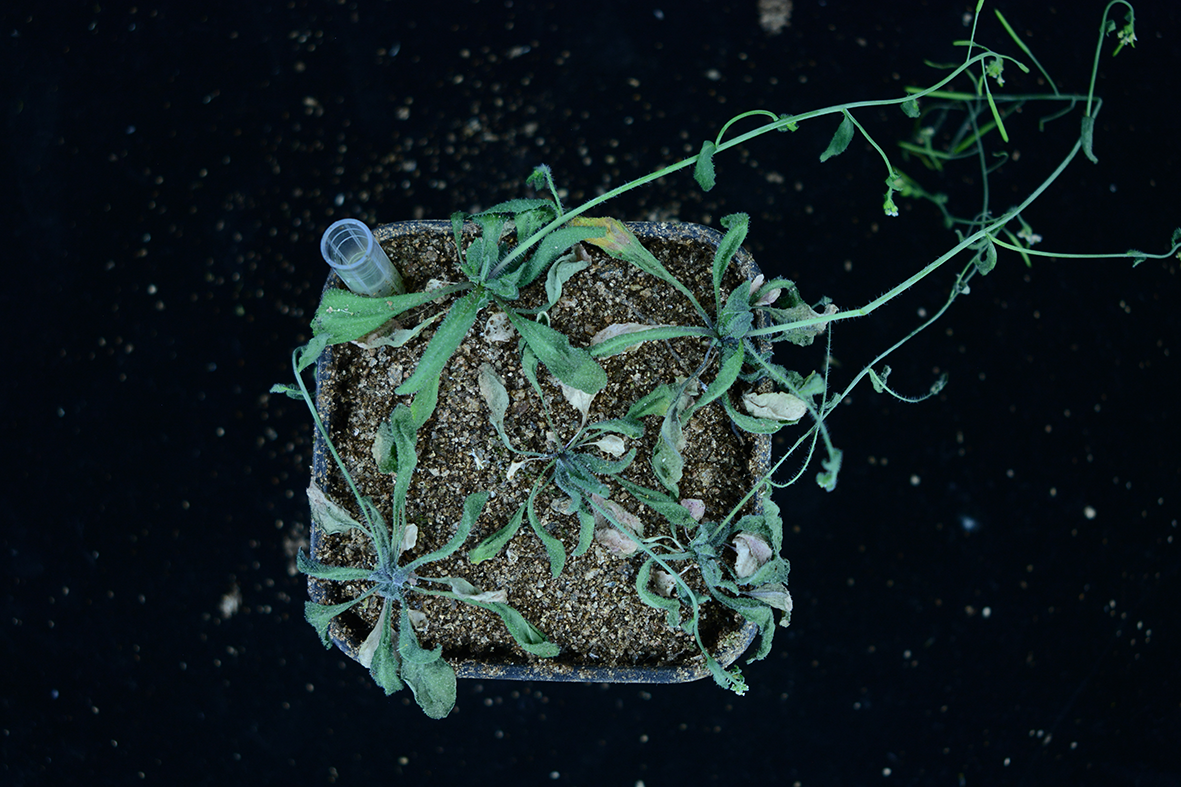

Supplement: Supplementary file 6 — Source Data Fig. 3 [file 44319_2023_30_MOESM6_ESM.zip › Fig.3G/dip1-1 drought for fourteen days.tif]

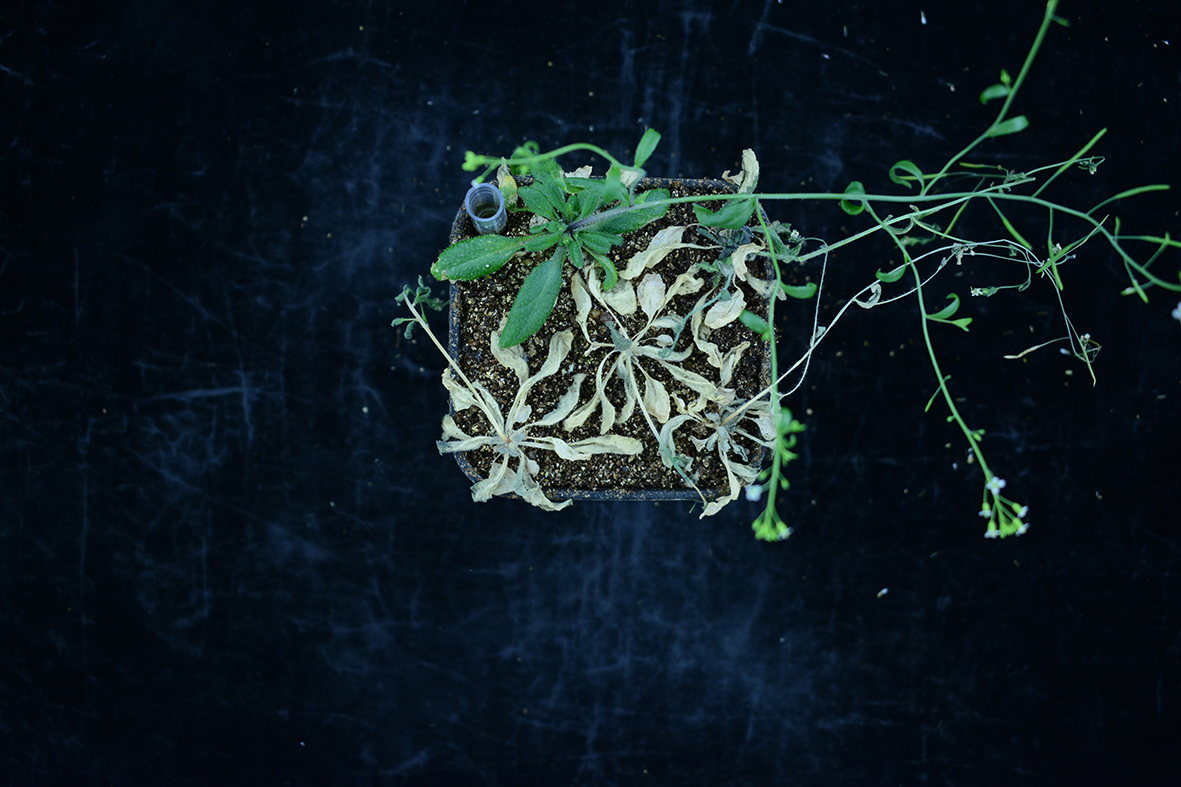

Supplement: Supplementary file 6 — Source Data Fig. 3 [file 44319_2023_30_MOESM6_ESM.zip › Fig.3G/dip1-1 five days after rewatering.tif]

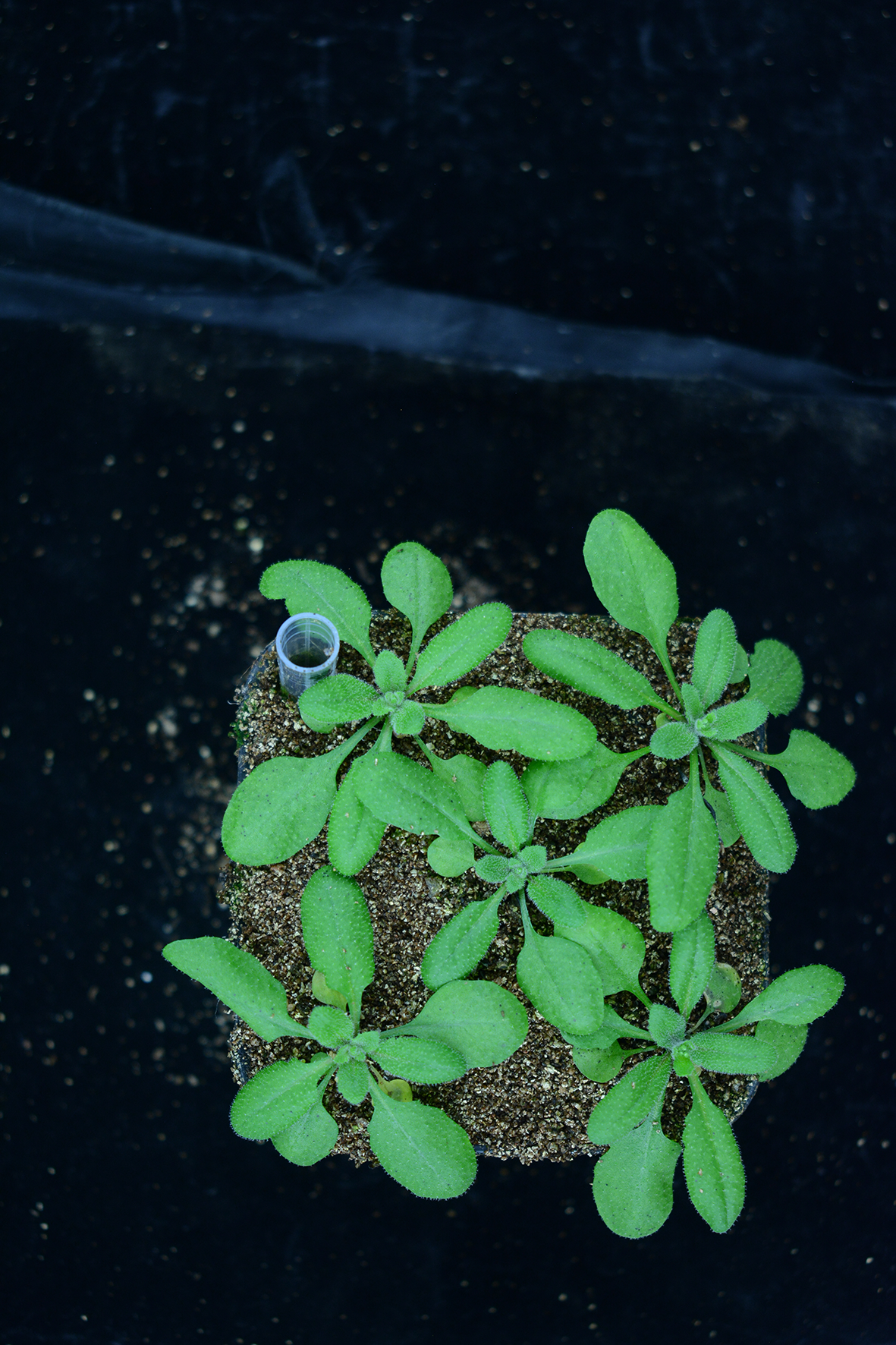

Supplement: Supplementary file 6 — Source Data Fig. 3 [file 44319_2023_30_MOESM6_ESM.zip › Fig.3G/dip1-1 three-week-old seedlings.tif]

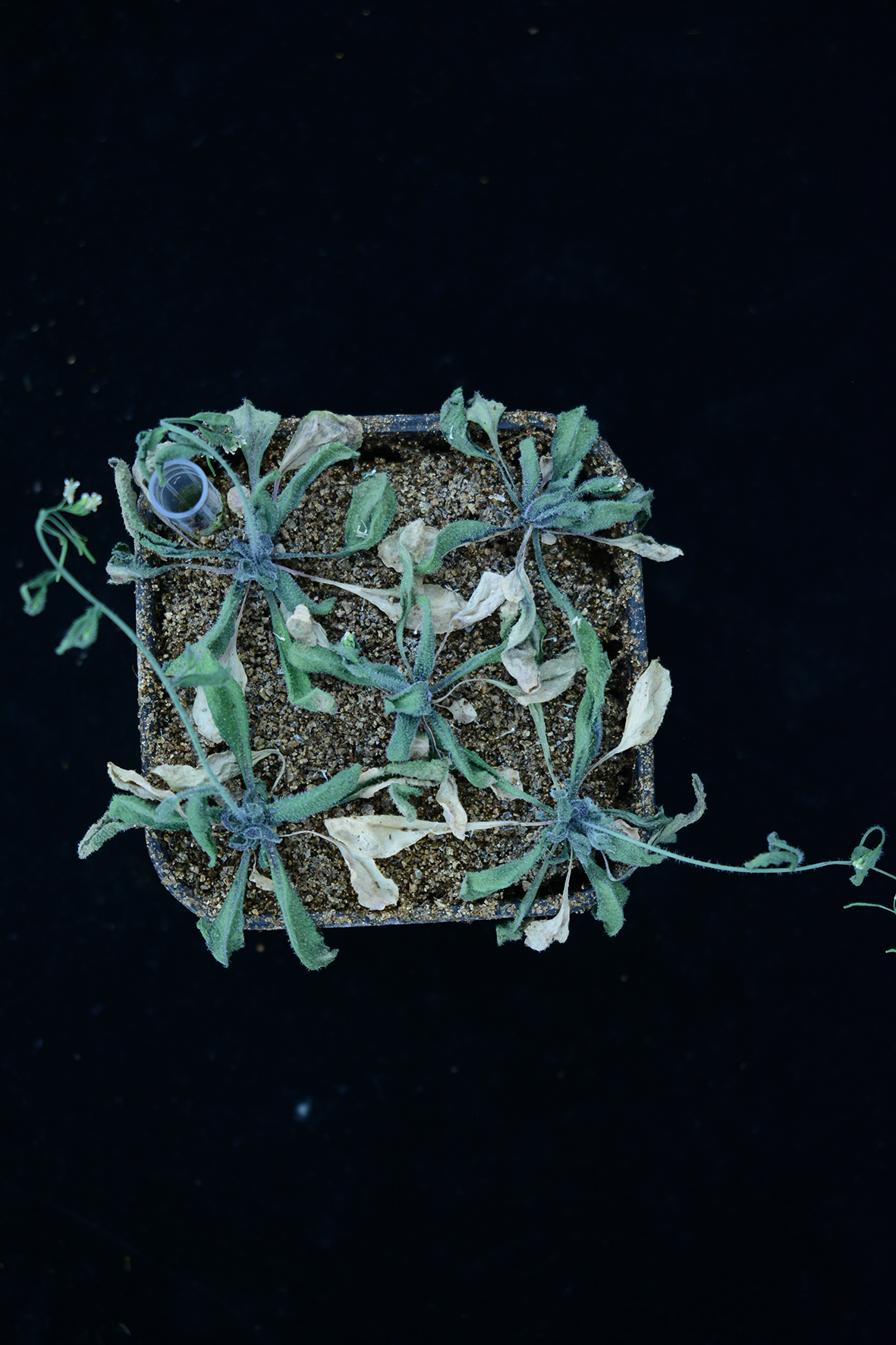

Supplement: Supplementary file 6 — Source Data Fig. 3 [file 44319_2023_30_MOESM6_ESM.zip › Fig.3G/dip1-2 drought for fourteen days.tif]

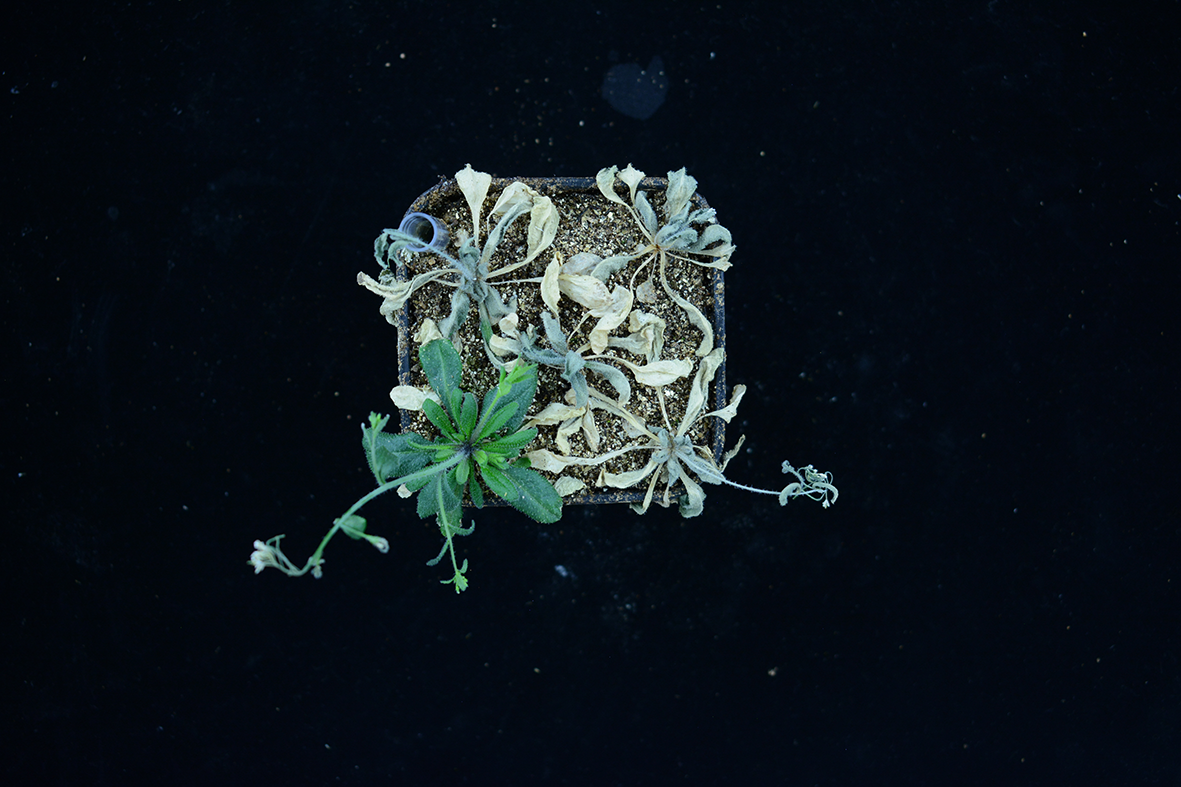

Supplement: Supplementary file 6 — Source Data Fig. 3 [file 44319_2023_30_MOESM6_ESM.zip › Fig.3G/dip1-2 five days after rewatering.tif]

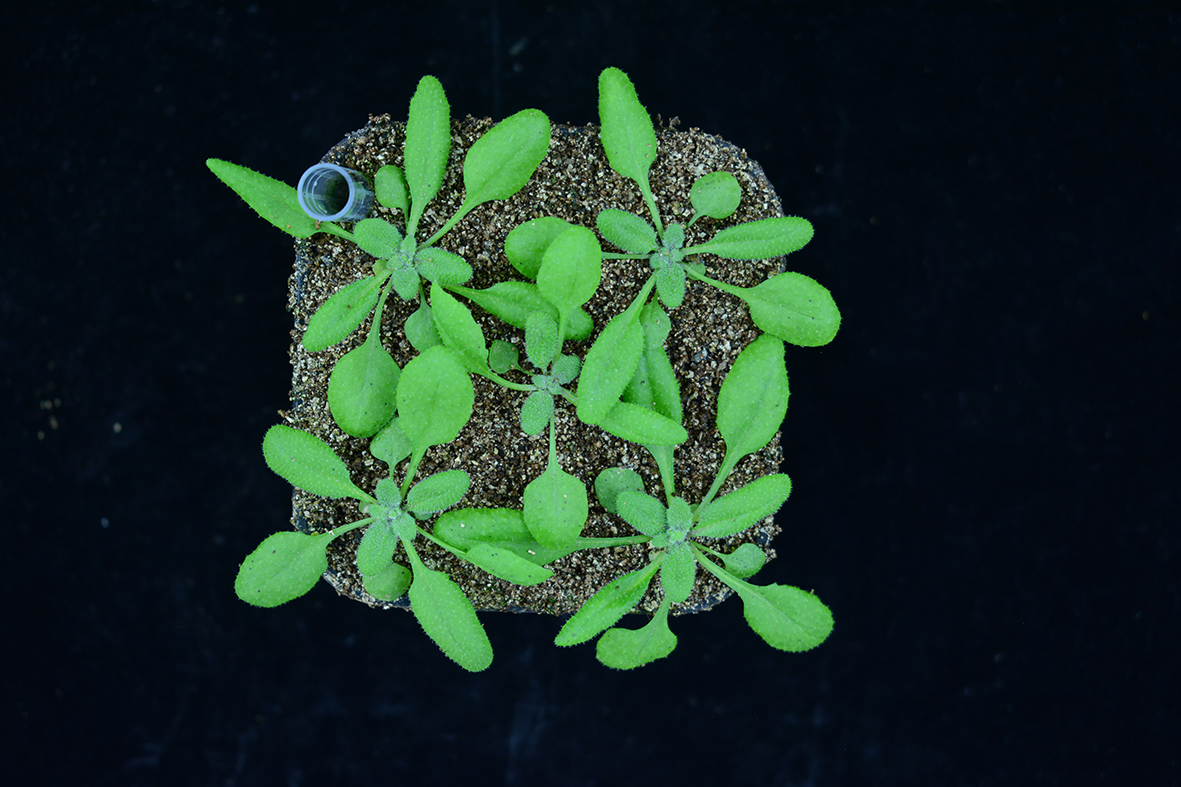

Supplement: Supplementary file 6 — Source Data Fig. 3 [file 44319_2023_30_MOESM6_ESM.zip › Fig.3G/dip1-2 three-week-old seedlings.tif]

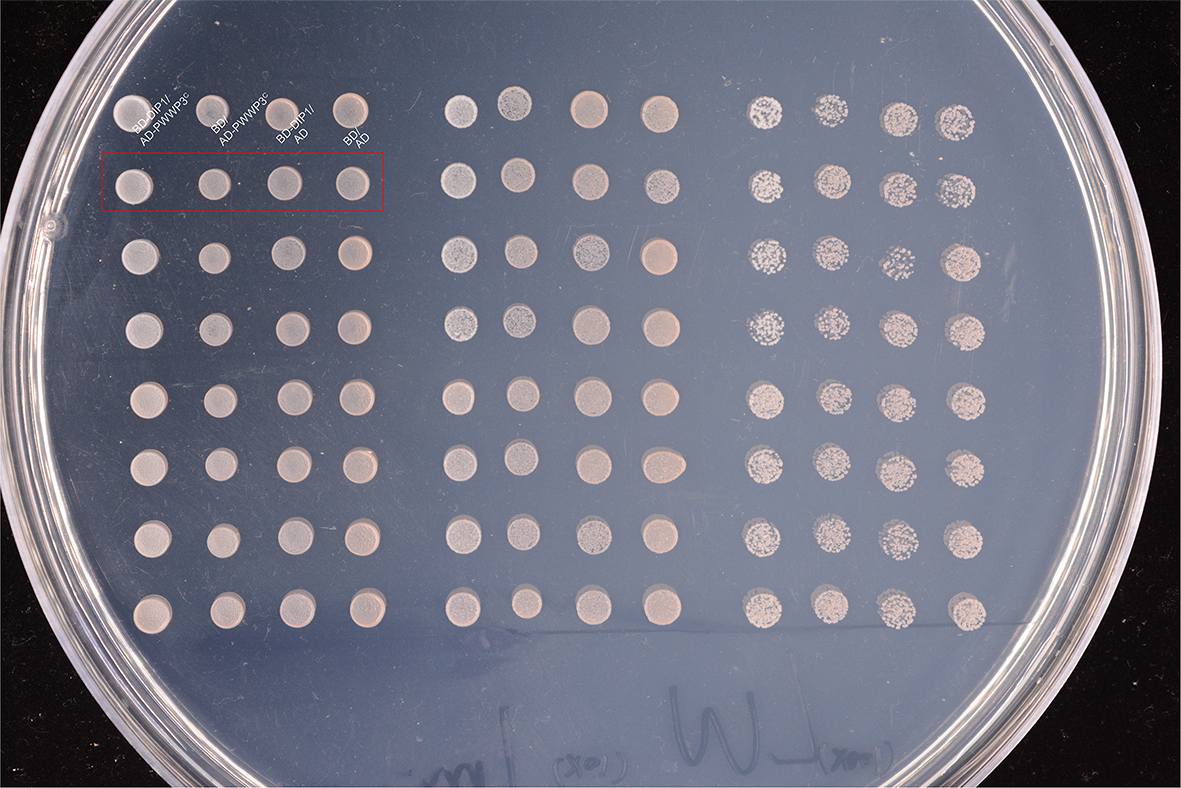

Supplement: Supplementary file 7 — Source Data Fig. 4 [file 44319_2023_30_MOESM7_ESM.zip › Fig.4A/-WL.tif]

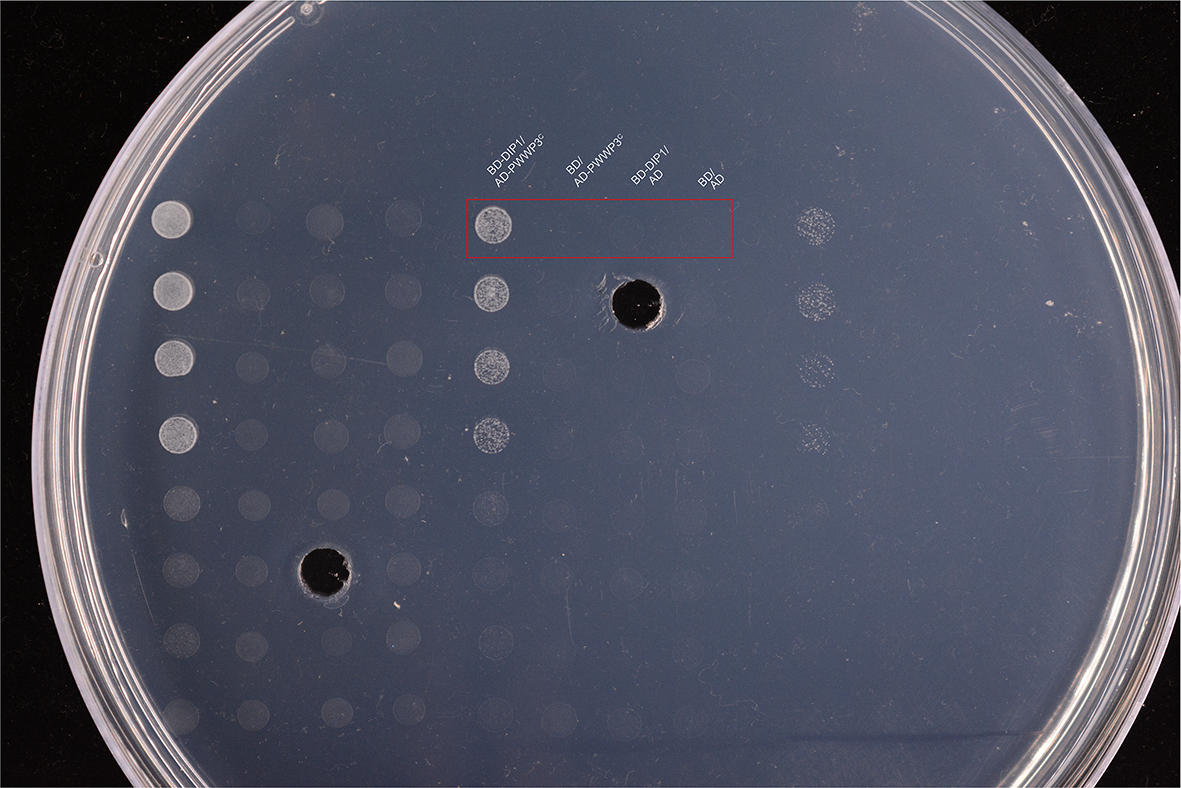

Supplement: Supplementary file 7 — Source Data Fig. 4 [file 44319_2023_30_MOESM7_ESM.zip › Fig.4A/-WLHA.tif]

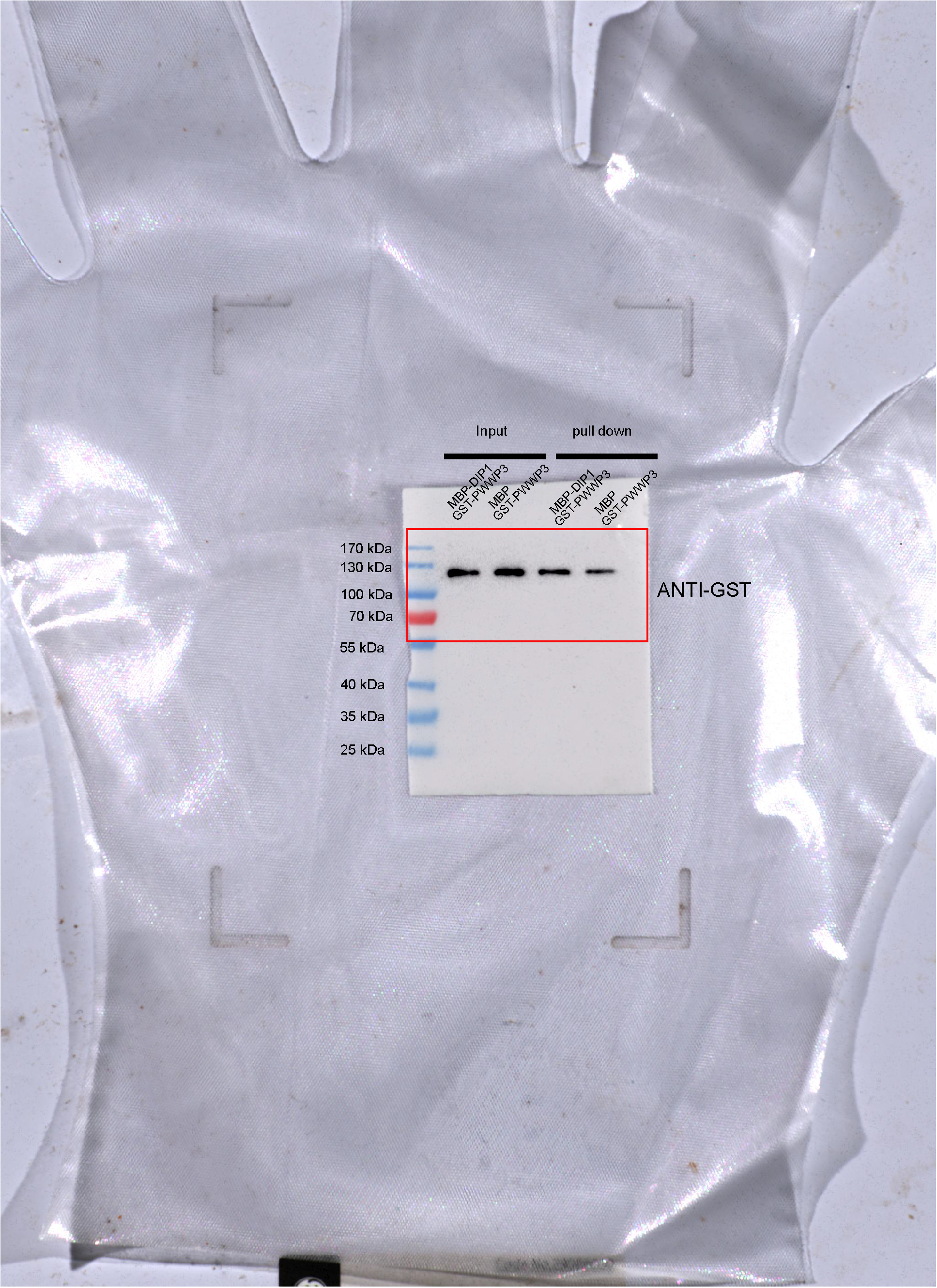

Supplement: Supplementary file 7 — Source Data Fig. 4 [file 44319_2023_30_MOESM7_ESM.zip › Fig.4B/ANTI-GST.tif]

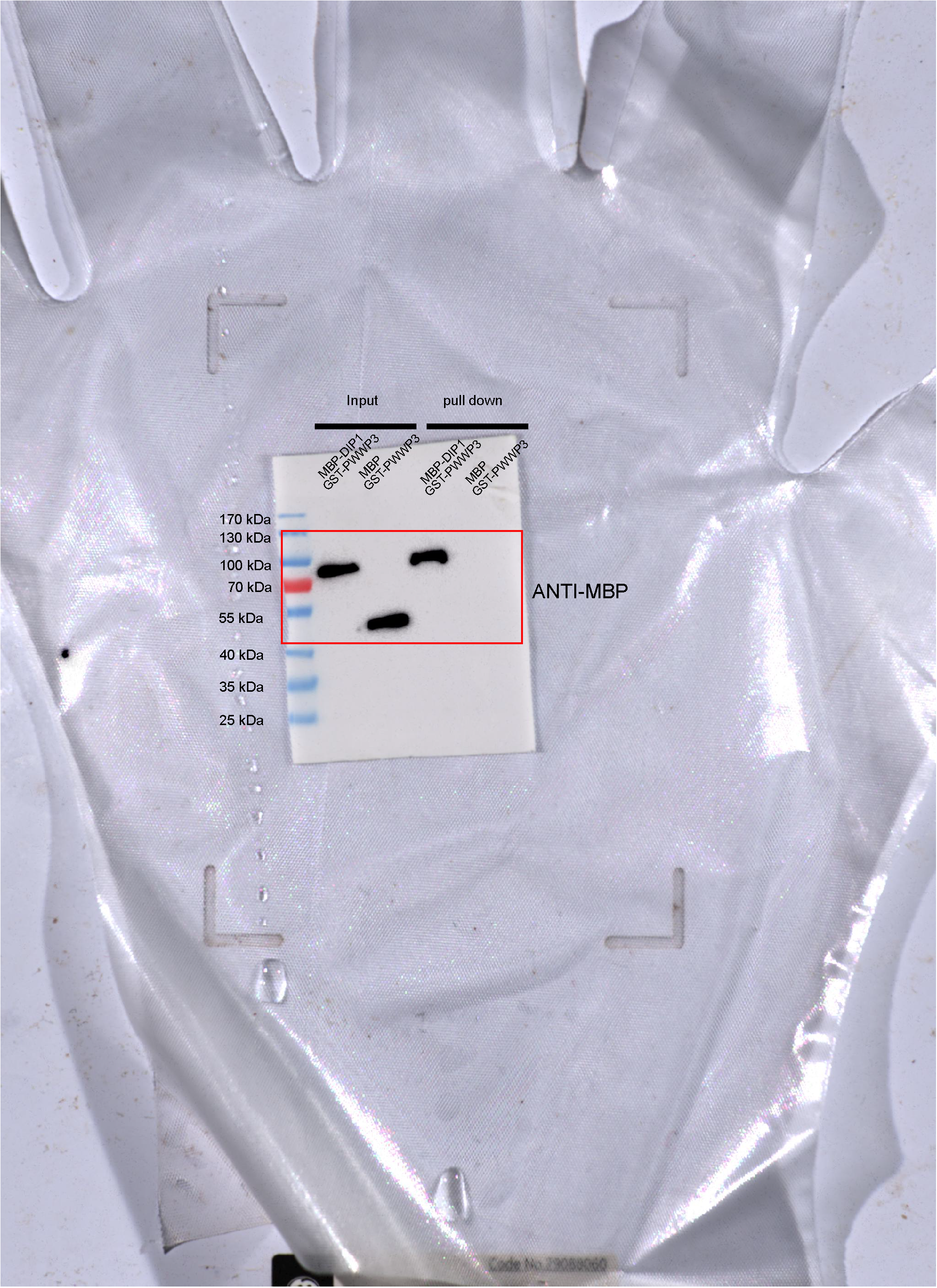

Supplement: Supplementary file 7 — Source Data Fig. 4 [file 44319_2023_30_MOESM7_ESM.zip › Fig.4B/ANTI-MBP.tif]

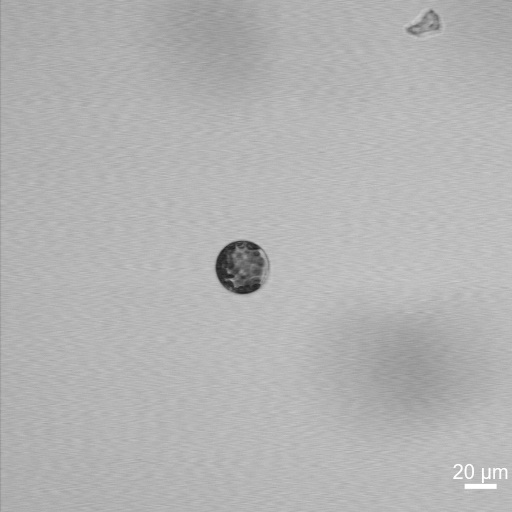

Supplement: Supplementary file 7 — Source Data Fig. 4 [file 44319_2023_30_MOESM7_ESM.zip › Fig.4C/BF (cYFP PWWP3-nYFP).tif]

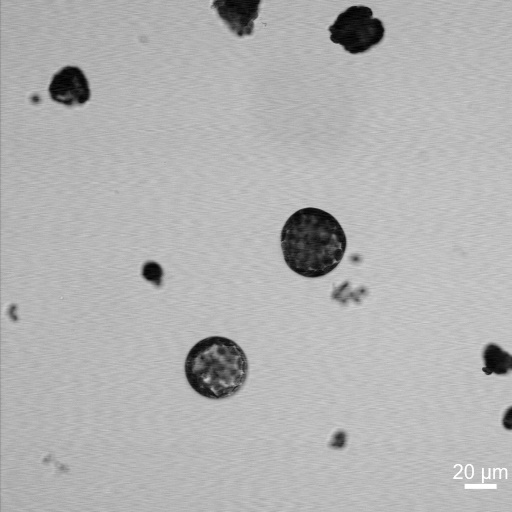

Supplement: Supplementary file 7 — Source Data Fig. 4 [file 44319_2023_30_MOESM7_ESM.zip › Fig.4C/BF (DIP-cYFP nYFP).tif]

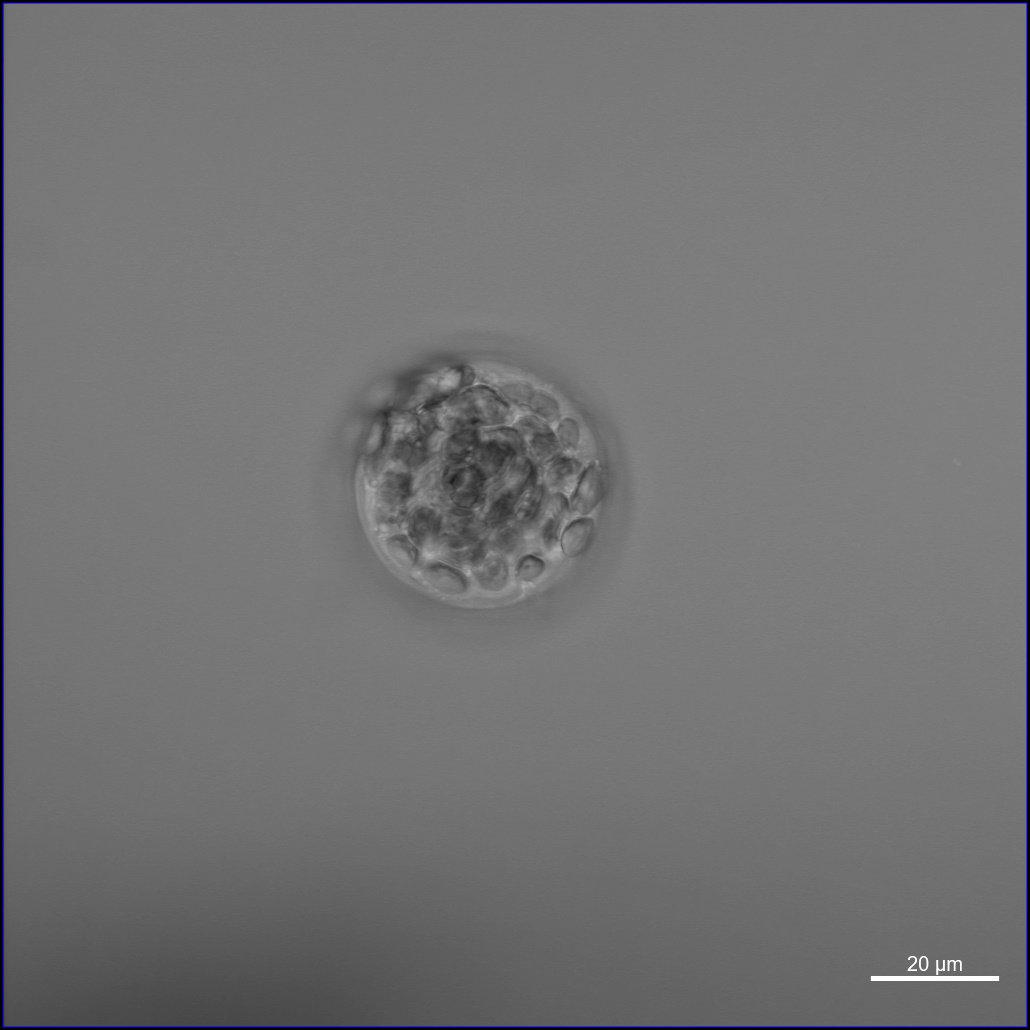

Supplement: Supplementary file 7 — Source Data Fig. 4 [file 44319_2023_30_MOESM7_ESM.zip › Fig.4C/BF (DIP1-cYFP PWWP3-nYFP).tif]

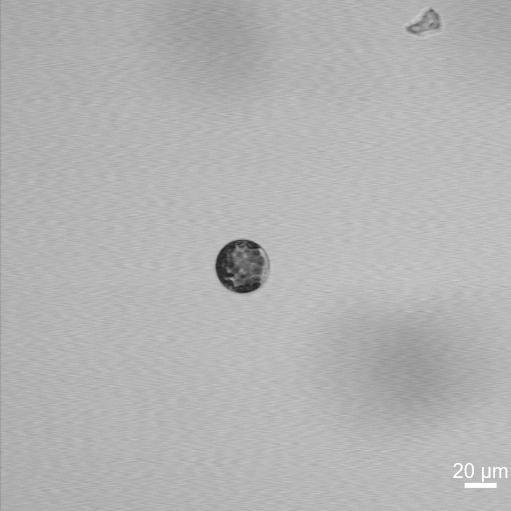

Supplement: Supplementary file 7 — Source Data Fig. 4 [file 44319_2023_30_MOESM7_ESM.zip › Fig.4C/Merge (cYFP PWWP3-nYFP).tif]

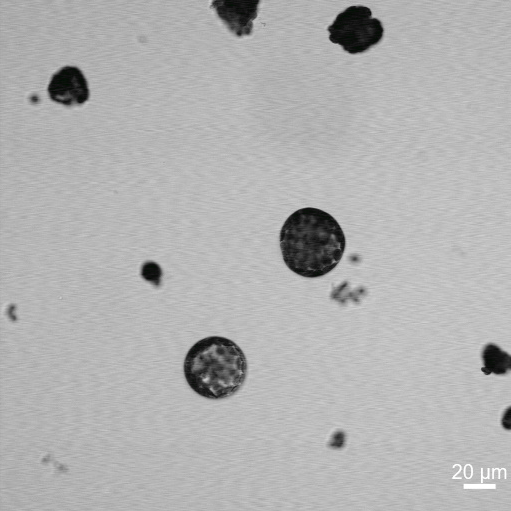

Supplement: Supplementary file 7 — Source Data Fig. 4 [file 44319_2023_30_MOESM7_ESM.zip › Fig.4C/Merge (DIP1-cYFP nYFP).tif]

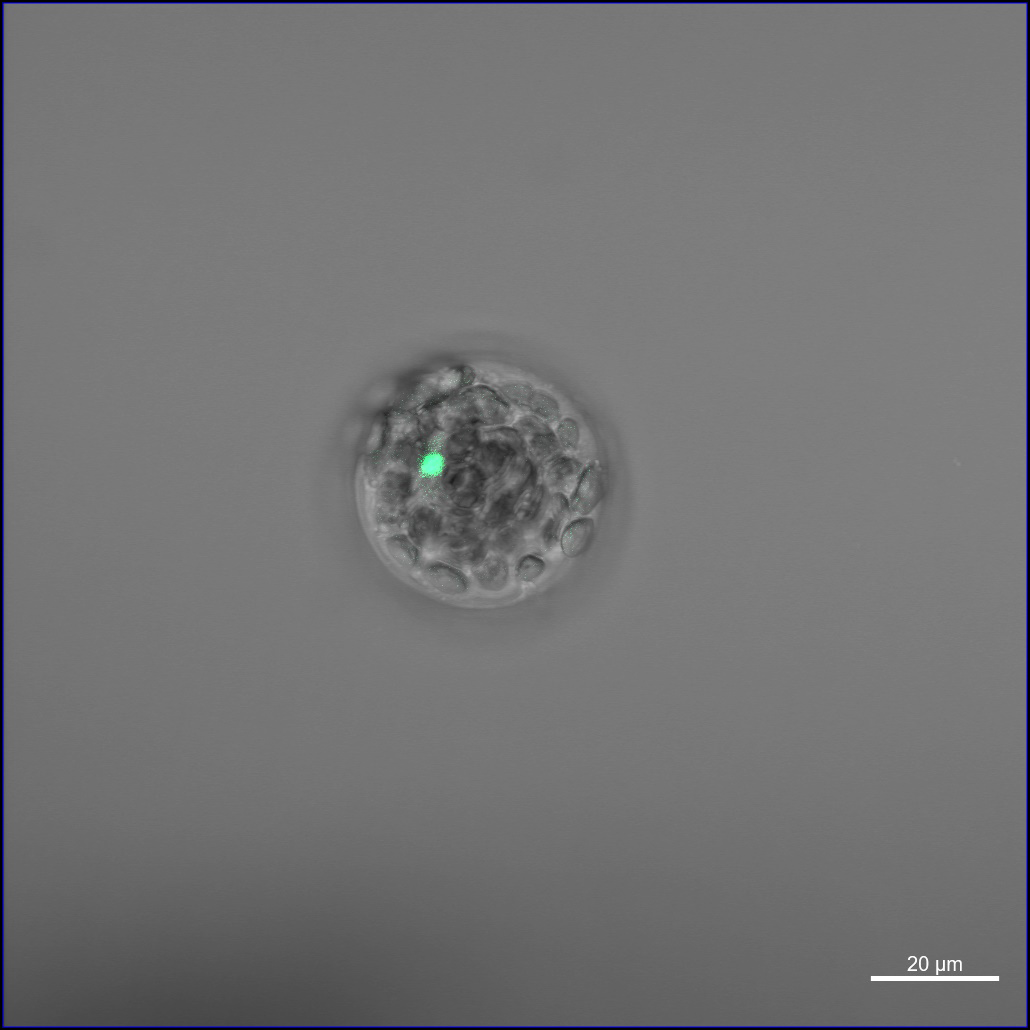

Supplement: Supplementary file 7 — Source Data Fig. 4 [file 44319_2023_30_MOESM7_ESM.zip › Fig.4C/Merge (DIP1-cYFP PWWP3-nYFP).tif]

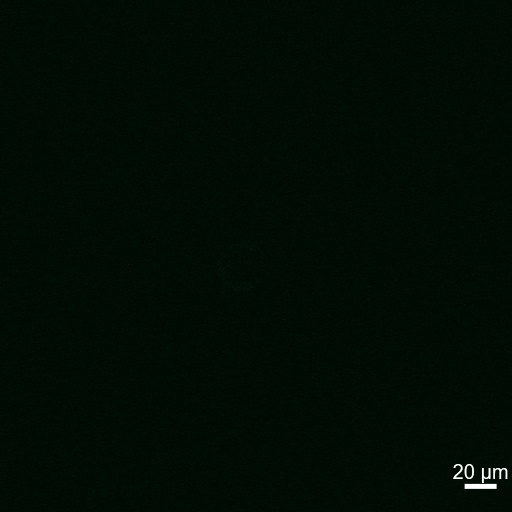

Supplement: Supplementary file 7 — Source Data Fig. 4 [file 44319_2023_30_MOESM7_ESM.zip › Fig.4C/YFP (cYFP PWWP3-nYFP).tif]

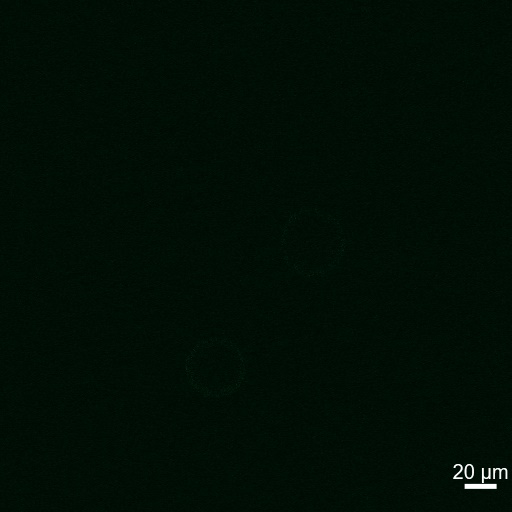

Supplement: Supplementary file 7 — Source Data Fig. 4 [file 44319_2023_30_MOESM7_ESM.zip › Fig.4C/YFP (DIP-cYFP nYFP).tif]

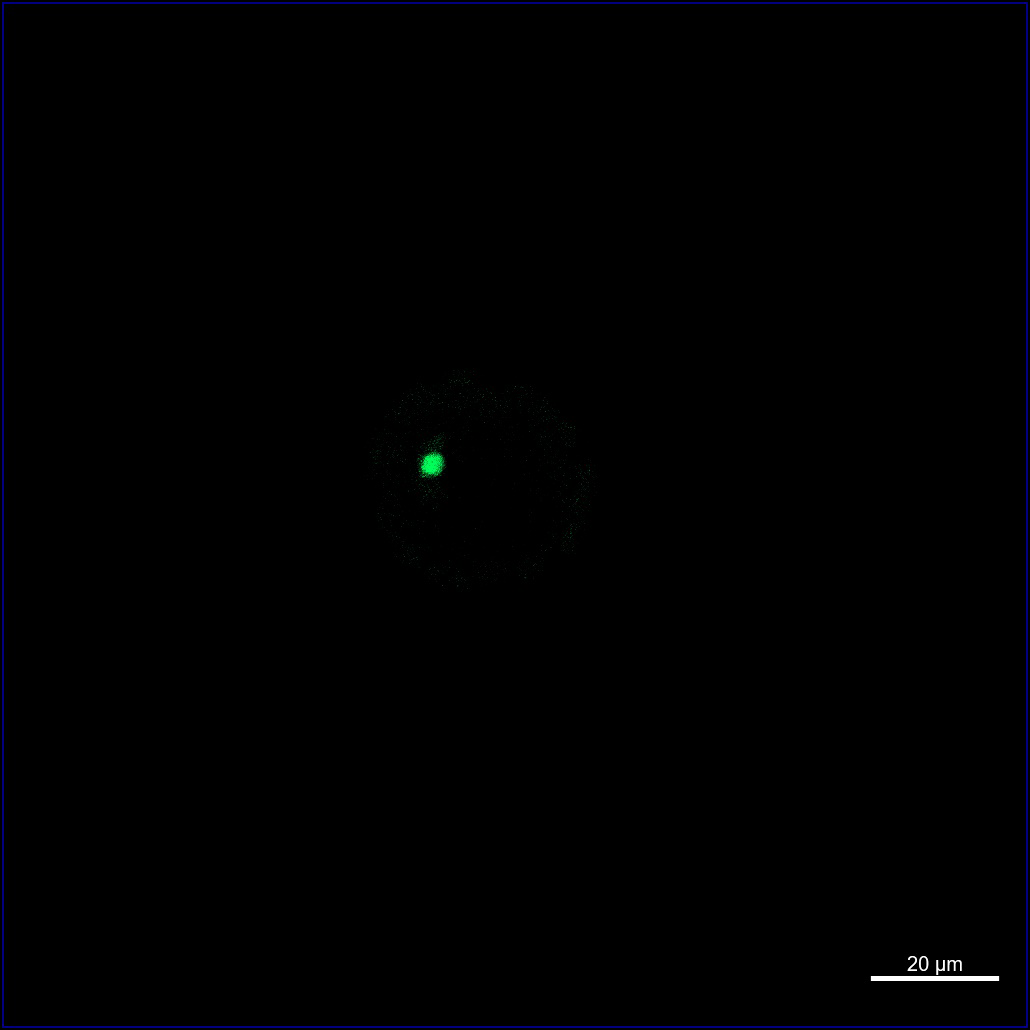

Supplement: Supplementary file 7 — Source Data Fig. 4 [file 44319_2023_30_MOESM7_ESM.zip › Fig.4C/YFP (DIP1-cYFP PWWP3-nYFP).tif]

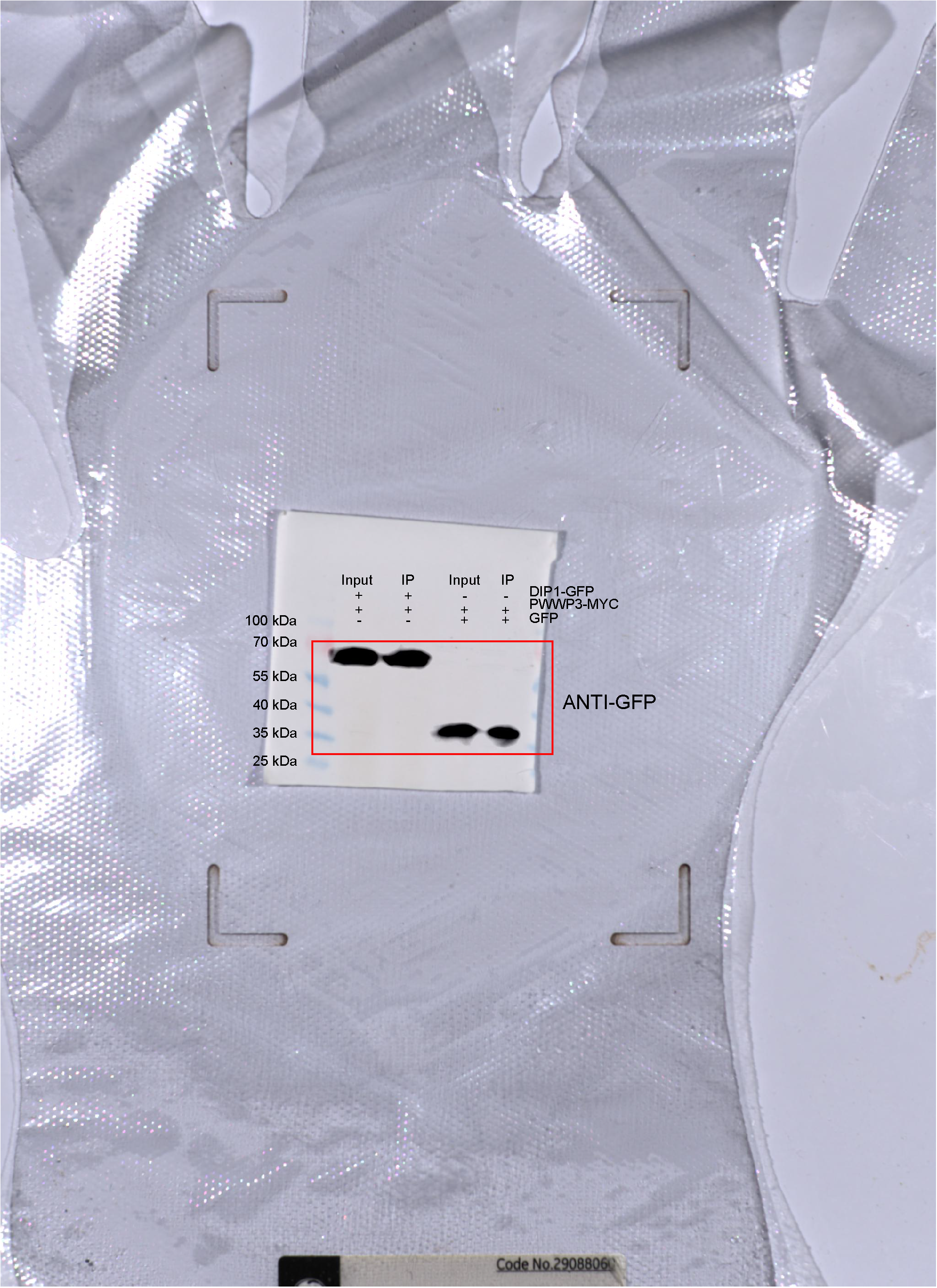

Supplement: Supplementary file 7 — Source Data Fig. 4 [file 44319_2023_30_MOESM7_ESM.zip › Fig.4D/ANTI-GFP.tif]

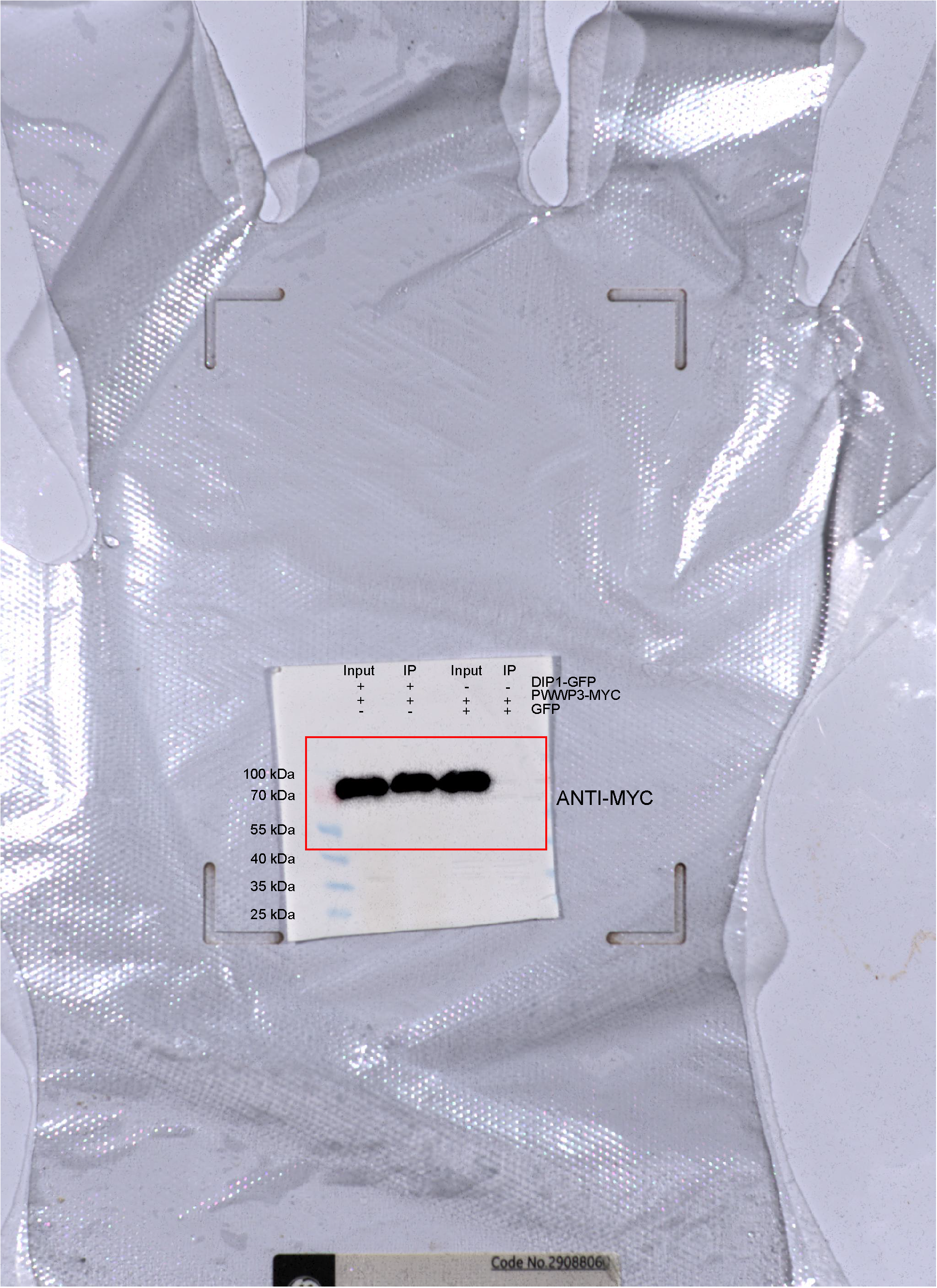

Supplement: Supplementary file 7 — Source Data Fig. 4 [file 44319_2023_30_MOESM7_ESM.zip › Fig.4D/ANTI-MYC.tif]

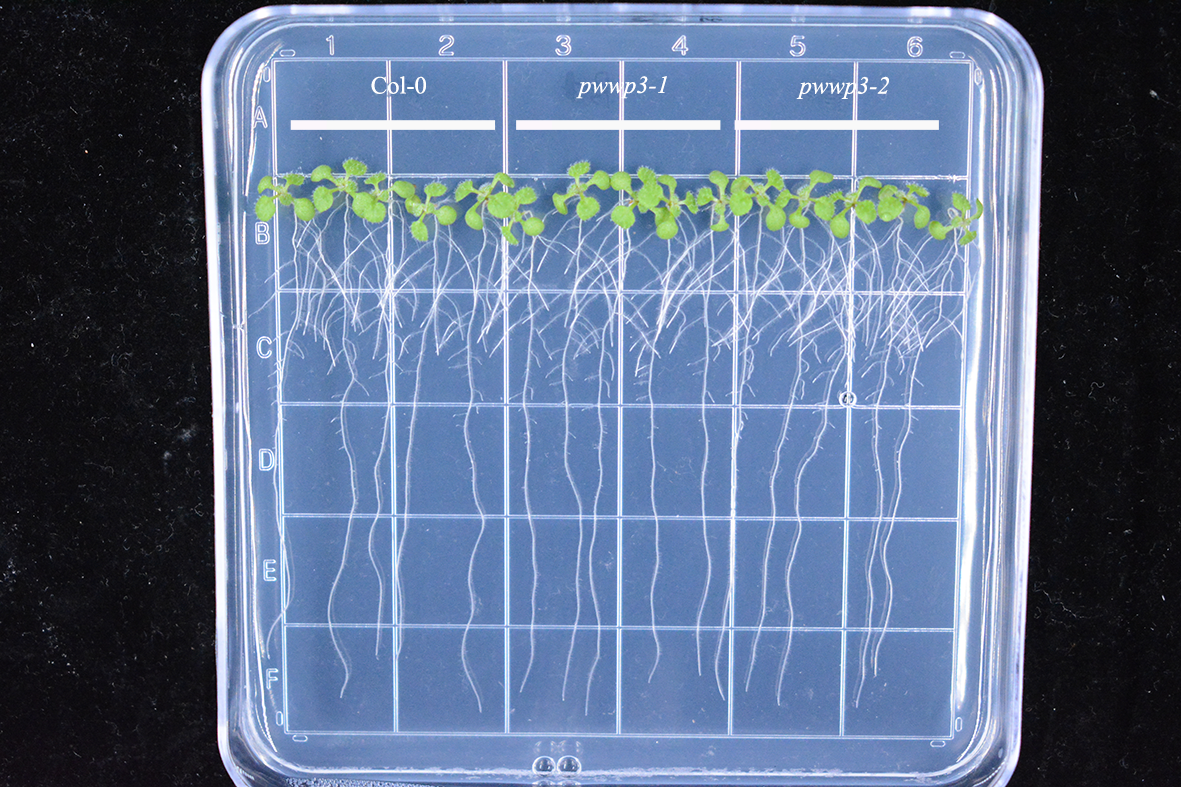

Supplement: Supplementary file 7 — Source Data Fig. 4 [file 44319_2023_30_MOESM7_ESM.zip › Fig.4E/1-2 MS.tif]

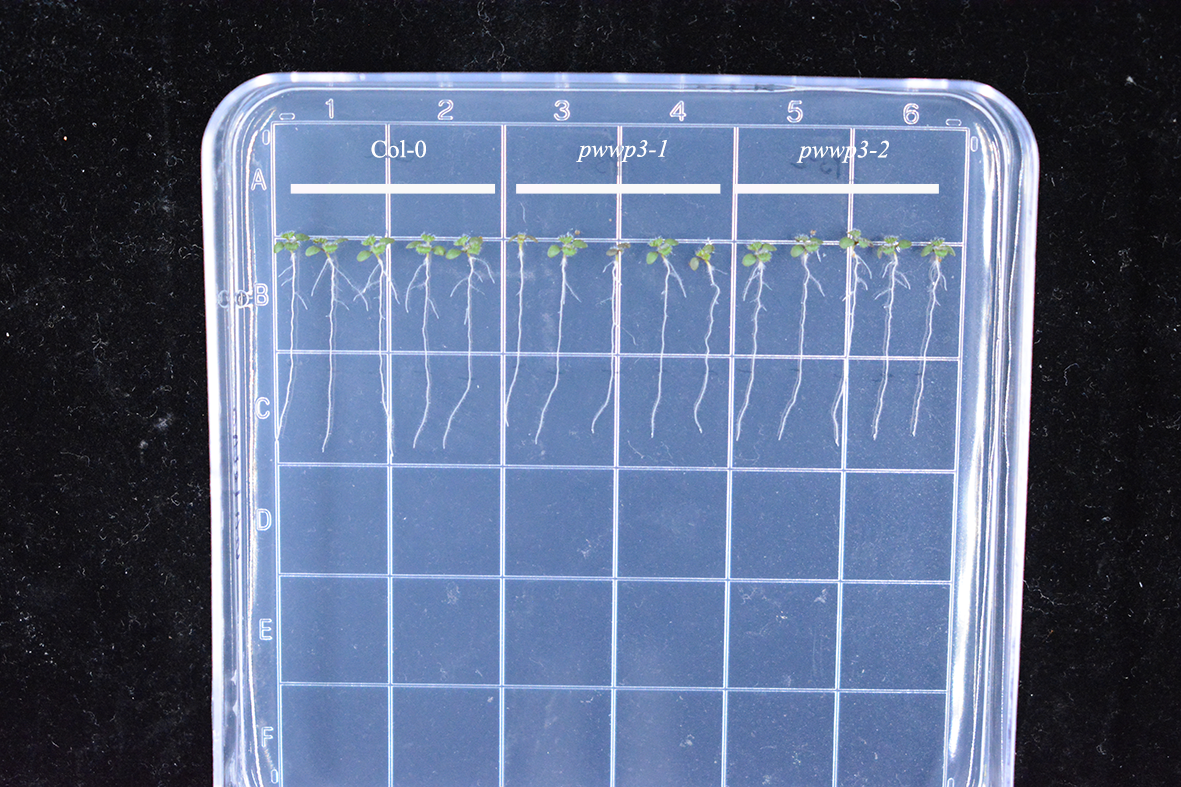

Supplement: Supplementary file 7 — Source Data Fig. 4 [file 44319_2023_30_MOESM7_ESM.zip › Fig.4E/20% PEG.tif]

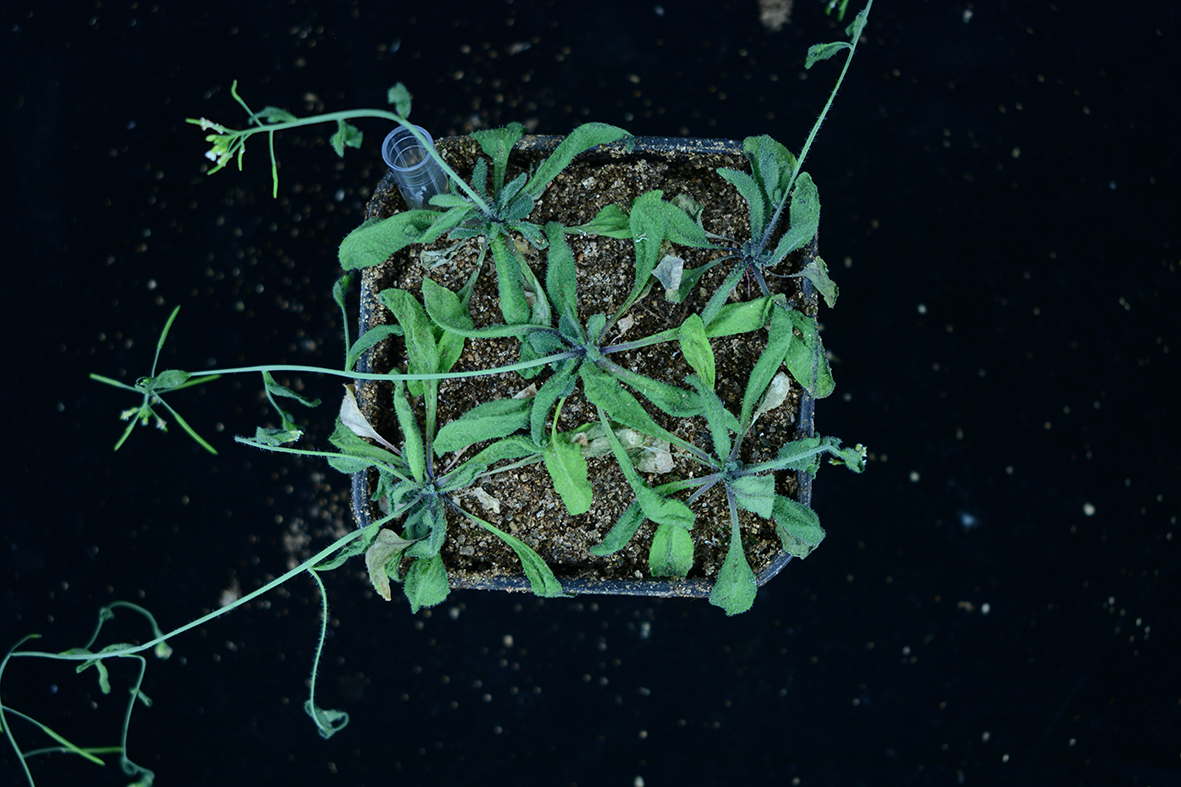

Supplement: Supplementary file 7 — Source Data Fig. 4 [file 44319_2023_30_MOESM7_ESM.zip › Fig.4G/Col-0 drought for thirteen days.tif]

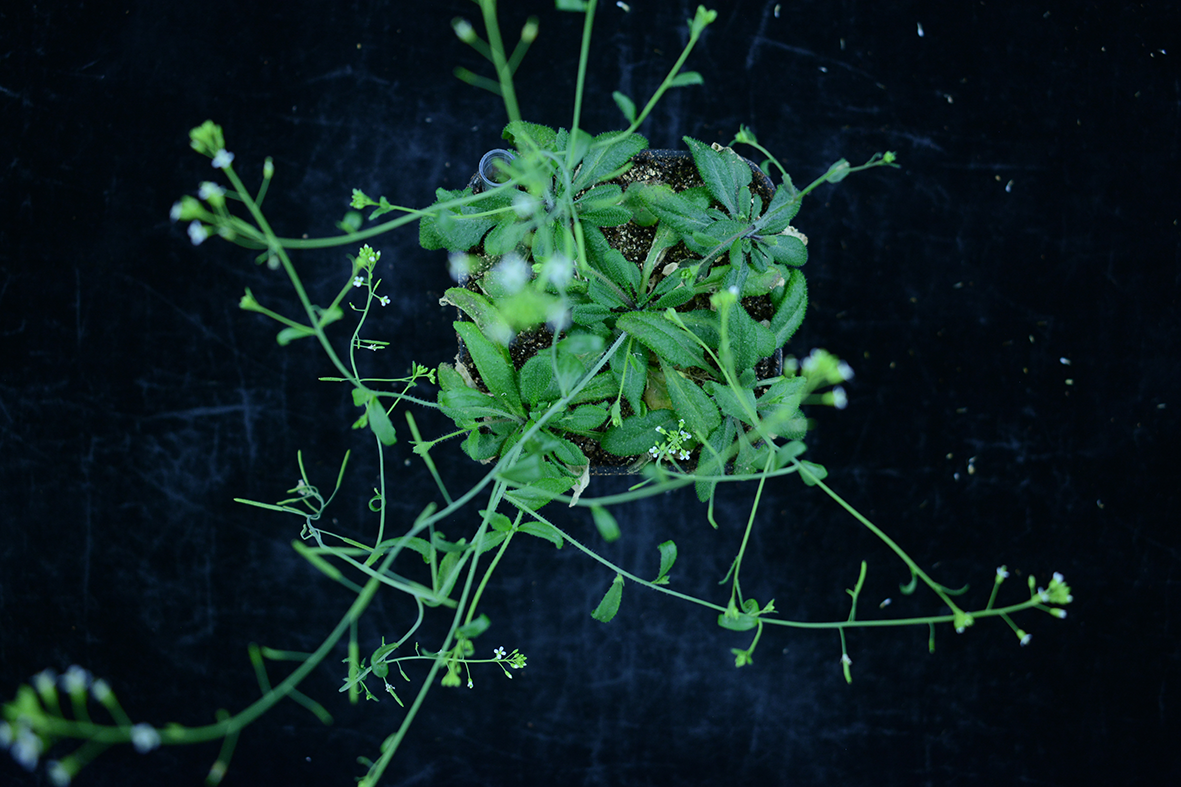

Supplement: Supplementary file 7 — Source Data Fig. 4 [file 44319_2023_30_MOESM7_ESM.zip › Fig.4G/Col-0 five days after rewatering.tif]

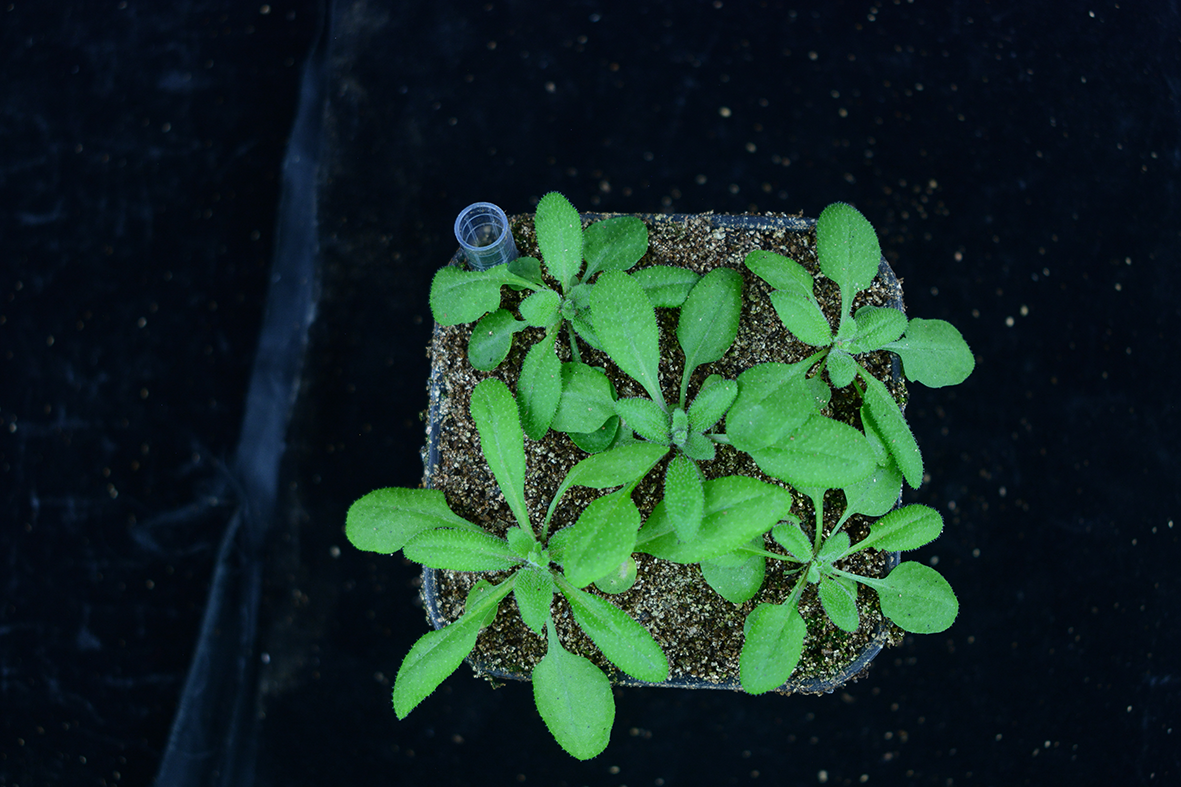

Supplement: Supplementary file 7 — Source Data Fig. 4 [file 44319_2023_30_MOESM7_ESM.zip › Fig.4G/Col-0 three-week-old seedlings.tif]

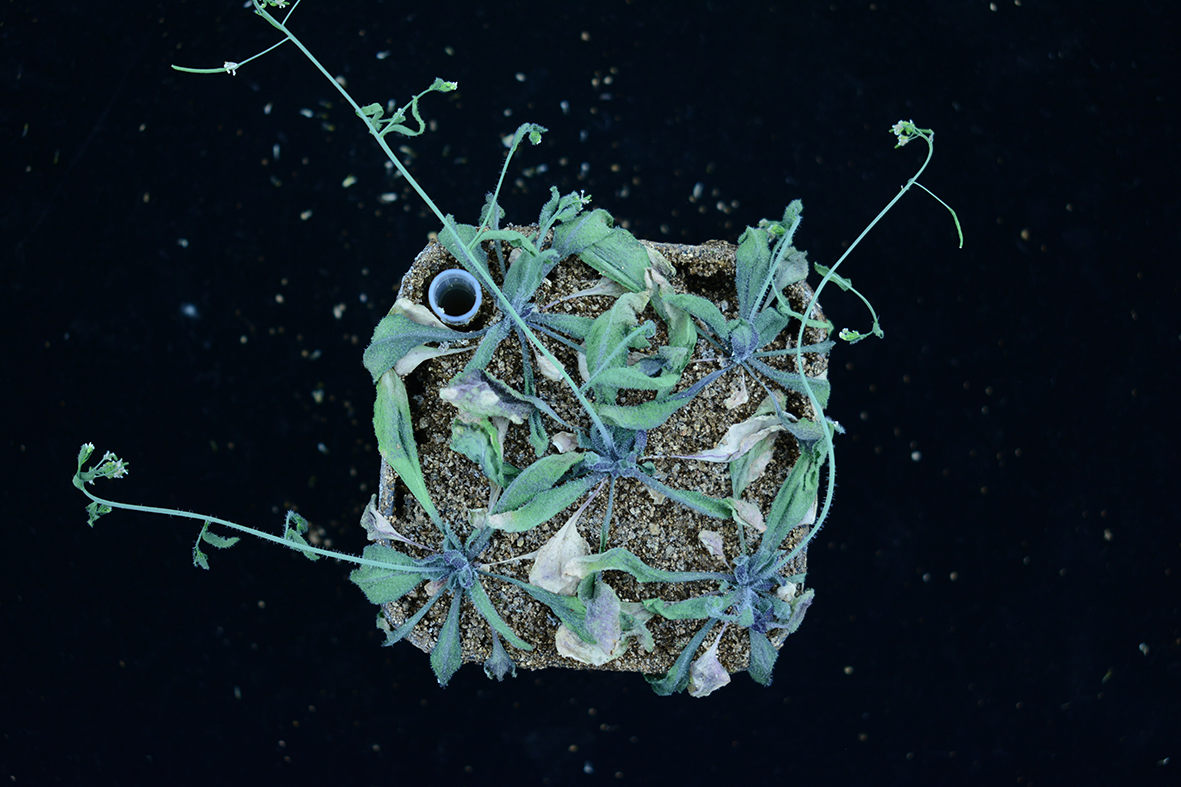

Supplement: Supplementary file 7 — Source Data Fig. 4 [file 44319_2023_30_MOESM7_ESM.zip › Fig.4G/pwwp3-1 drought for thirteen days.tif]

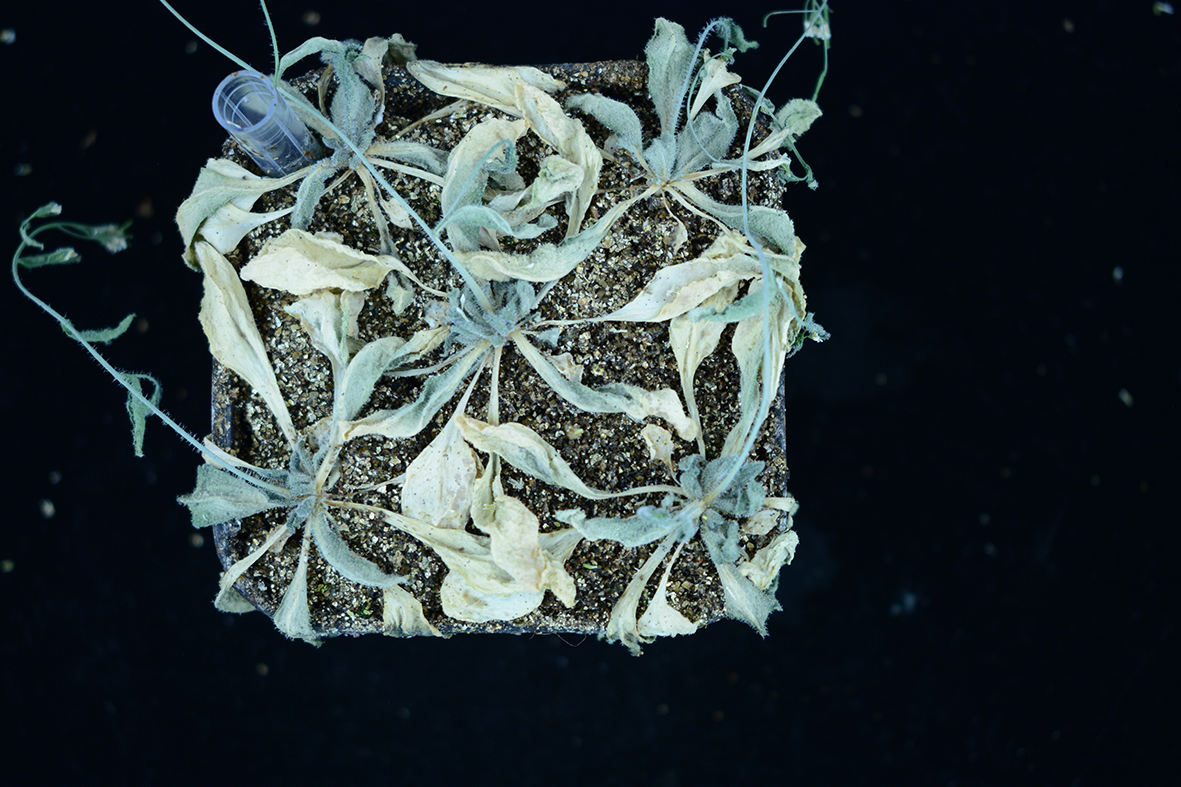

Supplement: Supplementary file 7 — Source Data Fig. 4 [file 44319_2023_30_MOESM7_ESM.zip › Fig.4G/pwwp3-1 five days after rewatering.tif]

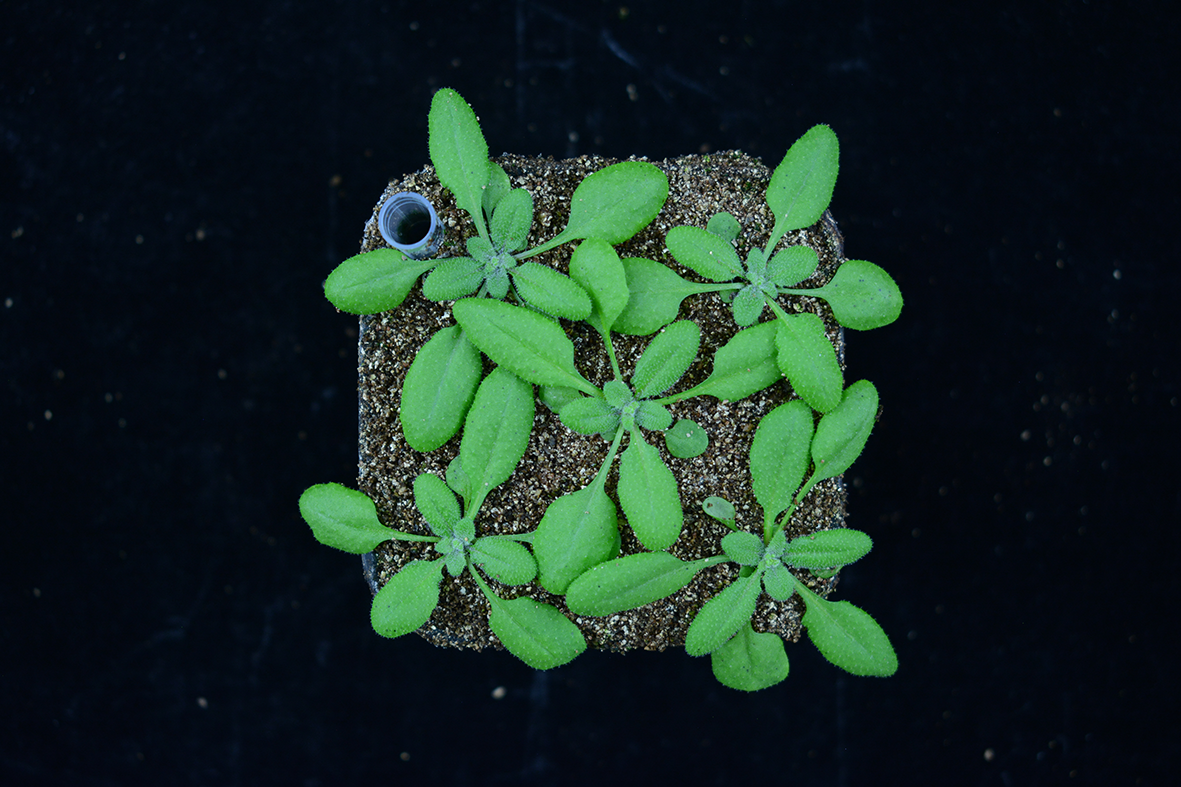

Supplement: Supplementary file 7 — Source Data Fig. 4 [file 44319_2023_30_MOESM7_ESM.zip › Fig.4G/pwwp3-1 three-week-old seedlings.tif]

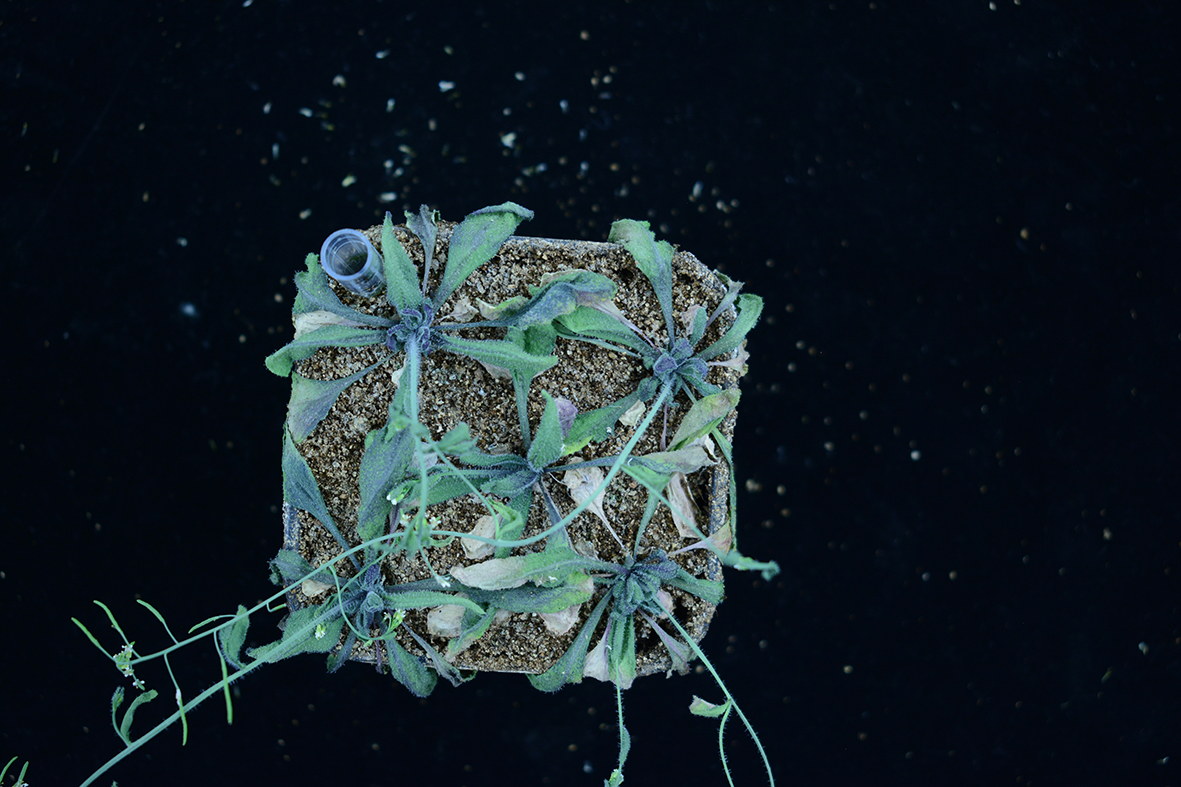

Supplement: Supplementary file 7 — Source Data Fig. 4 [file 44319_2023_30_MOESM7_ESM.zip › Fig.4G/pwwp3-2 drought for thirteen days.tif]

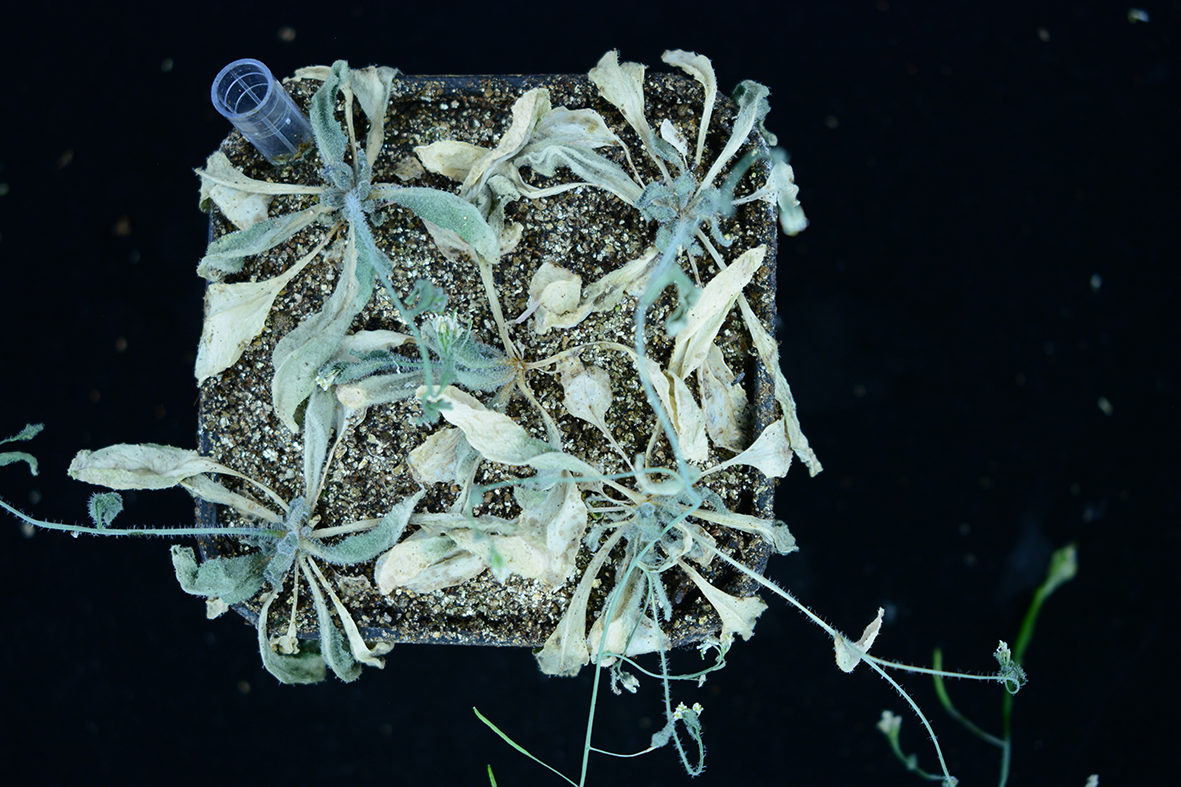

Supplement: Supplementary file 7 — Source Data Fig. 4 [file 44319_2023_30_MOESM7_ESM.zip › Fig.4G/pwwp3-2 five days after rewatering.tif]

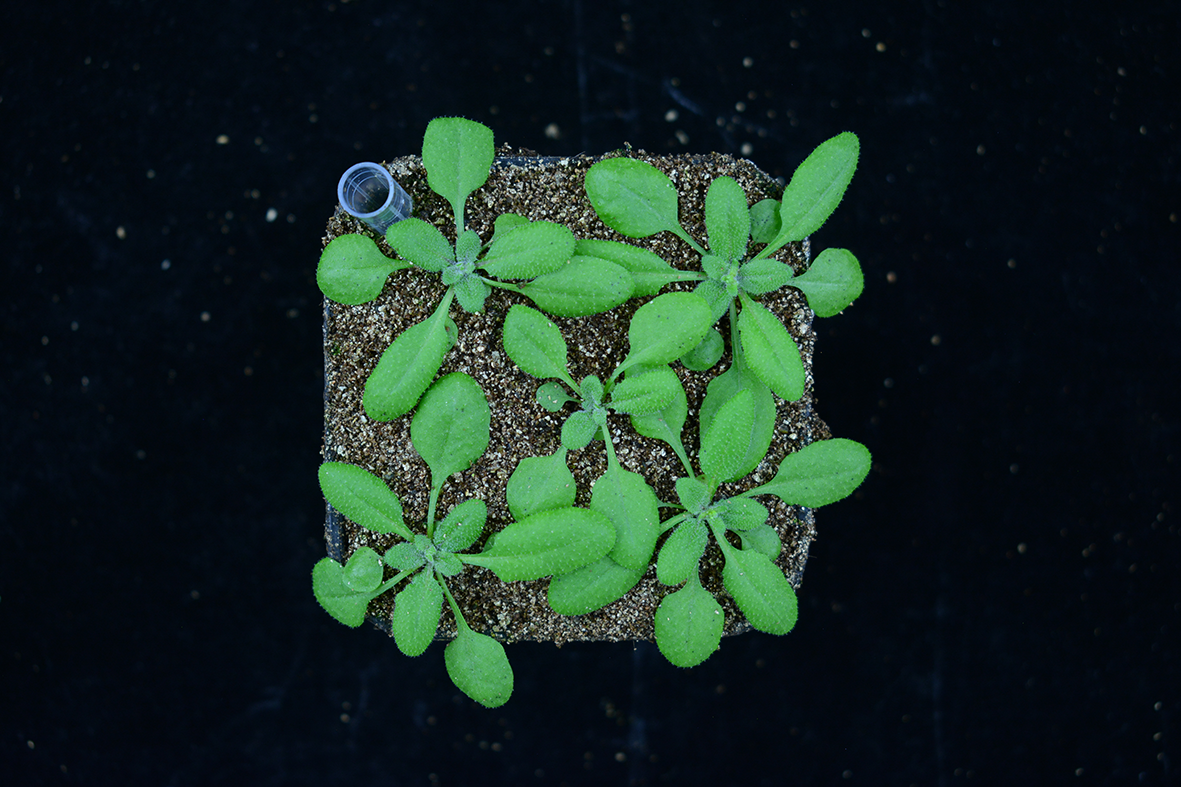

Supplement: Supplementary file 7 — Source Data Fig. 4 [file 44319_2023_30_MOESM7_ESM.zip › Fig.4G/pwwp3-2 three-week-old seedlings.tif]

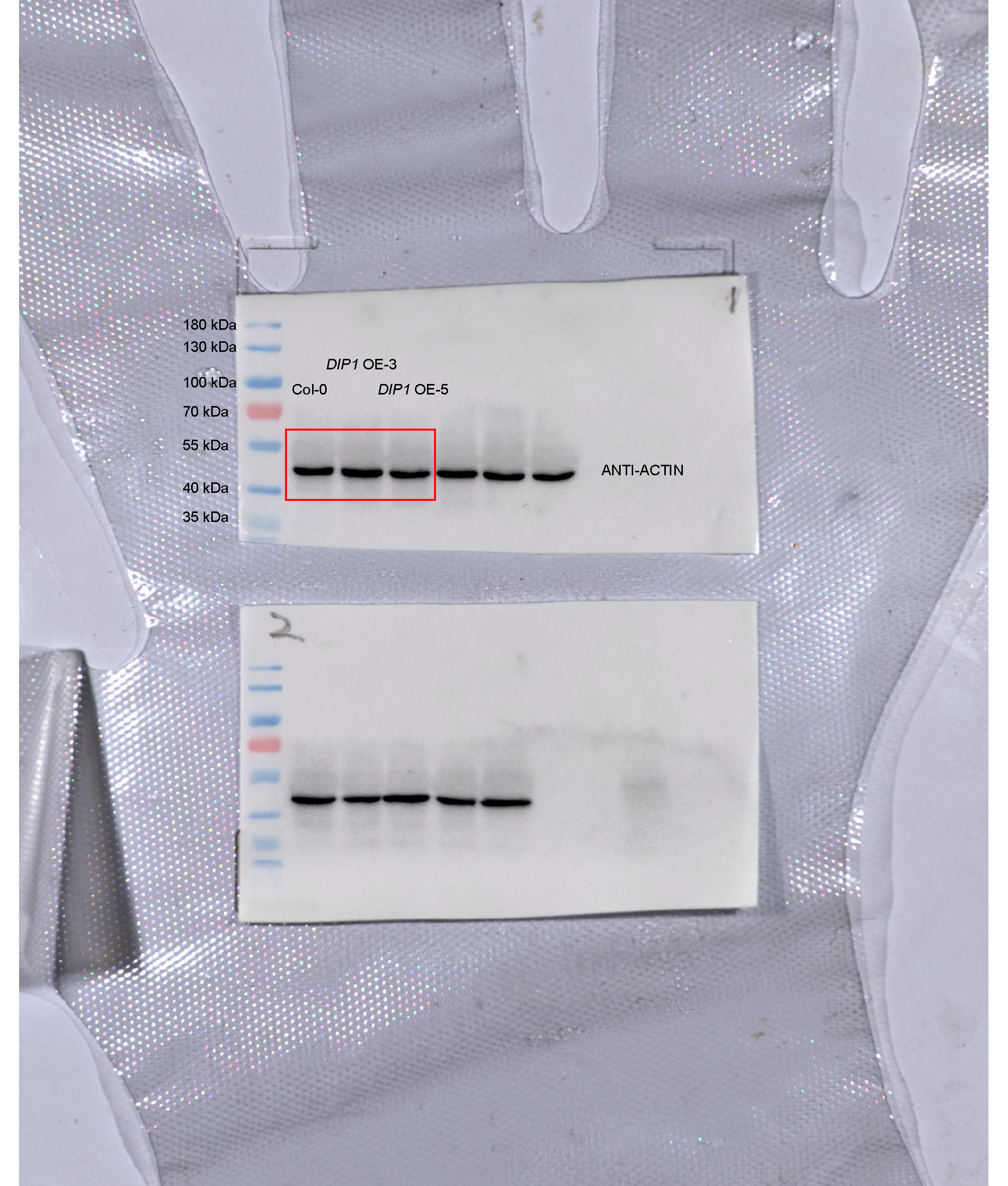

Supplement: Supplementary file 9 — Appendix Figure S6B Source Data [file 44319_2023_30_MOESM9_ESM.zip › ANTI-ACTIN.tif]

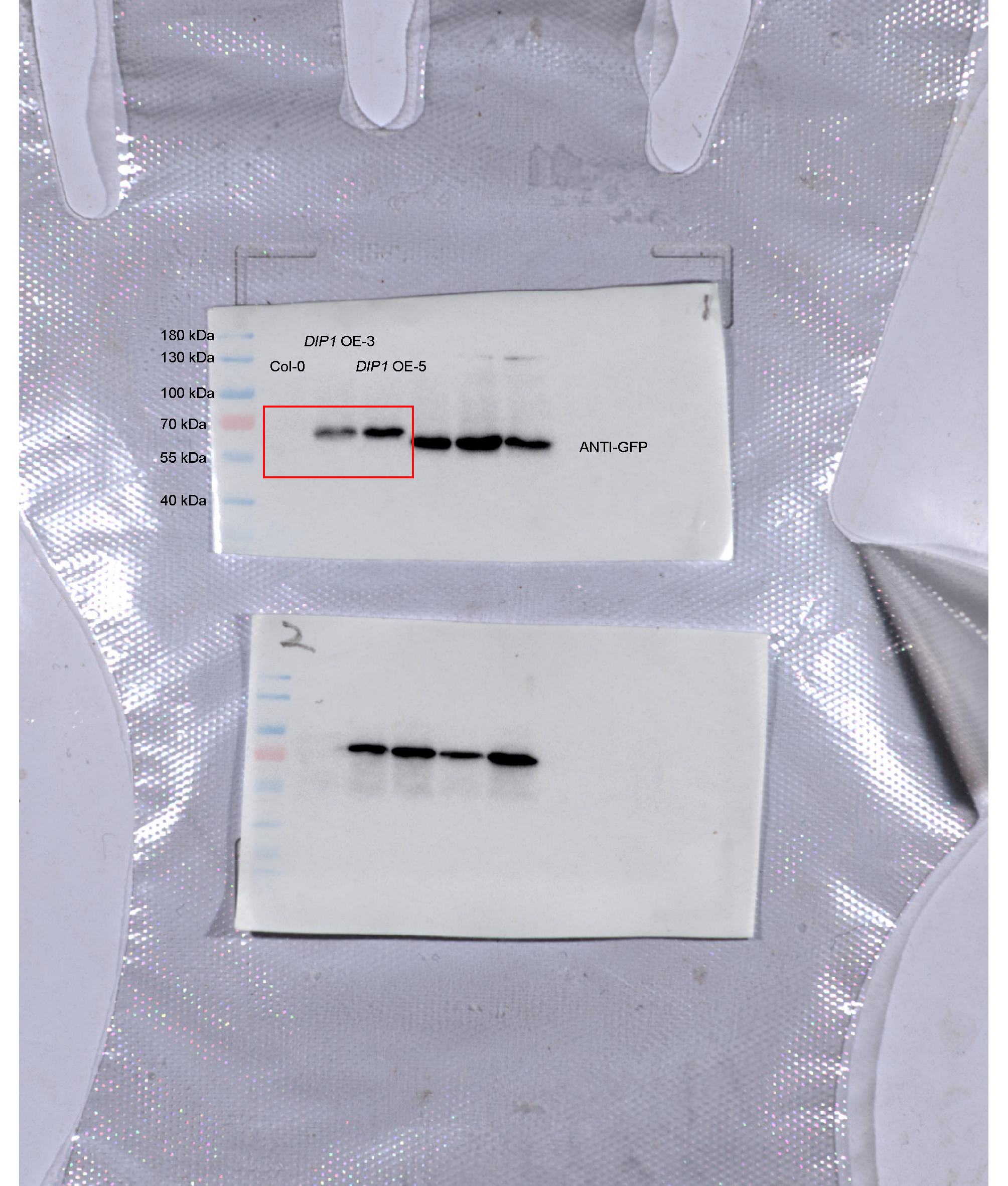

Supplement: Supplementary file 9 — Appendix Figure S6B Source Data [file 44319_2023_30_MOESM9_ESM.zip › ANTI-GFP.tif]
